# National tuberculosis spending efficiency and its associated factors in 121 low-income and middle-income countries, 2010–19: a data envelopment and stochastic frontier analysis

**DOI:** 10.1016/S2214-109X(22)00085-7

**Published:** 2022-04-12

**Authors:** Gerard Joseph Abou Jaoude, Ines Garcia Baena, Peter Nguhiu, Andrew Siroka, Tom Palmer, Lara Goscé, Kasim Allel, Edina Sinanovic, Jolene Skordis, Hassan Haghparast-Bidgoli

**Affiliations:** a Institute for Global Health, University College London, London, UK; b World Health Organization, Geneva, Switzerland; c Kenya Medical Research Institute (KEMRI) Wellcome Trust, Nairobi, Kenya; d Department of Disease Control, Faculty of Infectious & Tropical Diseases, London School of Hygiene & Tropical Medicine, London, UK; e Health Economics Unit, School of Public Health & Family Medicine, University of Cape Town, Cape Town, South Africa

## Abstract

**Background:**

Maximising the efficiency of national tuberculosis programmes is key to improving service coverage, outcomes, and progress towards End TB targets. We aimed to determine the overall efficiency of tuberculosis spending and investigate associated factors in 121 low-income and middle-income countries between 2010 and 2019.

**Methods:**

In this data envelopment and stochastic frontier analysis, we used data from the WHO Global TB report series on tuberculosis spending as the input and treatment coverage as the output to estimate tuberculosis spending efficiency. We investigated associations between 25 independent variables and overall efficiency.

**Findings:**

We estimated global tuberculosis spending efficiency to be between 73·8% (95% CI 71·2–76·3) and 87·7% (84·9–90·6) in 2019, depending on the analytical method used. This estimate suggests that existing global tuberculosis treatment coverage could be increased by between 12·3% (95% CI 9·4–15·1) and 26·2% (23·7–28·8) for the same amount of spending. Efficiency has improved over the study period, mainly since 2015, but a substantial difference of 70·7–72·1 percentage points between the most and least efficient countries still exists. We found a consistent significant association between efficiency and current health expenditure as a share of gross domestic product, out-of-pocket spending on health, and some Sustainable Development Goal (SDG) indicators such as universal health coverage.

**Interpretation:**

To improve efficiency, treatment coverage will need to be increased, particularly in the least efficient contexts where this might require additional spending. However, progress towards global End TB targets is slow even in the most efficient countries. Variables associated with TB spending efficiency suggest efficiency is complimented by commitments to improving health-care access that is free at the point of use and wider progress towards the SDGs. These findings support calls for additional investment in tuberculosis care.

**Funding:**

None.

## Introduction

Tuberculosis remains a leading cause of mortality and morbidity globally. In 2019, tuberculosis accounted for an estimated 1·4 million deaths and 47·0 million disability-adjusted life-years, with 42·7 million years of life lost and 4·3 million years lived with disability.^1^, ^2^ Over 90% of notified tuberculosis infections occur in low-income and middle-income countries and two-thirds of the estimated 10 million new active tuberculosis cases globally are accounted for by eight countries: India (26% of global cases), Indonesia (9%), China (8%), the Philippines (6%), Pakistan (6%), Nigeria (4%), Bangladesh (4%), and South Africa (4%).^1^ Despite a 14% cumulative reduction in global tuberculosis-related mortality since 2015 and a 2% average annual decrease in incidence, global progress is falling short of improvements required to meet targets set by the WHO End TB Strategy and the UN Sustainable Development Goal on tuberculosis (SDG 3.3).^1^, ^3^, ^4^

Tuberculosis spending in low-income and middle-income countries has been increasing since 2006.^1^ However, WHO estimates that tuberculosis spending decreased from US$5·8 billion in 2019 to $5·3 billion in 2020.^5^ Hence, spending in 2020 amounted to less than half of the $13 billion in funding required for the Stop TB Partnership's Global Plan to End TB, 2018–22, in line with funding targets laid out in the UN high-level meeting on the fight for tuberculosis in 2018.^1^, ^6^, ^7^ To minimise tuberculosis-related mortality and morbidity, the gap in resources must be closed by mobilising additional funding sources^1^ and maximising the efficiency of tuberculosis spending.^8^ To our knowledge, only one multicountry analysis of the overall efficiency of national tuberculosis spending has been done to date;^9^ however, this analysis included only 19 countries for the period 2011–17. Here, we aimed to build on existing evidence with several analyses of tuberculosis spending efficiency and its associated factors in 121 low-income and middle-income countries between 2010 and 2019.

**Research in context**

**Evidence before this study**

Few multicountry efficiency studies of health spending have been published before this study, including an analysis of HIV/AIDS spending in 68 low-income and middle-income countries between 2002 and 2007 published in 2012. More recently, a study published in 2019 investigated the health system efficiency of 46 countries in Asia and two other studies published in 2020 investigated the efficiency of spending on universal health coverage (UHC) outputs in 204 countries and 172 countries. We searched PubMed, Scopus, and Google Scholar on Nov 24, 2021, with no date restrictions, for publications in English, using the terms “technical efficiency” and synonyms combined with “tuberculosis” to identify multicountry efficiency studies of national tuberculosis programmes or spending. We only identified one study, published in 2019, which considered 19 countries and two independent variables.

**Added value of this study**

To our knowledge, this is the first comprehensive global analysis of the overall efficiency of tuberculosis spending. We investigated tuberculosis spending efficiency in 121 low-income and middle-income countries between 2010 and 2019 along with 25 independent variables. The combined use of data envelopment analysis (DEA) and stochastic frontier analysis (SFA) to estimate efficiency in this study built on the literature more widely than previous studies. To our knowledge, this study is one of few multicountry analyses of health spending efficiency to use both DEA and SFA models to estimate efficiency.

**Implications of all the available evidence**

We estimated that global tuberculosis treatment coverage could be increased by between 12·3% and 26·2% from existing levels, with the same amount of spending. Substantial variations in efficiency between countries are driven by tuberculosis treatment coverage rather than spending. Therefore, countries should focus on increasing treatment coverage even if this requires additional spending. However, there is insufficient progress towards End TB targets even in the most efficient countries and some potential sources of inefficiency are beyond the control of national tuberculosis programmes. Regression analysis results suggest that government commitment to health, lower out-of-pocket spending on health and tuberculosis, and progress towards key Sustainable Development Goal indicators such as UHC or reducing the proportion of people living in slums, are associated with increased levels of tuberculosis programming efficiency. Overall, our findings support existing evidence calling for additional investment in tuberculosis care and multisectoral action to meet End TB targets.

## Methods

### Study design and analytical models

In this study, we used two analytical methods, data envelopment and stochastic frontier analysis, to model tuberculosis spending efficiency for 121 low-income and middle-income countries using data from the Global TB Report series.

Overall efficiency considers how different production units, or decision-making units, perform in maximising outputs from related inputs.^10^, ^11^ Fully efficient decision-making units lie on an efficiency frontier. In this study, we designated countries and their national tuberculosis programmes as the decision-making units. We used data envelopment analysis (DEA) and stochastic frontier analysis (SFA) to determine the efficiency frontier. DEA and SFA are widely applied in health-care analyses to estimate the efficiency of units ranging from facilities to national health systems.^12^, ^13^ DEA is a non-parametric method and requires few assumptions to construct the efficiency frontier,^11^ whereas SFA is a parametric method and can allow considerations such as heterogeneity in the data as well as investigating factors associated with inefficiency.^14^

### Input and output variables

We compiled national tuberculosis spending data in constant 2020 US$ using WHO estimates published in the Global TB report series.^15^ WHO tuberculosis finance database estimates include public spending reported by national tuberculosis programmes from domestic public and donor sources. Funding for inpatient and outpatient care are estimated independently by WHO. Out-of-pocket and pre-paid private spending are not included. Sufficient data were available to include spending for fiscal years between 2010 and 2019 for 121 low-income and middle-income countries, which account for 98% of global tuberculosis notifications.^1^ We divided annual national tuberculosis spending by the annual number of people notified with tuberculosis to generate the main DEA and SFA input, estimate tuberculosis spending per person notified, and enable comparability across decision-making units. In total, 121 countries over the 10-year period produced 1209 observations (one of the countries included in the analysis, South Sudan, was founded in 2011), including all 30 countries with a high tuberculosis burden and every WHO region (43 countries in the African region, 20 in the region of the Americas, 13 in the Eastern Mediterranean region, 16 in the European region, 11 in the South-East Asia region, and 18 in the Western Pacific region) and World Bank income group except for high-income countries (28 low-income countries, 46 lower-middle-income countries, and 47 upper-middle-income countries; appendix p 11–36).

We used annual tuberculosis treatment coverage as the main DEA and SFA output. Treatment coverage was defined as total cases notified as a share of total incident cases. We derived these data from estimates published in the Global TB Report series,^15^ and they were available for all for all countries for all years investigated. We verified isotonicity (ie, that increases in inputs result in increases in outputs) between input and output variables (ρ*=*0·230, p<0·0001), which is required for DEA and SFA. Summary statistics for input and output variables are presented in table 1.

**Table 1:** Summary statistics of input, output, and independent variables across 121 low-income and middle-income countries over a 10 year period used in the main data envelopment and stochastic frontier analyses

			**Mean (SD)**	**Minimum to maximum**
Input				
	Spending per person notified with tuberculosis (constant in 2020 US$)		$3845·54 (9937·72)	$54·65 to $168 076·10
Output				
	Treatment coverage (defined as yearly notifications divided by incidence)		68·83 (16·48)	22 to 110
Independent variables (n=25)				
	Drug-sensitive tuberculosis			
		Typical number of visits to a health facility after diagnosis for treatment	63·66 (67·66)	0·00 to 448·00
		Estimated proportion of cases who are hospitalised	27·38% (31·16)	0% to 100%
		Estimated average duration of hospital stay if cases are hospitalised, days	26·14 (23·14)	0·00 to 120·00
	Multidrug-resistant tuberculosis			
		Typical number of visits to a health facility after diagnosis for treatment	216·04 (239·95)	0·00 to 810·00
		Estimated proportion of cases who are hospitalised	62·92% (40·72)	0% to 100%
		Estimated average duration of hospital stay if cases are hospitalised, days	103·96 (98·75)	0·00 to 630·00
Tuberculosis spending accounted for by external sources (proportion of total public tuberculosis spending, excluding private spending)			37·46% (30·75)	0% to 99·87%
Governance indicator*			−0·54 (0·58)	−2·31 to 0·95
Population total			48 800 000 (173 000 000)	10 530 to 1 400 000 000
Population per km^2^			125·24 (196·31)	1·75 to 1769·84
Rural population (proportion of total population)			51·09% (20·76)	8·01% to 89·36%
Current health expenditure (proportion of GDP)			6·13% (2·77)	0·69% to 20·41%
Current health expenditure per capita, PPP (current international $)			$467·89 (440·25)	$22·27 to $3142·44
External health expenditure (proportion of current health expenditure)			13·89% (16·85)	0% to 79·98%
Out-of-pocket expenditure (proportion of current health expenditure)			37·57% (20·57)	0·11% to 85·26%
UHC service coverage index			56·29 (14·27)	22·00 to 83·00
Incidence of tuberculosis per 100 000 people			184·25 (195·40)	1·90 to 1590·00
Incidence of multidrug-resistant tuberculosis per 100 000 people			6·45 (8·17)	0·02 to 49·77
HIV prevalence (adults aged 15–49 years; proportion of population)			2·13% (4·75)	0·10% to 28·90%
Population living in slums (proportion of urban population)			41·20% (22·61)	3·60% to 97·50%
Diabetes prevalence (proportion of population age ≥18 years)			9·86% (4·28)	3·70% to 27·33%
Alcohol use disorders, 12-month prevalence (proportion of population age ≥15 years)			4·76% (3·16)	0·40% to 20·90%
Gini index†			40·76 (7·81)	24·00 to 63·40
Proportion of population living below the international poverty line			20·18% (21·57)	0% to 79·00%
Prevalence of undernourishment (proportion of population)			15·44% (12·02)	2·11% to 72·89%

All variables were determined on the basis of 1209 observations. GDP=gross domestic product. PPP=purchasing power parity. UHC=universal health coverage.

* The average of six World Governance Indicators: (1) voice and accountability, (2) political stability and absence of violence or terrorism, (3) government effectiveness, (4) regulatory quality, (5) rule of law, and (6) control of corruption.

† In which a score of 0 equates to perfect equality and 100 equates to perfect inequality.

### Independent variables

We selected independent variables on the basis of previous studies,^16^, ^17^, ^18^ their interaction with tuberculosis care and services, and the availability of data. The expected effect of independent variables on efficiency, justification for their inclusion, and data sources are in the appendix (pp 2–4). Summary statistics for the 25 independent variables included are shown in table 1. Substantial amounts of data were missing for SDG indicators considered. Details on excluded SDG indicators and how missing data were addressed for those included are in the appendix (p 5). The following SDG indicators were included: population living in slums (proportion of urban population), diabetes prevalence (proportion of population aged ≥18 years), alcohol use disorders, 12-month prevalence (proportion of population age ≥15 years), Gini index, proportion of population living below the international poverty line, prevalence of undernourishment (proportion of population), HIV prevalence (in individuals aged 15–49 years; proportion of population), incidence of tuberculosis per 100 000 people, and universal health coverage (UHC) service coverage index. Correlation between selected independent variables does not suggest a risk of multicollinearity (mean variance inflation factor was 2·48 [appendix p 6]). We used Pesaran's test of cross-sectional dependence^19^ and we found that the results reject the presence of strong cross-sectional dependence that could cause inconsistent or biased estimates (fixed effects: Pesaran*=*1·502, probability=0·1331; random effects: Pesaran=0·681, probability=0·4961).

### Estimating overall efficiency

DEA and SFA are used here to construct an efficiency frontier. The frontier represents the maximum outputs that can be produced from inputs. Efficiency scores represent the distance of countries from the constructed frontier. An efficiency score of 0 is the furthest from the frontier, while an efficiency score of 100 indicates that the country of interest lies on the efficiency frontier.

We used a double bootstrap two-stage DEA method, developed by Simar and Wilson,^20^ to correct for serial correlation and endogeneity due to measurement error, which other two-stage DEA approaches are prone to. The Simar–Wilson DEA method adjusts initial efficiency estimates to generate bias-corrected efficiency scores by correcting for bias caused by serial correlation or measurement error. We chose an output-oriented model, in line with previous studies,^16^, ^17^, ^18^, ^21^ in which the efficiency frontier represents the maximum outputs that can be produced from inputs. Defining a frontier that represents maximising outputs reflects a global commitment to maximise outputs, such as tuberculosis treatment coverage, rather than minimise resources while maintaining existing outputs, and the fact that national tuberculosis programmes have less control over their allocated budgets than their outputs. Because national tuberculosis programmes use various inputs to produce multiple outputs, we assumed variable returns to scale to generate the main findings,^18^, ^22^ such that outputs might increase or decrease with increases in inputs. We used the true-random effects model^14^, ^23^ for the main SFA and we assumed a half-normal distribution and random effects.^24^

Bootstrapping in the first stage of DEA after truncated maximum likelihood estimation generates a better approximation of the true sampling distribution when repeated a number of times. In this study, after 3000 bootstrap replications, correlation between the initial Shepard efficiency scores and bias-corrected efficiency scores were 0·998 (p<0·0001). We used bootstrapped truncated maximum likelihood estimation, again with 3000 replications, in the second stage of DEA to investigate factors associated with overall efficiency and generate unbiased coefficients and confidence bounds. For the SFA, we estimated robust coefficients using simulated maximum likelihood. We also used a joinpoint regression analysis, which assumes trend data can be divided into sections and unique trends determined in each section, to determine whether significant changes existed in the trend of efficiency estimates (DEA and SFA) between 2010 and 2019. We investigated the correlation between efficiency estimates and rankings across DEA and SFA models, and between input and output variables with main DEA and SFA model efficiency estimates. We did all analyses using Stata (version 15.0).^23^, ^25^

### Sensitivity analysis

To investigate the robustness of estimated efficiency scores, we did sensitivity analyses in which we tested 13 variations of the main DEA and SFA models. One disadvantage of DEA and SFA is their high sensitivity to outliers.^26^ Hence, three of 13 alternative models tested the effect on efficiency scores of excluding different combinations of outliers. Four models tested the effect of using different combinations of outputs and independent variables used in the second stage regression. Different combinations of outputs included outcome-adjusted treatment coverage of new drug-susceptible tuberculosis, previously treated drug-susceptible tuberculosis cases, and multidrug-resistant tuberculosis cases. We generated these outputs by multiplying Global TB Report series data^15^ on treatment coverage with the annual proportion of treatment success for people with new drug-susceptible tuberculosis, previously treated drug-susceptible tuberculosis, and multidrug-resistant tuberculosis. Three other models investigated the effect of considering lag in investment impact using a 3-year and 5-year time lag^17^ and a 10-year average of all variables with a cross-sectional design. Another model assumed constant returns to scale, which were used to calculate scale efficiency by dividing the results with variable returns to scale efficiency scores. We also investigated two SFA models based on models by Pitt and Lee^27^ and Battese and Coelli.^23^, ^28^ Finally, we did four subgroup DEA analyses to enable comparison of efficiency and coefficient estimates with the main DEA model. The four subgroups included all three World Bank income groups and the WHO African region. Other regions are not presented here due to multicollinearity.

### Role of the funding source

There was no funding source for this study.

## Results

**Figure 1:**
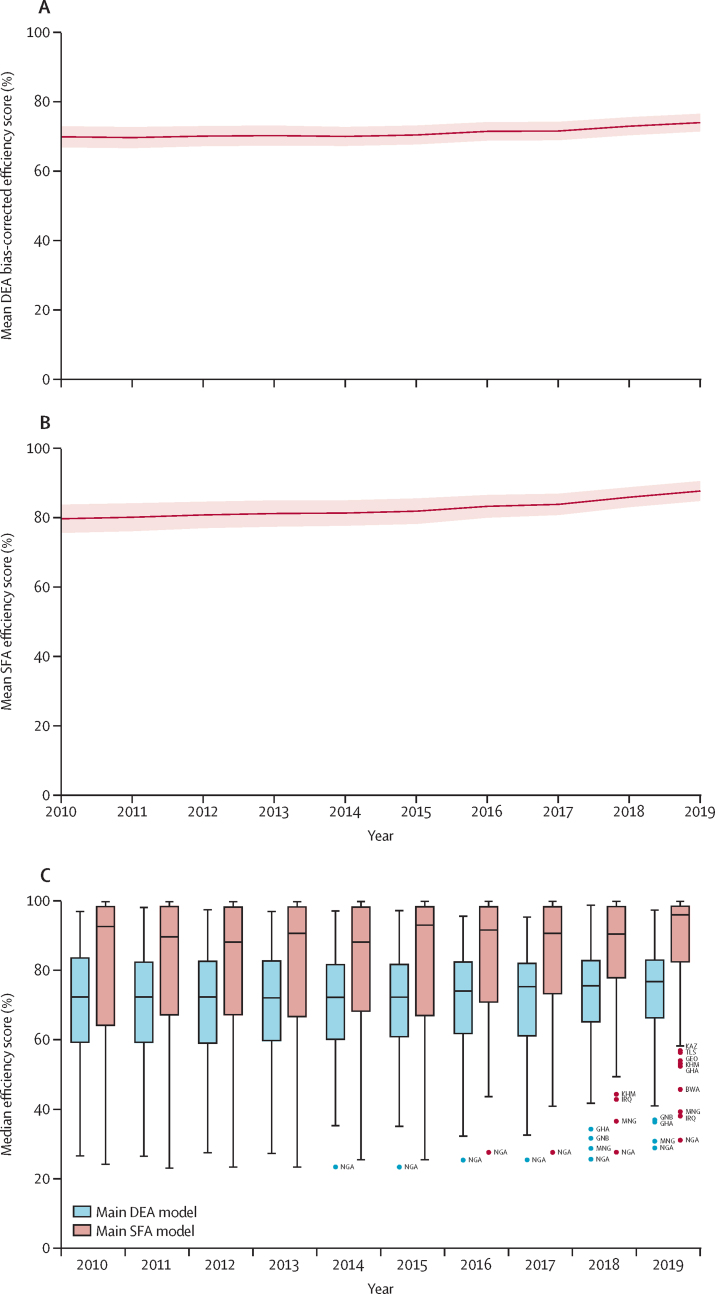
Average DEA and SFA efficiency score for the period 2010–19 (A) Mean DEA bias-corrected efficiency score. (B) Mean SFA efficiency score. Solid lines show means, and shaded area shows 95% CIs. (C) Box plot of median DEA and SFA efficiency scores for each year, with the central line of the box showing the median, the extremes of the box showing the IQR, the whiskers showing the range of scores, and datapoints showing the outliers. BWA=Botswana. DEA=data envelopment analysis. GEO=Georgia. GHA=Ghana. GNB=Guinea-Bissau. IRQ=Iraq. KAZ=Kazakhstan. KHM=Cambodia. MNG=Mongolia. NGA=Nigeria. SFA=stochastic frontier analysis. TLS=Timor-Leste.

We estimated mean tuberculosis spending efficiency to have improved from 69·7% (95% CI 66·7–72·7) in 2010 to 73·8% (71·2–76·3) in 2019 across 121 low-income and middle-income countries on the basis of the DEA model, and from 79·7% (75·7–83·8) in 2010 to 87·7% (84·9–90·6) in 2019 on the basis of the SFA model (figure 1). Therefore, countries could have increased treatment coverage by between 12·3% (95% CI 9·4–15·1) and 26·2% (23·7–28·8) on average, compared with actual levels, for the same amount of spending in 2019. The sharpest increase in efficiency occurred between 2015 and 2019, during which mean annual increases in efficiency across countries were between 0·89 percentage points (pp; DEA) and 1·45 pp (SFA). By comparison, the average annual efficiency increase between 2010 and 2015 was between 0·1 pp (DEA) and 0·44 pp (SFA; figure 1). Correlation between the initial Shepard efficiency scores and bias-corrected efficiency scores were 0·998 (p<0·0001).

The range of efficiency scores narrowed over time (figure 1C), converging across countries towards higher levels of efficiency relative to baseline (2010). We found a significant break in the trend of DEA estimates between 2014 and 2015 (specifically at 2014·8; p<0·0001, adjusted *R*^2^=0·0054) and a weakly significant increasing trend between 2015 and 2019 (0·86% increase; p=0·051). For the SFA estimates, a significant break in the trend was found between 2015 and 2016 (specifically at 2015·5; p<0·0001, adjusted *R*^2^=0·0132 and a weakly significant increasing trend between 2016 and 2019 (1·53%; p=0·053). According to both DEA and SFA estimates, trends in efficiency by World Bank income group and WHO region were similar to the overall trend observed across all countries, except for the South-East Asia region and low-income countries, for which efficiency increased substantially more than the average increase across all countries (appendix pp 8–9).

**Figure 2:**
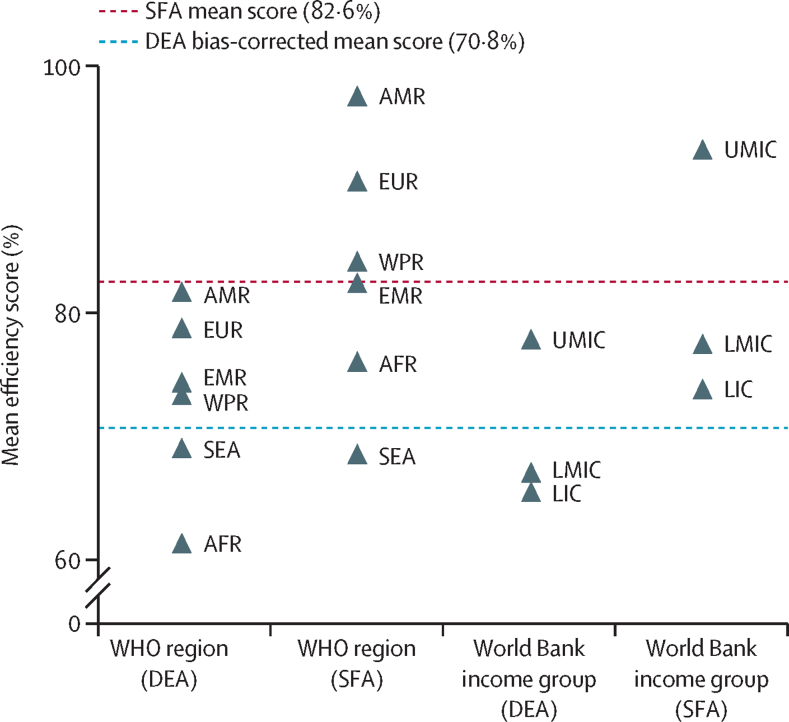
Mean DEA and SFA efficiency scores by income group and region, for the period 2010–19 AFR=African region. AMR=region of the Americas. DEA=data envelopment analysis. EMR=Eastern Mediterranean region. EUR=European region. LIC=low-income country. LMIC=lower-middle-income country. SEA=South-East Asia region. SFA=stochastic frontier analysis. UMIC=upper-middle-income country. WPR=Western Pacific region.

Differences in mean efficiency are greater between WHO regions and World Bank income groups than across years (Figure 1, Figure 2). On average, for the period 2010–19, upper-middle-income countries (DEA 77·8% [95% CI 76·7–78·8]; SFA 93·1% [91·8–94·3]) were on average 11–19 pp more efficient than lower-middle-income (DEA 67·0% [65·5–68·5]; SFA 77·4% [75·5–79·3]) and low-income countries (DEA 65·5% [63·7–67·2]; SFA 73·7% [71·7–75·8]). Countries in the region of the Americas (DEA 81·6% [95% CI 80·7–82·5]; SFA 97·4% [96·7–98·0]) and European regions (DEA 78·7% [77·6–79·7]; SFA 90·5% [88·2–92·8]) converted spending into treatment coverage most efficiently for the period 2010–19. The Eastern Mediterranean (DEA 74·3% [71·5–77·1]; SFA 82·3% [79·0–85·6]) and Western Pacific (DEA 73·3% [70·7–75·8]; SFA 84·0% [80·9–87·1]) regions were the next most efficient, with similar levels of efficiency for the period 2010–19. The South-East Asia region (DEA 69·0% [66·3–71·6]; SFA 68·5% [64·0–73·0]) and African region (DEA 61·3% [60·0–62·6]; SFA 76·0% [74·2–77·7]) were the least efficient on average between 2010 and 2019. The African region was the least efficient WHO region in the DEA analysis and the South-East Asia region in the SFA analysis.

**Figure 3:**
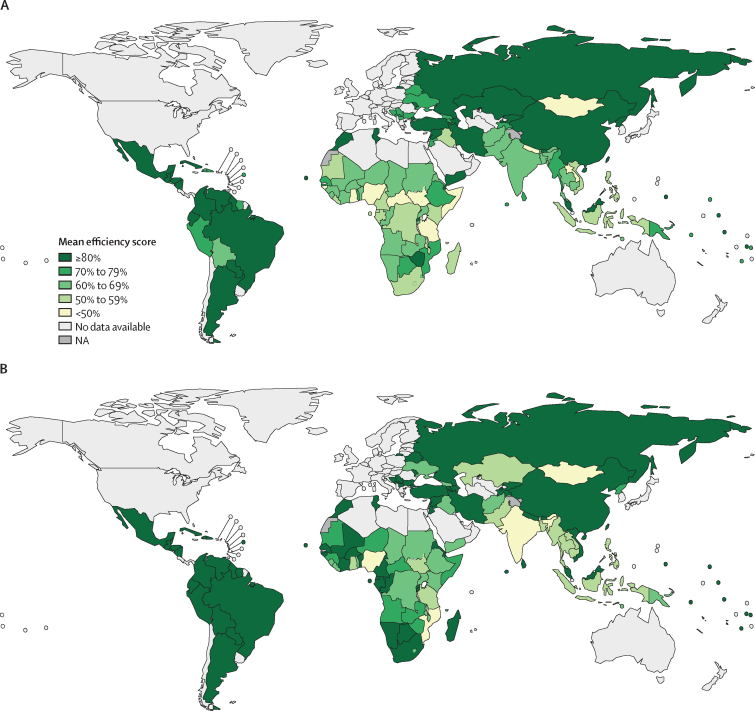
Global map of mean DEA bias-corrected (A) and SFA (B) efficiency scores by country, for the period 2010–19 DEA=data envelopment analysis. NA=not applicable. SFA=stochastic frontier analysis.

**Figure 4:**
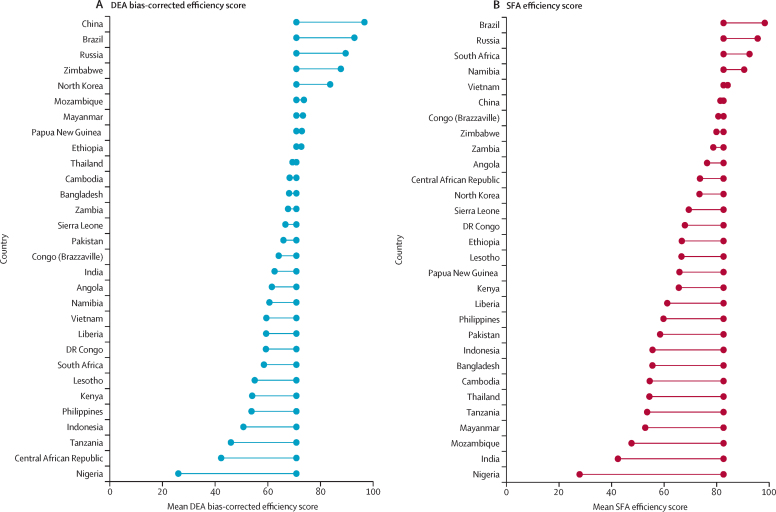
Mean DEA bias-corrected (A) and SFA (B) efficiency scores for 30 high tuberculosis burden countries for the period 2010–19 Centrally aligned datapoints show the average DEA bias-corrected efficiency score (70·8%) and SFA efficiency score (82·6%) across all 121 countries between 2010 and 2019, with datapoints to either side showing the score for each country. DEA=data envelopment analysis. SFA=stochastic frontier analysis.

The efficiency of national tuberculosis spending varies most between countries (figure 3). On average, we estimated a difference of 70·7 pp by DEA and 72·1 pp by SFA between the most and least efficient countries for the period 2010–19. Average efficiency between 2010 and 2019 also varied substantially among the 30 high tuberculosis burden countries (figure 4). In the DEA, nine of the 30 high tuberculosis burden countries (China, Brazil, Russia, Zimbabwe, North Korea, Mozambique, Myanmar, Papua New Guinea, and Ethiopia) were more efficient in their tuberculosis spending than the average estimated across all countries investigated, and in the SFA, five countries were more efficient than the average estimated across all countries (Brazil, Russia, South Africa, Namibia, and Vietnam). Country rankings, from most to least efficient, varied between the main DEA and SFA results (appendix p 10). Nonetheless, we found a significant moderate correlation between DEA and SFA country rankings (ρ=0·5799; p<0·0001) and a significant strong correlation between DEA and SFA efficiency scores (ρ=0·6584; p<0·0001; appendix p 7). Specific countries were consistently the most efficient across analyses (appendix p 39), such as Brazil (DEA 92·86%, SFA 98·33%), Paraguay (DEA 88·76%, SFA 98·29%), Venezuela (DEA 87·90%, SFA 99·59%), Rwanda (DEA 85·25%, SFA 96·13%), Lebanon (DEA 85·48%, SFA 98·35%), and Iran (DEA 83·97%, SFA 99·75%); while others remained among the least efficient, such as Nigeria (DEA 26·03%, SFA 27·84%), Mongolia (DEA 34·82%, SFA 43·73%), Ghana (DEA 34·91%, SFA 50·46%), Laos (DEA 43·07%, SFA 57·12%), and Indonesia (DEA 50·73%, SFA 55·62%). Full results including DEA (variable and constant returns to scale) efficiency scores, bias estimates, bias-corrected efficiency scores, scale efficiency, and SFA efficiency scores for all 1209 observations are in the appendix (pp 11–36).

Tuberculosis spending is weakly correlated with DEA (ρ=0·073; p=0·0108) and SFA (ρ=0·203; p<0·0001) efficiency estimates compared with tuberculosis treatment coverage, which is strongly correlated with both DEA (ρ=0·946; p<0·0001) and SFA (ρ=0·712; p<0·0001) efficiency estimates (for plots of tuberculosis spending against tuberculosis treatment coverage, similar variations are observed for outcome-adjusted coverage; appendix p 38). For example, average spending in constant 2020 US$ per notified tuberculosis case in the 10% of countries that are the most efficient is $3005 compared with $1240 in the 10% of countries that are the least efficient, but average treatment coverage is 87·3% in the 10% of countries that are the most efficient and 38·7% in the 10% of countries that are the least efficient. Additionally, on average, spending decreased by $255 annually between 2010 and 2015 and treatment coverage increased by 0·23 pp, whereas spending increased by $414 annually between 2015 and 2019 (during which efficiency increased more rapidly) but treatment coverage increased by 0·77 pp.

Of the 25 independent variables investigated, ten were significantly associated with average efficiency in both DEA and SFA regression analyses (table 2). Descriptive graphs for consistently significant variables against efficiency scores are included in the appendix (pp 40–41) Current health expenditure as a percentage of GDP and UHC service coverage index are positively associated with average efficiency. Efficiency is expected to increase as UHC service coverage index or current health expenditure as a proportion of GDP increase. Conversely, out-of-pocket spending as a proportion of current health expenditure, proportion of the population living in slums, average of six World Governance Indicators, and tuberculosis incidence per 100 000 people are negatively associated with average efficiency. Although the typical number of health facility visits after diagnosis of multidrug-resistant tuberculosis, the estimated average duration of hospital stay for drug-susceptible tuberculosis if hospitalised, prevalence of undernourishment, and rural population as a proportion of total population are significantly associated with efficiency in DEA and SFA models, their association is unclear because it is both positive and negative depending on the model. Some variables (eg, tuberculosis spending accounted for by external sources, HIV prevalence, GINI index, and diabetes prevalence) are significantly associated with efficiency in only one of the regression analyses. Of the six SDG indicators associated with tuberculosis incidence considered, two are not significant in either analysis (alcohol use disorders and proportion of the population living below the poverty line).

**Table 2:** Independent variables (n=25) associated with efficiency scores

	**Observed coefficient**	**Standard error**	**p value**	**95% CI**
**Main DEA model regression results**				
Typical number of visits to a health facility after diagnosis for drug-sensitive tuberculosis treatment	0·0001	0·0001	0·4410	−0·0001 to 0·0002
Typical number of visits to a health facility after diagnosis for multidrug-resistant tuberculosis treatment	−4·53 × 10^−5^*	1·86 × 10^−5^	0·0150	−0·0001 to −8·68 × 10^−6^
Estimated proportion of drug-sensitive tuberculosis cases who are hospitalised	−0·0816†	0·0182	<0·0001	−0·1163 to −0·0441
Estimated proportion of multidrug-resistant tuberculosis cases who are hospitalised	0·0321‡	0·0108	0·0030	0·0105 to 0·0529
Estimated average duration of stay for drug-sensitive tuberculosis cases if they are hospitalised	0·0008†	0·0002	0·0010	0·0003 to 0·0013
Estimated average duration of stay for multidrug-resistant tuberculosis cases if they are hospitalised	4·06 × 10^−5^	4·96 × 10^−5^	0·4120	−0·0001 to 0·0001
Governance indicators§	−0·0217*	0·0103	0·0350	−0·0418 to −0·0011
Current health expenditure per capita, PPP (current international $)	−2·09 × 10^−5^	1·70 × 10^−5^	0·2190	−0·0001 to 1·42 × 10^−5^
Current health expenditure (proportion of GDP)	0·7154†	0·1779	<0·0001	0·3673 to 1·0671
External health expenditure (proportion of current health expenditure)	−0·0087	0·0399	0·8270	−0·0856 to 0·0709
Out-of-pocket expenditure (proportion of current health expenditure)	−0·1677†	0·0279	<0·0001	−0·2213 to −0·1124
Population total (log)	0·0035	0·0028	0·2070	−0·0017 to 0·0089
Population per km^2^	−3·20 × 10^−5^	2·04 × 10^−5^	0·1170	−0·0001 to 8·92 × 10^−6^
Rural population (proportion of total population)	0·0664*	0·0259	0·0100	0·0148 to 0·1159
Tuberculosis spending accounted for by external sources (proportion of total tuberculosis spending, excluding private spending)	−0·0657†	0·0166	<0·0001	−0·0995 to −0·0335
UHC service coverage index	0·398†	0·0584	<0·0001	0·2816 to 0·5113
Incidence of tuberculosis per 100 000 people	−0·0003†	0·0000	<0·0001	−0·0004 to −0·0003
Incidence of multidrug-resistant tuberculosis per 100 000 people	0·0019‡	0·0007	0·0040	0·0006 to 0·0032
HIV prevalence (adults age 15 to 49 years; proportion of the population)	0·0239	0·1342	0·8590	−0·2492 to 0·2862
Population living in slums (proportion of urban population)	−0·1075†	0·0283	<0·0001	−0·1618 to −0·0517
Diabetes prevalence (proportion of population age ≥18 years)	0·7799†	0·1169	<0·0001	0·5552 to 1·0119
Alcohol use disorders, 12-month prevalence (proportion of population age ≥15 years)	−0·0739	0·1495	0·6210	−0·3653 to 0·2245
Gini index¶	0·0478	0·0652	0·4630	−0·0810 to 0·1743
Proportion of population living below the international poverty line	−0·0462	0·0350	0·1870	−0·1138 to 0·0230
Prevalence of undernourishment (proportion of population)	0·1851†	0·0483	<0·0001	0·0898 to 0·2784
**Main SFA model regression results**				
Typical number of visits to a health facility after diagnosis for drug-sensitive tuberculosis treatment	−1·65 × 10^−5^	2·50 × 10^−5^	0·51	−6·54 × 10^−5^ to 3·24 × 10^−5^
Typical number of visits to a health facility after diagnosis for multidrug-resistant tuberculosis treatment	2·04 × 10^−5^‡	7·24 × 10^−6^	0·0053	6·22 × 10^−6^ to 3·46 × 10^−5^
Estimated proportion of drug-sensitive tuberculosis cases who are hospitalised	0·0006	0·0070	0·94	−0·013 to 0·014
Estimated proportion of multidrug-resistant tuberculosis cases who are hospitalised	0·0061	0·0035	0·078	−0·0007 to 0·013
Estimated average duration of stay for drug-sensitive tuberculosis cases if they are hospitalised	−1·79 × 10^−4^‡	6·07 × 10^−5^	0·0032	−2·98 × 10^−4^ to −6·04 × 10^−5^
Estimated average duration of stay for multidrug-resistant tuberculosis cases if they are hospitalised	6·79 × 10^−6^	1·24 × 10^−5^	0·58	−1·75 × 10^−5^ to 3·11 × 10^−5^
Governance indicators§	−0·016‡	0·0050	0·0010	−0·026 to −0·0064
Current health expenditure per capita, PPP (current international $)	2·17 × 10^−5^‡	8·16 × 10^−6^	0·0084	5·70 × 10^−6^ to 3·77 × 10^−5^
Current health expenditure (proportion of GDP)	0·19†	0·060	0·0013	0·073 to 0·31
External health expenditure (proportion of current health expenditure)	0·0012	0·017	0·94	−0·032 to 0·035
Out-of-pocket expenditure (proportion of current health expenditure)	−0·037‡	0·012	0·0020	−0·060 to −0·013
Population total (log)	−0·0069†	0·0012	<0·0001	−0·0093 to −0·0045
Population per km^2^	3·17 × 10^−5^†	8·85 × 10^−6^	<0·0001	1·43 × 10^−5^ to 4·90 × 10^−5^
Rural population (proportion of total population)	−0·030*	0·013	0·023	−0·057 to −0·0043
Tuberculosis spending accounted for by external sources (proportion of total tuberculosis spending, excluding private spending)	−0·0050	0·0050	0·31	−0·015 to 0·0047
UHC service coverage index	0·10†	0·014	<0·0001	0·075 to 0·13
Incidence of tuberculosis per 100 000 people	−4·22 × 10^−5^†	1·16 × 10^−5^	<0·0001	−6·49 × 10^−5^ to −1·95 × 10^−5^
Incidence of multidrug-resistant tuberculosis per 100 000 people	−1·70 × 10^−4^	2·11 × 10^−4^	0·42	−5·84 × 10^−4^ to 2·44 × 10^−4^
HIV prevalence (adults age 15 to 49 years; proportion of the population)	−0·107*	0·047	0·024	−0·20 to −0·014
Population living in slums (proportion of urban population)	−0·046‡	0·016	0·0042	−0·077 to −0·014
Diabetes prevalence (proportion of population age ≥18 years)	0·046	0·073	0·53	−0·097 to 0·19
Alcohol use disorders, 12-month prevalence (proportion of population age ≥15 years)	−0·026	0·071	0·71	−0·17 to 0·11
Gini index¶	0·11†	0·025	<0·0001	0·056 to 0·15
Proportion of population living below the international poverty line	0·021	0·015	0·16	−0·0079 to 0·049
Prevalence of undernourishment (proportion of population)	−0·056*	0·026	0·033	−0·11 to −0·0044

Data are based on 1209 observations. SFA coefficients have been multiplied by −1 to show the association with efficiency, rather than inefficiency. DEA=data envelopment analysis. GDP=gross domestic product. PPP=purchasing power parity. SFA=stochastic frontier analysis. UHC=universal health coverage.

* p<0·05.

† p<0·001.

‡ p<0·01.

§ The average of six World Governance Indicators: (1) voice and accountability, (2) political stability and absence of violence or terrorism, (3) government effectiveness, (4) regulatory quality, (5) rule of law, and (6) control of corruption.

¶ In which a score of 0 equates to perfect equality and 100 equates to perfect inequality.

The 13 alternative models we tested in the sensitivity analysis generated variation in the estimated average efficiency compared with the main DEA model (ie, model 1; appendix p 37). The largest variation observed in average efficiency results was with the main SFA analysis (model 2), which was 11·8 pp greater than the main DEA model (except for the constant returns to scale DEA model results, which were generated solely to estimate scale efficiency). Average efficiency varies less than the main DEA model when using the SFA models based on the works of Battese and Coelli (model 14, increase of 6·01 pp) and Pitt and Lee (model 15, increase of 5·05 pp)than in the main DEA model (appendix p 37). Similar variations in average efficiency were seen compared with the main DEA result when only considering drug-susceptible tuberculosis spending, outputs and related independent variables (resulting in a decrease of 6·54 pp [model 7]) or when excluding top and bottom 5% outliers (an increase of 6·52 pp [model 5]). The remaining eight models resulted in smaller differences in average efficiency relative to the main DEA model, between 0·003 pp and 4·55 pp. Subgroup DEA results are similar to main model results (appendix pp 42–45).

Country-level efficiency scores (DEA main model *vs* constant returns to scale [ρ=0·8362; p<0·0001], DEA main model *vs* 3-year time lag [ρ=0·9743; p<0·0001], and DEA main model *vs* 5-year time lag [ρ=0·965; p<0·0001]) and rankings are strongly and significantly correlated across DEA models. However, there is variation in the country-level results between some models, especially between main DEA and SFA efficiency estimates (ρ=0·6584, p<0·0001; appendix p 7) and rankings (ρ=0·5799, p<0·0001; appendix p 10).

## Discussion

To our knowledge, this is the first comprehensive global analysis of the overall efficiency of tuberculosis spending. From our assessment, we found that estimated global tuberculosis treatment coverage can potentially increase from current levels for the same amount of spending. Despite improvements in efficiency, mainly since 2015, large differences remain between countries and only between five and nine of the 30 high tuberculosis burden countries are more efficient than the general average, depending on the analytical method used. Our results indicate increased treatment coverage would improve efficiency, even if this entails additional spending. Government commitment to health, based on the proxy variable of current health expenditure as a proportion of GDP, and progress towards key SDG indicators such as UHC are consistently associated with increased tuberculosis spending efficiency. Our findings have implications for progress towards End TB targets and provide a benchmark to facilitate cross-country learning. They also suggest that efforts to address tuberculosis are complimented by progress towards SDG indicators and wider commitments to improving health-care access that is free at the point of use.

Levels of tuberculosis spending inefficiency estimated in this study are in line with those of the 2010 WHO World Health Report,^29^ which indicated inefficiencies amount to 20–40% of all health spending. We estimated that, for the same amount of spending in 2019, global tuberculosis treatment coverage could have been increased from existing levels by between 12·3% and 26·2%. However, estimated efficiency varied substantially between countries, ranging between 26·0% and 96·7% when estimated using the DEA and 27·8% and 99·9% when estimated using the SFA. Despite differences in methods, these wide variations in efficiency between countries support findings from a previous analysis of tuberculosis spending efficiency in 19 countries between 2011 and 2017 (23·2–79·2%),^9^ another study of national HIV spending in 68 low-income and middle-income countries between 2004 and 2007 (0·59–100%),^16^ and a cross-sectional efficiency analysis of health spending for UHC in 172 countries in 2015 (50·0–100%).^17^

Differences in efficiency between countries are driven predominantly by differences in tuberculosis treatment coverage rather than spending. We found that tuberculosis spending is weakly correlated with DEA and SFA efficiency estimates compared with tuberculosis treatment coverage, which is strongly correlated with both DEA and SFA efficiency estimates. Therefore, to improve efficiency, countries should focus on improving tuberculosis treatment coverage, even if this entails additional spending. This is highlighted by the most efficient countries identified, which spend substantially more on average than the least efficient countries but in turn attain much higher levels of treatment coverage. The likely increase in global efficiency over time, since around 2015, calculated using both DEA and SFA, is also due to increased treatment coverage.

Average efficiency and trends across years, World Bank income groups, and WHO regions were similar between models. Country-level efficiency estimates and rankings were largely consistent across DEA models investigated. However, country-level results should be interpreted with caution because there is variation between models, in particular the main DEA and SFA country efficiency estimates and rankings. Nonetheless, some countries such as Brazil, Russia, Paraguay, Venezuela, Lebanon, Iran, and Rwanda are consistently among the most efficient across analyses. Others were consistently among the least efficient such as Nigeria, Mongolia, Indonesia, Ghana, and Laos.

Our findings have some similarities with previous efficiency studies. Brazil was found to be the second most efficient country among 19 countries in a previous analysis of tuberculosis spending efficiency, while Nigeria and Indonesia were the least efficient.^9^ Additionally, Rwanda, Paraguay, and Brazil were among the most efficient countries in a study of HIV spending efficiency.^16^ Rwanda was also among the most efficient countries in two recent efficiency studies of health spending for UHC in 172 countries and 204 countries.^17^, ^24^ Therefore, although individual country results should be interpreted with caution, the tuberculosis responses of countries consistently identified as most efficient in this study can be a useful benchmark for similar counterparts to draw lessons from.

Our estimates might be especially useful for the 30 high tuberculosis burden countries, 21 (70%) to 25 (83%) of which are less efficient than the general average, depending on the analytical method used. Potential reasons for high efficiency in Rwanda, for example, could be applicable to similar contexts. Having met Millennium Development Goals, Rwanda has substantially reduced tuberculosis deaths and provided national coverage of community tuberculosis care, in addition to reductions in mortality due to HIV/AIDS and malaria, and maternal and under-5 mortality.^30^, ^31^, ^32^ This progress has been accompanied by the use of evidence-informed equitable policies, like rapid expansion of health insurance focused on the poor and vulnerable, integration and quality improvement of health services, progress towards UHC, strengthening of the health system and workforce, and wider efforts at poverty reduction.^30^, ^32^, ^33^

Although some efficiency gains will be in the control of a country's national tuberculosis programme, our regression analysis results indicate that some differences in efficiency are probably due to wider contextual factors. Commitment to health is associated with increased tuberculosis spending efficiency, as shown by consistent significant positive associations with current health expenditure as a proportion of GDP and the UHC service coverage index. This finding supports the study of HIV spending efficiency, for which a significant positive association has been found between efficiency and the variable “government commitment to health”.^16^ By contrast, efficiency decreases as out-of-pocket spending as a proportion of current health expenditure increases, which supports calls to reduce out-of-pocket spending in health and suggests this could be associated with greater efficiency and not just added financial risk protection. Considered in combination, current health expenditure as a proportion of GDP, UHC service coverage index, and out-of-pocket spending as a proportion of current health expenditure indicate that governments comprehensively pursuing progress towards UHC might also improve efficiency in traditionally vertical programmes and disease areas.

More widely, consistently significant negative associations between efficiency and the proportion of urban populations living in slums suggest that governments pursuing multisectoral development and poverty reduction strategies have also increased tuberculosis spending efficiency. These findings support existing literature advocating the need for multisectoral approaches and wider social protection to improve on key tuberculosis indicators.^34^, ^35^ However, regression results should not be interpreted as causal associations. We only investigated the presence or absence of an association with efficiency.

Some key limitations must be considered when interpreting our findings. First, we assumed tuberculosis spending and treatment coverage to be comparable across included countries, which, in reality, is not always the case. Second, we only included treatment coverage in our analysis, and no prevention interventions or outcomes. This approach might bias our results towards curative rather than preventive responses. Third, more complete data on SDG indicators are needed. Excluding SDG indicators in a sensitivity analysis did not notably affect results. However, given the amount of missing data for financial risk protection, social protection coverage, and smoking prevalence, we were compelled to exclude these factors from investigation. The association between efficiency and SDG variables considered should be analysed again once more data are available. Fourth, we did not consider quality of care in this study. Results from sensitivity analyses that incorporated success rates in outputs, and therefore indirectly adherence, treatment failure, and mortality, were similar to main model findings. However, these results do not wholly reflect quality of care. Given the substantial negative impact of COVID-19 on key tuberculosis outcomes, such as treatment coverage and mortality,^36^, ^37^ it would be beneficial to repeat our analyses when additional country–year data become available.

Overall, our study is a useful reference to identify countries that are consistently most efficient and emulate relevant aspects of their tuberculosis responses in similar contexts with lower efficiency. Country-level analyses can provide further insight into how the performance of national tuberculosis programmes and associated health providers can be improved. Improved efficiency in some countries, especially high tuberculosis burden countries, might accelerate progress towards End TB targets. However, even in the most efficient countries we analysed, incidence rates have not decreased sufficiently to meet End TB targets.^1^ These results support calls for greater investment in tuberculosis.^1^, ^6^ Insufficient progress towards End TB targets among the most efficient countries and regression analysis results suggest ending tuberculosis will require multisectoral action, including social protection and UHC to ensure all people with tuberculosis have access to affordable quality care without incurring catastrophic health expenditures.^1^, ^34^, ^35^, ^38^, ^39^ Patient-centred and multisectoral approaches were key during 2020 as the COVID-19 emergency response involved rethinking the delivery of tuberculosis care and approaches to find missing cases with ever more constrained resources and the threat of reversing advances as tuberculosis case detection dropped. Such efforts and approaches need to continue and will be key for progress towards End TB targets and to eventually end tuberculosis.

## Data sharing

WHO Global TB Report series data for 2010–19 on tuberculosis spending and the number of visits, proportion of cases hospitalised, tuberculosis spending accounted for by external sources (proportion of total public tuberculosis spending, excluding private spending), and duration of hospitalisation can be made available upon official request from the WHO Global TB Programme at https://www.who.int/teams/global-tuberculosis-programme/about. All other data used for this study were extracted from publicly available sources that are listed in the appendix (pp 2–4).

## Declaration of interests

We declare no competing interests.
