# Supplementary material for: National tuberculosis spending efficiency and its associated factors in 121 low-income and middle-income countries, 2010–19: a data envelopment and stochastic frontier analysis
Source: Lancet Glob Health. 2022 Apr 12;10(5):e649–60. doi: 10.1016/S2214-109X(22)00085-7 (PMC9023749; doi:10.1016/S2214-109X(22)00085-7)
Supplement: Supplementary appendix [file mmc1.pdf]

THE LANCET
Global Health

Supplementary appendix

This appendix formed part of the original submission and has been peer reviewed. We post it as supplied by the authors.

Supplement to: Abou Jaoude GJ, Garcia Baena I, Nguhiu P, et al. National tuberculosis spending efficiency and its associated factors in 121 low-income and middle-income countries, 2010–19: a data envelopment and stochastic frontier analysis. *Lancet Glob Health* 2022; **10:** e649–60.

Table S1. Expected effect of independent variables on overall efficiency and justification for inclusion in the analysis

Table S2. Missing data by year and country for SDG independent variables

Table S3. Multicollinearity test, variance inflation factor (VIF) for included independent variables

Table S4. Full results with DEA (variable and constant returns to scale) scores, bias estimates and bias-corrected efficiency scores, scale efficiency and SFA efficiency scores

Table S5. Sub-regional DEA analysis regression results

Figure S1a. Scatter plots of 1209 bias-corrected efficiency scores against the original DEA efficiency scores

Figure S1b. Scatter plot of 1209 DEA bias-corrected efficiency scores against the main SFA efficiency scores

Figure S2a. Graph of average DEA bias-corrected efficiency score by World Bank income group over time: 2010-2019

Figure S2b. Graph of average SFA efficiency score by World Bank income group over time: 2010-2019

Figure S2c. Graph of average DEA bias-corrected efficiency score by WHO region over time: 2010-2019

Figure S2d. Graph of average SFA efficiency score by WHO region over time: 2010-2019

Figure S3. Graph of country rankings by model, relative to main DEA model results

Figure S4. Sensitivity analysis: average efficiency scores of 14 alternative models compared with the main model

Figure S5a. Graph of average log of input and output by country: 2010-2019

Figure S5b. Graph of average log of input and three outcome-adjusted coverage outputs by country: 2010-2019

Figures S6. Average DEA and SFA efficiency scores by country between 2010-2019

Figures S7a-S7n. Descriptive plots of independent variables against DEA and SFA efficiency scores

Figures S8a-8e. Main versus sub-regional DEA analyses average scores and rankings

Table S1. Expected effect of independent variables on overall efficiency and justification for inclusion in the analysis

Independent variable	Expected effect	Justification	Data source
Typical number of visits to a health facility after diagnosis for DS-TB treatment	-	A higher number of health facility visits indicates prioritising health facility delivery of direct-observed treatment care over community or hospital care, as well as longer treatment durations which are more costly. Longer treatment durations and higher numbers of required health facility visits could also increase loss to follow-up and lower treatment completion rates. Efficiency scores are therefore expected to decrease as the number of visits increases beyond a certain point.	WHO Global TB Report series data [1]
Typical number of visits to a health facility after diagnosis for MDR-TB treatment	-		
Estimated percentage of DS-TB cases that are hospitalised	+ or -	Some DS-TB cases may require hospitalisation given underlying co-morbidities or potential adverse treatment effects. A small percentage of hospitalisation therefore likely indicates good quality care that reduces the likelihood of treatment failure – thus raising efficiency scores. However, excessive hospitalisation could in turn be unnecessary, lead to nosocomial transmission and result in lower efficiency scores due to the higher costs of tertiary care.	WHO Global TB Report series data [1]
Estimated percentage of MDR-TB cases that are hospitalised	+	The majority of MDR-TB cases will need some hospitalisation in initial treatment phase or in the presence of complications. Higher percentages of MDR-TB hospitalisation can indicate good quality care that could identify and minimise adverse effects and monitor initial responses to drug regimens. This reduces the likelihood of treatment failure due to inappropriate drug regimens and thus should raise efficiency scores.	WHO Global TB Report series data [1]
Estimated average duration of stay for DS-TB cases if they are hospitalised	-	It is expected that as the duration of hospitalisation increases, efficiency will decrease given that hospitalisation is more costly than primary care with similar outcomes and it is not recommended in the case of DS-TB cases.	WHO Global TB Report series data [1]
Estimated average duration of stay for MDR cases if they are hospitalised	-	While hospitalisation is often necessary for MDR-TB cases, it is usually only necessary in the initial phase and for a few weeks. It is therefore expected that as the duration of hospitalisation increases, efficiency will decrease given that hospitalisation is more costly than primary care with similar outcomes.	WHO Global TB Report series data [1]
TB spending accounted for by external sources (% of total TB spending, excluding private spending)	+ or -	Development assistance and external funding for TB could improve efficiency with health systems support, such as higher quality and lower drug prices via cheaper procurement lines and technical support for strengthening surveillance and TB response. External funding for TB has played a major role [1] in effectively complementing government budgets. However, given that external TB funding often complements government budgets to address resource gaps, higher levels of external funding also reflect under-resourced health systems with lower overall capacity. Aid dependent countries face challenges: limited predictability of donor funds, disconnect between pledges and disbursement timings, donor conditionalities. Aid dependency also might also provoke fragmentation and limited use of aid modalities for broader government support and pooled sector funding.	WHO Global TB Report series data [1]
Average of six World Governance Indicators	+	Zeng and colleagues included several World Bank governance indicators in their efficiency analysis of HIV spending.[21] Good governance is expected to be associated with improved efficiency.[22] For this analysis, scores for each of the six World Bank governance indicators (Voice and Accountability, Political Stability and Absence of Violence, Government Effectiveness, Regulatory Quality, Rule of Law, and Control of Corruption) were highly correlated and therefore combined to generate an average as done by Jordi and colleagues in their efficiency analysis of spending on UHC.[22]	World Bank [2]
Population total	+	As population increases, economies of scale could improve overall efficiency.	World Bank Open Database [3]
Population per km2	+ or -	More densely populated areas are less costly to reach. A higher population density suggests higher levels of urbanisation and increased likelihood of household access to health facilities. However, higher	World Bank Open Database [3]

		population density may also reflect crowding and an increased probability of TB transmission.	
Rural population (% of total population)	-	Rural areas tend to have a lower density of health workers and fewer health facilities that receive less funding than in urban areas and are further away from catchment populations. These can result in lower treatment coverage and poorer TB care. Efficiency is therefore expected to decrease as rurality increases.	World Bank Open Database [3]
Current health expenditure (% of GDP)	+	The percentage of GDP allocated to health can reflect government commitment to health. Governments that prioritise and focus on health are more likely to achieve high levels of efficiency.	World Bank Open Database [3]
Current health expenditure per capita, PPP (current international $)	+	Higher levels of per capita spending on health indicates better resourced health systems that can adopt more efficient practices with resources available for investments in health infrastructure and technology. CHE per capita was also significantly correlated with Gross National Income (GNI) per capita expressed in international dollars (0·81, p<0·001). A higher GNI per capita suggests higher levels of development and living standards.	World Bank Open Database [3]
External health expenditure (% of CHE)	-	Higher levels of external health spending mean a greater overall dependence of health systems on development assistance, indicating lower capacity to provide services. In addition, external health spending can also compete with existing national priorities and objectives. The latter could lead to fragmentation in the health system and result in higher levels of inefficiency.	World Bank Open Database [3]
Out-of-pocket expenditure (% of CHE)	-	Higher proportions of overall health spending comprised of OOP, indicate underdeveloped health financing structures. The latter are not only associated with financial barriers to accessing care, but also the quality and availability of health services. As the most regressive form of health financing, higher proportions of OOP spending are expected to result in lower efficiency.	World Bank Open Database [3]
UHC service coverage index	+	A higher UHC service coverage index, reflects progress toward UHC through a greater coverage of and access to essential healthcare services at a national level. Efficiency scores are therefore expected to increase as the UHC index increases, given that progress toward UHC will likely be associated with better outcomes of TB care and efficient national planning, implementation and provision of care. The UHC service coverage index was also significantly correlated both with the Human Development Index (ρ=0·85, p<0·001) and Gross National Income per capita in international dollars (ρ=0·69, p<0·001), which were therefore not included as separate independent variables.	WHO Global Health Observatory [4]
TB incidence per 100k population	+ or -	High incidence of TB can be an indicator of poor outcomes, such as low treatment coverage (if the number of missing cases is large), which would result in lower efficiency scores. However, other social and environmental factors such as poverty are significant drivers of TB incidence. Higher TB incidence could allow for economies of scale, thus lowering costs and resulting in higher efficiency scores if programmatic outcomes are not poor. For example, Zeng and colleagues found a significant positive association between HIV prevalence and the efficiency of HIV spending.[21]	WHO Global Health Observatory [4]
MDR-TB incidence per 100k population	-	Efficiency scores are expected to decrease as MDR-TB incidence increases given that higher rates of drug-resistance may be indicators of poor diagnosis and treatment outcomes. In addition, diagnosis and treatment outcomes for MDR-TB tend to be inferior and costlier compared with care for DS-TB.	WHO Global Health Observatory [4]
HIV prevalence (adults 15-49)	-	Higher HIV prevalence increases population susceptibility to TB and therefore the likelihood of high HIV-TB comorbidity. HIV-TB comorbidity requires integrated approaches to the management of care and disease control. Integrated approaches to comorbidity management are often lacking or recently implemented, leading to poorer outcomes, and as such efficiency is expected to decrease as HIV prevalence increases.	Joint United Nations Programme on HIV/AIDS [5]

Population living in slums (% of urban population)	-	These are the “SDG indicators beyond SDG 3 that are associated with TB incidence” included in the WHO TB report 2020.[1] Each of these six indicators affects TB incidence and consequently the denominator of the treatment coverage variable used as an output in the main model. The six SDG indicators (population living in slums, diabetes prevalence, alcohol use disorders, GINI index, population living in poverty and prevalence of undernourishment) are therefore expected to have a negative association with the overall efficiency of TB spending. However, a positive association may be observed with diabetes prevalence or the GINI index as both may increase with GDP and countries with higher GDP may have higher levels of efficiency.	UN SDG database [6]
Diabetes prevalence (% of population aged >= 18 years)	+ or -		WHO Global Health Observatory [4]
Alcohol use disorders, 12-month prevalence (% of population aged >= 15 years)	-		WHO Global Health Observatory [4]
GINI index (0=perfect equality, 100=perfect inequality)	+ or -		World Bank Open Database [3]
Population living below the international poverty line (% of population)	-		UN SDG database [6]
Prevalence of undernourishment (% of population)	-		World Bank Open Database [3]

Note: GDP: Gross Domestic Product, CHE: Current Health Expenditure, UHC: Universal Health Coverage

[1] World Health Organisation. WHO estimates published in Global TB Report series. World Health Organisation; 2020.
[2] World Bank. Worldwide Governance Indicators [Internet]. The World Bank. 2020 [Accessed: 20 October 2020]. Available from: https://info.worldbank.org/governance/wgi/.
[3] World Bank. World Bank Open Data [Internet]. World Bank. 2020 [Accessed: 20 October 2020]. Available from: https://data.worldbank.org/.
[4] World Health Organisation. Global Health Observatory Data [Internet]. World Health Organisation. 2020 [Accessed: 20 October 2020]. Available from: apps.who.int/gho/data/.
[5] UNAIDS. AIDSinfo [Internet]. UNAIDS. 2020 [Accessed: 20 October 2020]. Available from: https://aidsinfo.unaids.org/
[6] United Nations. Global SDG Indicators Database [Internet]. United Nations. 2020 [Accessed: 20 October 2020]. Available from: https://unstats.un.org/sdgs/indicators/database/.

Table S2. Missing data by year and country for SDG independent variables

Variable	Country	Year	Imputation method	Included in final model	Reason for exclusion
Access to clean fuels and technologies for cooking (% of population)	2·48%	31·8%	Ordinary least squares	No	Highly correlated with UHC index (0·819, p<0·001)
Population living in slums (% of urban population)	17·4%	80·1%	Ordinary least squares	Yes	n/a
Smoking prevalence (% of population aged >= 15 years)	28·9%	64·4%	n/a	No	Data missing for too many countries
Diabetes prevalence (% of population aged >= 18 years)	1·65%	50·7%	Ordinary least squares	Yes	n/a
Population covered by social protection floors/systems (% of population)	39·7%	87·3%	n/a	No	Data missing for too many countries
Alcohol use disorders, 12 month prevalence (% of population aged >= 15 years)	2·5%	90·2%	Assumed fixed over time (i.e. applied 2016 values to all years)	Yes	n/a
GINI index (0=perfect equality, 100=perfect inequality)	17·4%	72·0%	Ordinary least squares	Yes	n/a
Population living below the international poverty line (% of population)	17·4%	72·0%	Ordinary least squares	Yes	n/a
Prevalence of undernourishment (% of population)	14·9%	32·0%	Ordinary least squares	Yes	n/a
UHC index of essential service coverage (based on 14 tracer indicators including TB treatment)	3·31%	80·8%	Assumed fixed over time (i.e. applied 2015 and 2017 values to years before and after, along with average in between)	Yes	n/a
Greater than 10% of total household expenditure or income on health (% of population)	41·4%	85·8%	n/a	No	Data missing for too many countries
Greater than 25% of total household expenditure or income on health (% of population)	41·4%	85·8%	n/a	No	Data missing for too many countries

Table S3. Multicollinearity test, variance inflation factor (VIF) for included independent variables

Variable (n=25)	VIF	SQRT VIF	Tolerance	R-Squared
Typical number of visits to a health facility after diagnosis for DS-TB treatment	1·57	1·25	0·64	0·36
Typical number of visits to a health facility after diagnosis for MDR-TB treatment	1·56	1·25	0·64	0·36
Estimated percentage of DS-TB cases that are hospitalised	2·47	1·57	0·40	0·60
Estimated percentage of MDR-TB cases that are hospitalised	1·49	1·22	0·67	0·33
Estimated average duration of stay for DS-TB cases if they are hospitalised	2·32	1·52	0·43	0·57
Estimated average duration of stay for MDR cases if they are hospitalised	1·77	1·33	0·56	0·44
Governance indicator	2·73	1·65	0·37	0·63
Current health expenditure per capita, PPP (current international $)	3·84	1·96	0·26	0·74
Current health expenditure (% of GDP)	1·90	1·38	0·53	0·47
External health expenditure (% of CHE)	3·35	1·83	0·30	0·70
Out-of-pocket expenditure (% of CHE)	2·47	1·57	0·40	0·60
Population total (log)	2·47	1·57	0·40	0·60
Population per km2	1·19	1·09	0·84	0·16
Rural population (% of total population)	2·14	1·46	0·47	0·53
TB spending accounted for by external sources (% of total TB spending, excluding private spending)	2·02	1·42	0·49	0·51
TB incidence per 100k	2·19	1·48	0·46	0·54
MDR-TB incidence per 100k	1·98	1·41	0·50	0·50
UHC service coverage index	5·23	2·29	0·19	0·81
Population living in slums (% of urban population)	3·11	1·76	0·32	0·68
Diabetes prevalence (% of population aged >= 18 years)	2·11	1·45	0·47	0·53
Alcohol use disorders, 12 month prevalence (% of population aged >= 15 years)	1·63	1·27	0·62	0·38
GINI index (0=perfect equality, 100=perfect inequality)	1·98	1·41	0·51	0·49
Population living below the international poverty line (% of population)	4·54	2·13	0·22	0·78
Prevalence of undernourishment (% of population)	2·71	1·65	0·37	0·63
HIV prevalence (adults 15-49)	3·09	1·76	0·32	0·68
**Mean VIF**	**2·48**			

Figure S1a. Scatter plot of 1209 bias-corrected efficiency scores against the original DEA efficiency scores

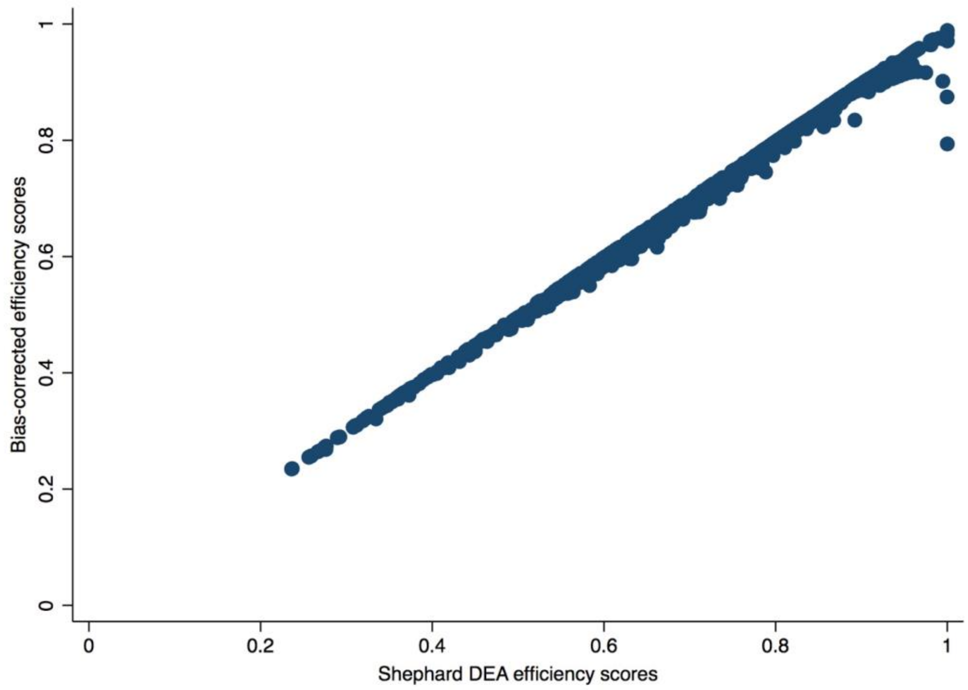

The correlation coefficient between the original Shepard DEA efficiency scores and the bias-corrected efficiency scores is 0·998 ($p<0·001$) for 1209 observations.

Figure S1b. Scatter plot of 1209 DEA bias-corrected efficiency scores against the main SFA efficiency scores

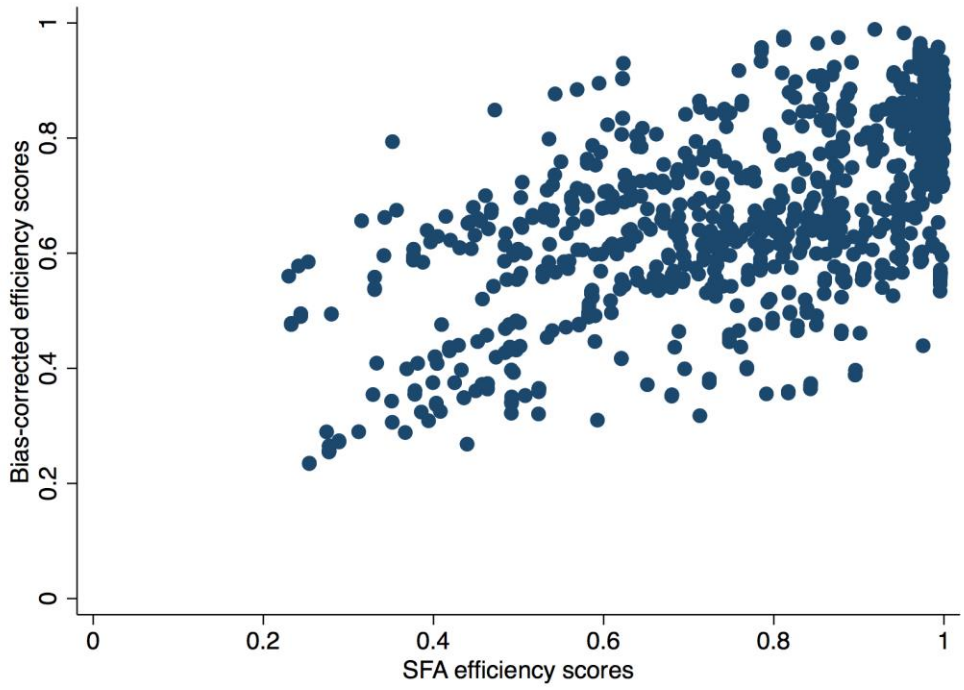

The correlation coefficient between the DEA bias-corrected efficiency scores and main SFA efficiency scores is 0·6584 ($p<0·001$) for 1209 observations.

Figure S2a. Graph of average DEA bias-corrected efficiency score by World Bank income group over time: 2010-2019

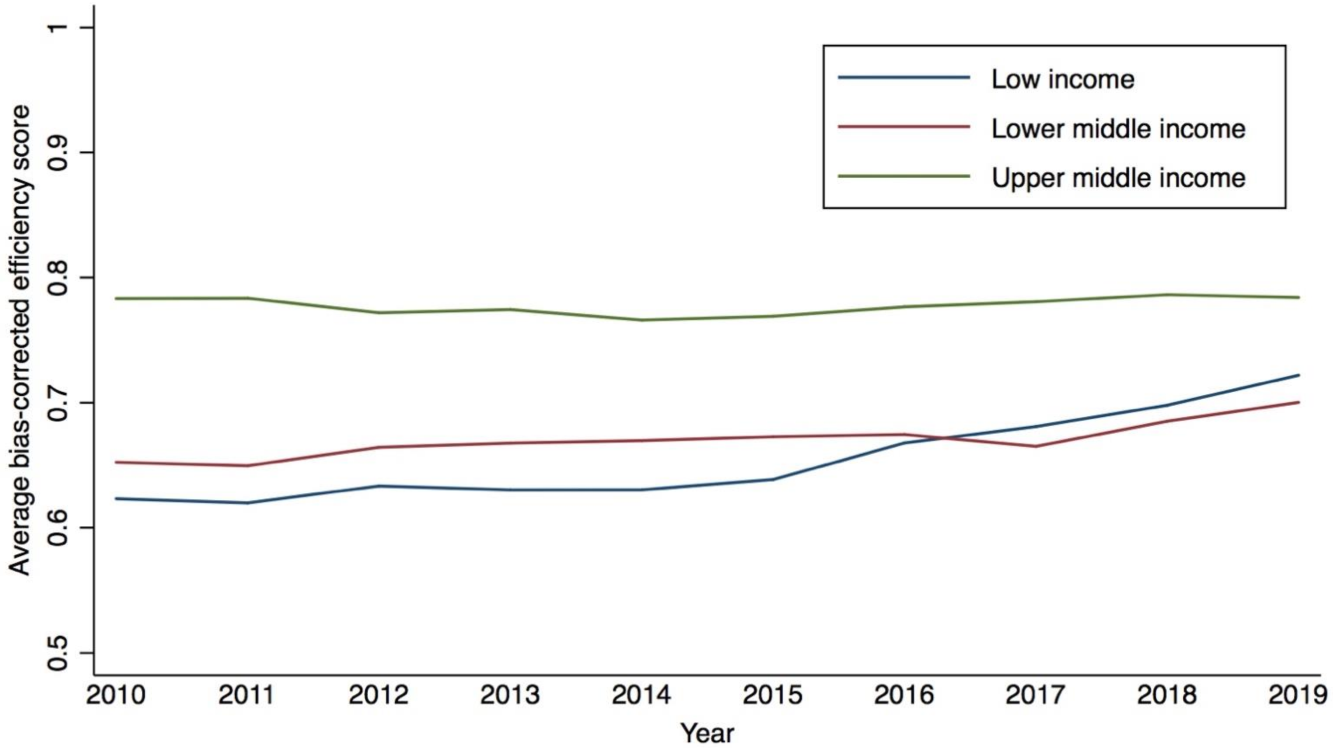

Figure S2b. Graph of average SFA efficiency score by World Bank income group over time: 2010-2019

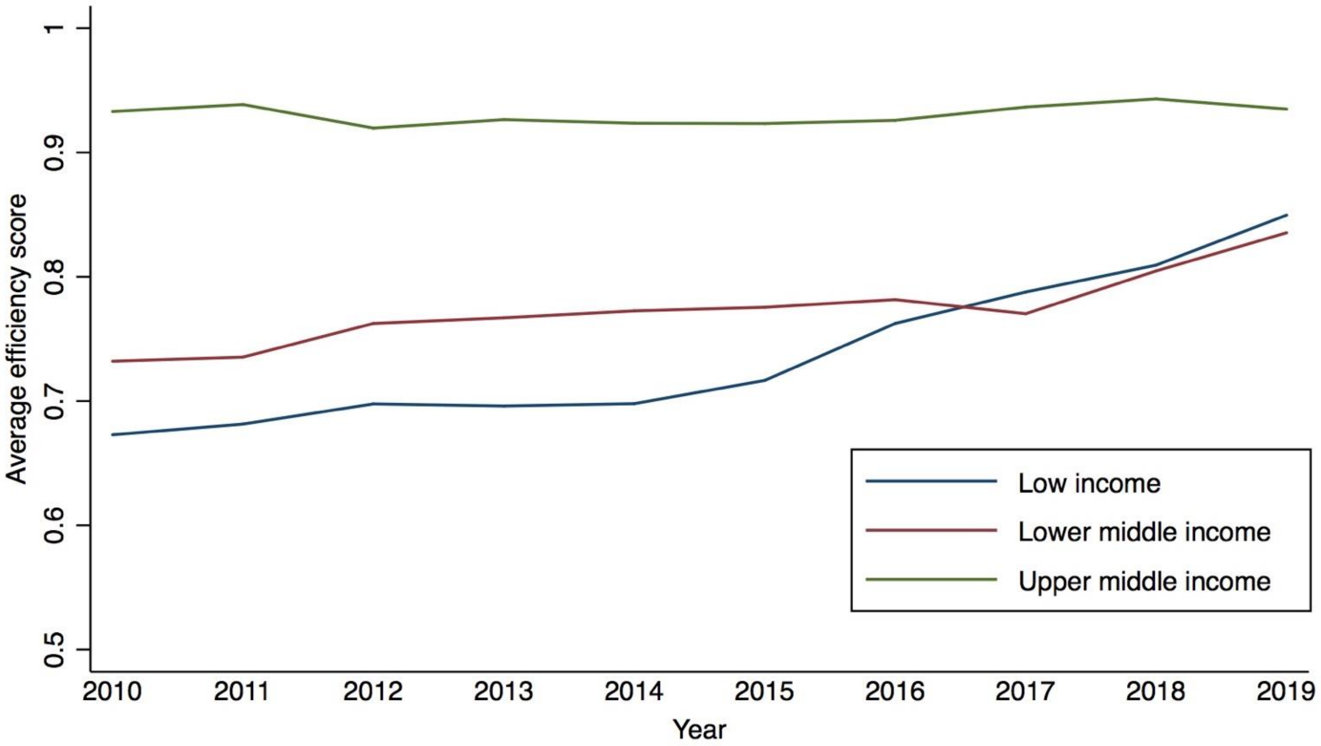

Figure S2c. Graph of average DEA bias-corrected efficiency score by WHO region over time: 2010-2019

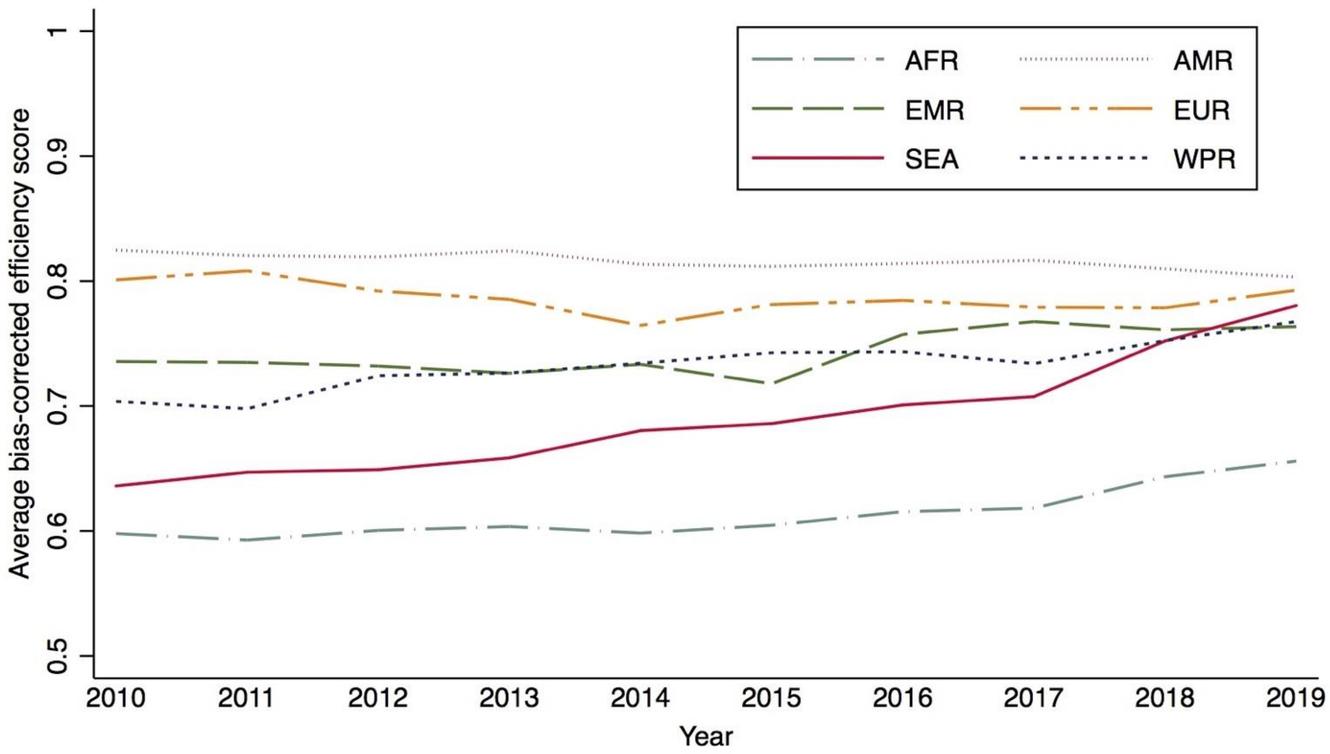

Figure S2d. Graph of average SFA efficiency score by WHO region over time: 2010-2019

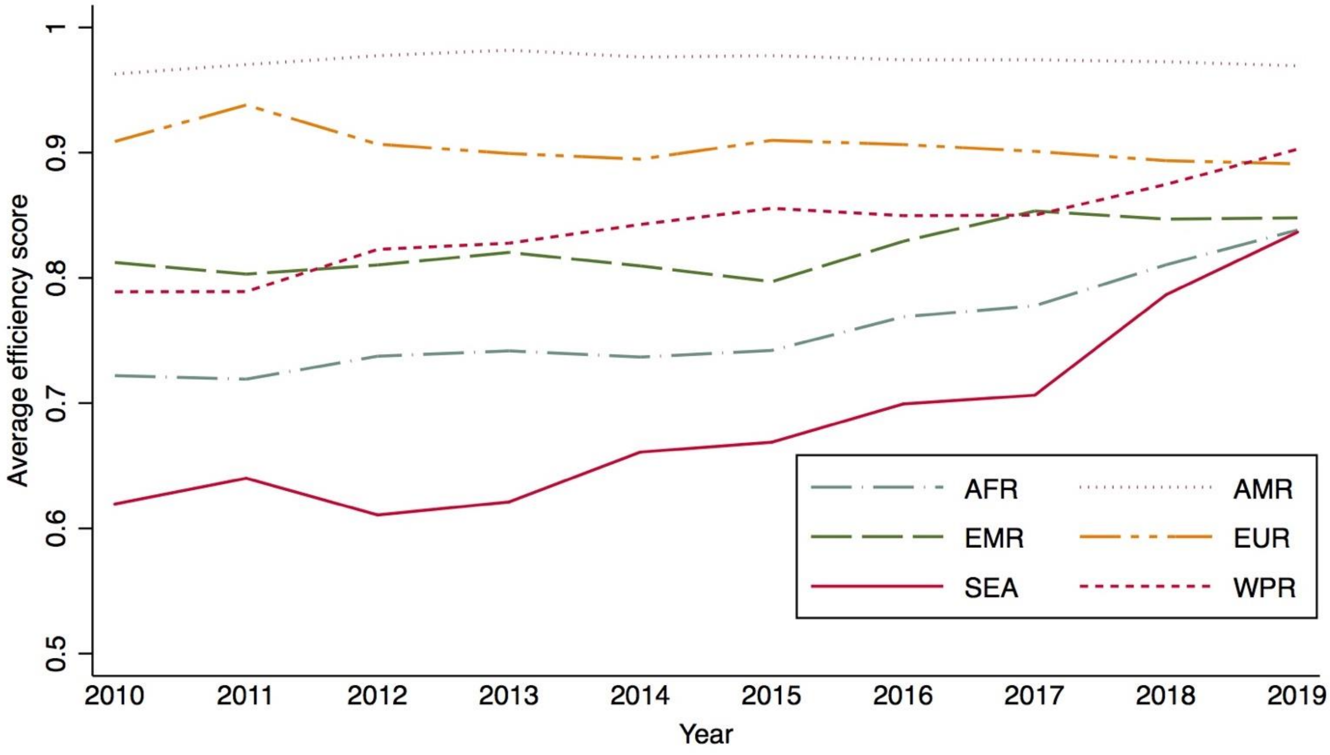

Figure S3. Graph of country rankings by model, relative to main DEA model results

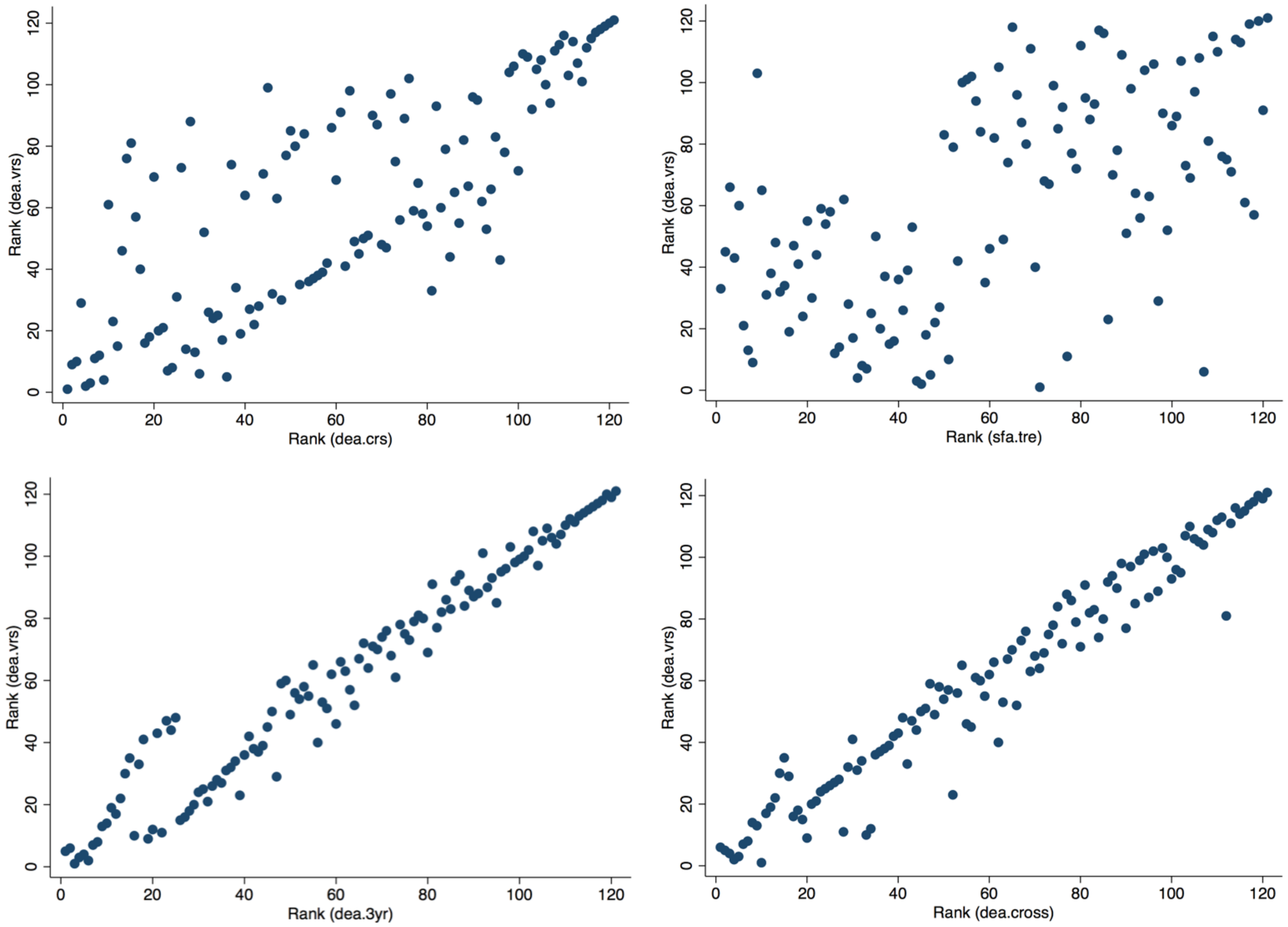

Note: dea.vrs = main DEA model, sfa.tre = SFA model, dea.3yr = 3-year lagged investment DEA model, dea.cross = cross-sectional DEA model using 10 year average for all variables.

Table S4. Full results with DEA (variable and constant returns to scale) scores, bias estimates and bias-corrected efficiency scores, scale efficiency and SFA efficiency scores

Country	iso3	Year	WHO Region	WB Income Group	DEA Score (VRS)	Bias estimate (VRS)	Bias-corrected DEA score (VRS)	SFA efficiency score	DEA Score (CRS)	Bias estimate (CRS)	Bias-corrected DEA score (CRS)	Scale efficiency (CRS/ VRS)
Afghanistan	AFG	2010	EMR	LIC	57.55	1.03	56.52	50.27	49.67	3.21	46.46	82.20
Afghanistan	AFG	2011	EMR	LIC	55.46	1.23	54.23	47.05	48.52	3.14	45.39	83.70
Afghanistan	AFG	2012	EMR	LIC	55.35	1.07	54.28	47.05	47.97	3.10	44.87	82.66
Afghanistan	AFG	2013	EMR	LIC	56.26	0.83	55.43	48.65	47.98	3.10	44.87	80.95
Afghanistan	AFG	2014	EMR	LIC	56.14	0.74	55.40	48.64	47.48	3.07	44.41	80.16
Afghanistan	AFG	2015	EMR	LIC	61.17	0.57	60.60	56.95	50.30	3.25	47.05	77.65
Afghanistan	AFG	2016	EMR	LIC	70.21	0.69	69.52	71.31	58.04	3.75	54.29	78.09
Afghanistan	AFG	2017	EMR	LIC	76.65	1.27	75.38	80.94	65.82	4.25	61.57	81.68
Afghanistan	AFG	2018	EMR	LIC	77.86	1.40	76.47	82.92	67.19	4.34	62.85	82.19
Afghanistan	AFG	2019	EMR	LIC	82.17	1.23	80.94	91.02	70.16	4.53	65.62	81.08
Angola	AGO	2010	AFR	LMIC	55.89	0.60	55.29	64.02	46.59	3.01	43.58	78.83
Angola	AGO	2011	AFR	LMIC	56.38	0.49	55.89	65.92	45.73	2.96	42.77	76.53
Angola	AGO	2012	AFR	LMIC	59.98	0.55	59.43	71.78	49.16	3.18	45.98	77.37
Angola	AGO	2013	AFR	LMIC	67.25	0.82	66.43	83.98	56.56	3.66	52.91	79.64
Angola	AGO	2014	AFR	LMIC	59.36	0.53	58.82	71.77	47.62	3.08	44.54	75.72
Angola	AGO	2015	AFR	LMIC	65.06	0.82	64.24	79.85	54.85	3.55	51.31	79.87
Angola	AGO	2016	AFR	LMIC	62.51	0.58	61.93	77.78	49.96	3.23	46.73	75.46
Angola	AGO	2017	AFR	LMIC	55.24	0.49	54.75	65.90	42.83	2.77	40.07	73.18
Angola	AGO	2018	AFR	LMIC	66.18	0.65	65.54	86.03	51.73	3.34	48.39	73.83
Angola	AGO	2019	AFR	LMIC	74.12	0.99	73.13	96.78	62.74	4.06	58.68	80.25
Argentina	ARG	2010	AMR	UMIC	86.36	0.28	86.07	99.09	59.89	3.87	56.02	65.09
Argentina	ARG	2011	AMR	UMIC	85.23	0.32	84.91	99.07	59.06	3.82	55.25	65.06
Argentina	ARG	2012	AMR	UMIC	84.82	0.34	84.49	99.07	58.79	3.80	54.99	65.09
Argentina	ARG	2013	AMR	UMIC	88.16	0.26	87.90	99.12	61.46	3.97	57.49	65.40
Argentina	ARG	2014	AMR	UMIC	82.10	0.54	81.56	99.05	57.17	3.70	53.48	65.57
Argentina	ARG	2015	AMR	UMIC	79.82	1.08	78.73	99.06	56.05	3.62	52.43	66.59
Argentina	ARG	2016	AMR	UMIC	83.90	0.39	83.51	99.04	58.20	3.76	54.44	65.18
Argentina	ARG	2017	AMR	UMIC	82.25	0.52	81.72	99.06	57.25	3.70	53.55	65.53
Argentina	ARG	2018	AMR	UMIC	88.72	0.26	88.45	99.13	62.02	4.01	58.01	65.59
Argentina	ARG	2019	AMR	UMIC	90.51	0.30	90.21	99.14	64.20	4.15	60.05	66.57
Armenia	ARM	2010	EUR	UMIC	74.95	0.58	74.37	98.75	52.29	3.38	48.91	65.77
Armenia	ARM	2011	EUR	UMIC	73.33	1.02	72.31	98.74	51.51	3.33	48.18	66.63
Armenia	ARM	2012	EUR	UMIC	74.71	0.63	74.08	98.75	52.17	3.37	48.79	65.87
Armenia	ARM	2013	EUR	UMIC	73.61	0.92	72.69	98.74	51.64	3.34	48.30	66.45
Armenia	ARM	2014	EUR	UMIC	72.73	0.52	72.21	98.69	49.17	3.18	45.99	63.69
Armenia	ARM	2015	EUR	UMIC	72.73	0.53	72.20	98.70	49.26	3.18	46.08	63.82
Armenia	ARM	2016	EUR	UMIC	72.73	0.66	72.06	98.71	49.99	3.23	46.76	64.88
Armenia	ARM	2017	EUR	UMIC	72.73	0.81	71.92	98.72	50.49	3.26	47.22	65.66
Armenia	ARM	2018	EUR	UMIC	72.73	0.97	71.76	98.73	50.91	3.29	47.62	66.36
Armenia	ARM	2019	EUR	UMIC	83.38	0.29	83.10	98.87	59.26	3.83	55.43	66.71
American Samoa	ASM	2010	WPR	UMIC	79.09	0.39	78.70	99.81	50.61	3.27	47.34	60.15
American Samoa	ASM	2011	WPR	UMIC	79.09	0.33	78.77	99.75	47.46	3.07	44.39	56.36

American Samoa	ASM	2012	WPR	UMIC	79.09	0.33	78.77	99.75	47.50	3.07	44.43	56.41
American Samoa	ASM	2013	WPR	UMIC	79.09	0.33	78.76	99.76	47.57	3.07	44.49	56.49
American Samoa	ASM	2014	WPR	UMIC	79.09	0.33	78.76	99.76	47.67	3.08	44.59	56.62
American Samoa	ASM	2015	WPR	UMIC	79.09	0.40	78.69	99.82	51.24	3.31	47.93	60.91
American Samoa	ASM	2016	WPR	UMIC	79.09	0.40	78.69	99.82	51.24	3.31	47.93	60.91
American Samoa	ASM	2017	WPR	UMIC	79.28	1.30	77.98	99.87	55.81	3.61	52.20	66.94
American Samoa	ASM	2018	WPR	UMIC	79.09	0.31	78.78	99.58	41.18	2.66	38.52	48.90
American Samoa	ASM	2019	WPR	UMIC	79.09	0.31	78.78	99.68	44.55	2.88	41.67	52.89
Azerbaijan	AZE	2010	EUR	UMIC	81.78	0.24	81.54	97.94	57.25	3.70	53.55	65.67
Azerbaijan	AZE	2011	EUR	UMIC	82.42	0.25	82.17	97.95	57.98	3.75	54.24	66.00
Azerbaijan	AZE	2012	EUR	UMIC	80.88	0.24	80.64	97.93	56.34	3.64	52.70	65.35
Azerbaijan	AZE	2013	EUR	UMIC	81.79	0.24	81.55	97.94	57.26	3.70	53.56	65.68
Azerbaijan	AZE	2014	EUR	UMIC	74.30	0.72	73.58	97.86	51.97	3.36	48.61	66.06
Azerbaijan	AZE	2015	EUR	UMIC	80.01	0.25	79.76	97.91	55.56	3.59	51.97	65.16
Azerbaijan	AZE	2016	EUR	UMIC	82.27	0.25	82.02	97.95	57.80	3.74	54.06	65.92
Azerbaijan	AZE	2017	EUR	UMIC	81.84	0.24	81.59	97.94	57.31	3.70	53.60	65.70
Azerbaijan	AZE	2018	EUR	UMIC	77.19	0.35	76.83	97.88	53.54	3.46	50.08	65.18
Azerbaijan	AZE	2019	EUR	UMIC	80.84	0.24	80.60	97.93	56.30	3.64	52.66	65.34
Burundi	BDI	2010	AFR	LIC	69.80	1.99	67.81	94.50	62.90	4.07	58.84	86.77
Burundi	BDI	2011	AFR	LIC	63.31	2.07	61.24	79.26	59.22	3.83	55.39	90.46
Burundi	BDI	2012	AFR	LIC	62.23	0.57	61.66	83.47	51.07	3.30	47.76	77.47
Burundi	BDI	2013	AFR	LIC	68.05	0.68	67.36	94.46	56.40	3.65	52.75	78.31
Burundi	BDI	2014	AFR	LIC	65.15	0.88	64.27	87.83	55.20	3.57	51.63	80.33
Burundi	BDI	2015	AFR	LIC	62.43	0.62	61.81	83.48	51.65	3.34	48.32	78.16
Burundi	BDI	2016	AFR	LIC	68.63	1.00	67.62	94.47	58.48	3.78	54.70	80.89
Burundi	BDI	2017	AFR	LIC	70.79	0.97	69.82	98.97	60.05	3.88	56.17	80.45
Burundi	BDI	2018	AFR	LIC	65.74	1.62	64.13	87.85	57.98	3.75	54.23	84.57
Burundi	BDI	2019	AFR	LIC	61.54	0.69	60.85	81.34	51.46	3.33	48.14	79.11
Benin	BEN	2010	AFR	LMIC	66.42	0.58	65.83	83.87	54.02	3.49	50.53	76.75
Benin	BEN	2011	AFR	LMIC	70.87	0.46	70.41	97.02	53.21	3.44	49.77	70.69
Benin	BEN	2012	AFR	LMIC	66.75	0.47	66.28	88.15	50.55	3.27	47.29	71.34
Benin	BEN	2013	AFR	LMIC	64.52	0.44	64.08	83.83	48.66	3.15	45.51	71.02
Benin	BEN	2014	AFR	LMIC	65.18	0.37	64.81	85.97	48.29	3.12	45.17	69.69
Benin	BEN	2015	AFR	LMIC	68.03	0.53	67.50	90.35	52.06	3.36	48.69	72.14
Benin	BEN	2016	AFR	LMIC	64.50	0.29	64.21	85.95	46.74	3.02	43.72	68.09
Benin	BEN	2017	AFR	LMIC	58.21	0.26	57.95	73.33	42.25	2.73	39.52	68.20
Benin	BEN	2018	AFR	LMIC	66.86	0.50	66.37	88.16	50.93	3.29	47.64	71.78
Benin	BEN	2019	AFR	LMIC	70.59	0.74	69.85	94.80	55.46	3.58	51.88	74.26
Burkina Faso	BFA	2010	AFR	LIC	55.80	0.23	55.57	73.25	40.17	2.60	37.57	67.61
Burkina Faso	BFA	2011	AFR	LIC	62.61	0.48	62.13	83.41	47.86	3.09	44.76	72.05
Burkina Faso	BFA	2012	AFR	LIC	63.54	0.63	62.91	81.40	52.55	3.40	49.15	78.13
Burkina Faso	BFA	2013	AFR	LIC	64.13	0.56	63.57	83.45	52.02	3.36	48.66	76.55
Burkina Faso	BFA	2014	AFR	LIC	65.47	0.59	64.89	85.53	53.55	3.46	50.09	77.19
Burkina Faso	BFA	2015	AFR	LIC	71.26	2.71	68.55	87.71	68.82	4.45	64.37	93.90
Burkina Faso	BFA	2016	AFR	LIC	67.37	0.89	66.48	87.64	56.99	3.68	53.30	80.18
Burkina Faso	BFA	2017	AFR	LIC	66.97	1.81	65.16	85.57	59.54	3.85	55.69	85.46
Burkina Faso	BFA	2018	AFR	LIC	70.90	1.05	69.85	94.00	60.50	3.91	56.59	81.02
Burkina Faso	BFA	2019	AFR	LIC	56.09	0.50	55.59	69.36	45.05	2.91	42.14	75.80

Bangladesh	BGD	2010	SEA	LMIC	55.78	2.10	53.68	33.08	53.76	3.47	50.28	93.68
Bangladesh	BGD	2011	SEA	LMIC	56.43	2.51	53.92	33.08	56.62	3.66	52.96	98.21
Bangladesh	BGD	2012	SEA	LMIC	60.91	2.48	58.43	38.75	59.91	3.87	56.03	95.90
Bangladesh	BGD	2013	SEA	LMIC	66.37	3.25	63.12	44.87	68.14	4.40	63.74	100.97
Bangladesh	BGD	2014	SEA	LMIC	67.13	2.89	64.23	46.46	66.90	4.32	62.58	97.42
Bangladesh	BGD	2015	SEA	LMIC	68.13	1.89	66.24	53.06	60.71	3.92	56.78	85.72
Bangladesh	BGD	2016	SEA	LMIC	72.66	1.98	70.68	60.13	64.64	4.18	60.46	85.55
Bangladesh	BGD	2017	SEA	LMIC	79.69	2.31	77.38	69.58	72.67	4.70	67.97	87.84
Bangladesh	BGD	2018	SEA	LMIC	86.19	2.46	83.73	81.78	78.08	5.05	73.03	87.22
Bangladesh	BGD	2019	SEA	LMIC	92.16	2.67	89.49	94.91	82.57	5.34	77.23	86.30
Bulgaria	BGR	2010	EUR	UMIC	72.73	0.64	72.09	99.88	49.87	3.22	46.64	64.70
Bulgaria	BGR	2011	EUR	UMIC	72.73	0.62	72.11	99.88	49.77	3.22	46.55	64.56
Bulgaria	BGR	2012	EUR	UMIC	72.73	0.52	72.21	99.87	49.16	3.18	45.99	63.69
Bulgaria	BGR	2013	EUR	UMIC	72.73	0.61	72.12	99.88	49.72	3.21	46.50	64.48
Bulgaria	BGR	2014	EUR	UMIC	72.73	0.42	72.31	99.86	48.08	3.11	44.97	62.19
Bulgaria	BGR	2015	EUR	UMIC	72.73	0.47	72.26	99.87	48.71	3.15	45.56	63.06
Bulgaria	BGR	2016	EUR	UMIC	72.73	0.45	72.28	99.86	48.51	3.14	45.38	62.78
Bulgaria	BGR	2017	EUR	UMIC	72.73	0.40	72.33	99.86	47.85	3.09	44.76	61.89
Bulgaria	BGR	2018	EUR	UMIC	72.73	0.38	72.35	99.85	47.36	3.06	44.30	61.23
Bulgaria	BGR	2019	EUR	UMIC	72.73	0.39	72.34	99.85	47.64	3.08	44.56	61.60
Bosnia and Herzegovina	BIH	2010	EUR	UMIC	80.17	0.25	79.93	98.60	55.70	3.60	52.10	65.18
Bosnia and Herzegovina	BIH	2011	EUR	UMIC	72.73	0.70	72.03	98.51	50.13	3.24	46.89	65.10
Bosnia and Herzegovina	BIH	2012	EUR	UMIC	72.73	0.43	72.30	98.47	48.23	3.12	45.11	62.40
Bosnia and Herzegovina	BIH	2013	EUR	UMIC	72.73	0.41	72.32	98.47	47.93	3.10	44.83	61.99
Bosnia and Herzegovina	BIH	2014	EUR	UMIC	72.73	0.37	72.36	98.46	47.12	3.05	44.07	60.91
Bosnia and Herzegovina	BIH	2015	EUR	UMIC	72.73	0.38	72.35	98.46	47.28	3.06	44.23	61.13
Bosnia and Herzegovina	BIH	2016	EUR	UMIC	72.73	0.36	72.37	98.44	46.52	3.01	43.51	60.12
Bosnia and Herzegovina	BIH	2017	EUR	UMIC	72.73	0.38	72.34	98.46	47.50	3.07	44.43	61.41
Bosnia and Herzegovina	BIH	2018	EUR	UMIC	74.07	0.78	73.29	98.55	51.85	3.35	48.50	66.18
Bosnia and Herzegovina	BIH	2019	EUR	UMIC	73.52	0.95	72.57	98.55	51.60	3.34	48.26	66.51
Belarus	BLR	2010	EUR	UMIC	72.73	0.35	72.38	97.33	46.35	3.00	43.35	59.90
Belarus	BLR	2011	EUR	UMIC	72.73	0.36	72.36	97.34	46.89	3.03	43.86	60.61
Belarus	BLR	2012	EUR	UMIC	72.73	0.36	72.36	97.34	46.90	3.03	43.87	60.62
Belarus	BLR	2013	EUR	UMIC	72.73	0.36	72.37	97.34	46.60	3.01	43.59	60.23
Belarus	BLR	2014	EUR	UMIC	72.73	0.34	72.38	97.33	45.96	2.97	42.99	59.39
Belarus	BLR	2015	EUR	UMIC	72.73	0.37	72.36	97.35	47.02	3.04	43.98	60.78
Belarus	BLR	2016	EUR	UMIC	78.31	0.29	78.02	97.47	54.27	3.51	50.76	65.07
Belarus	BLR	2017	EUR	UMIC	80.44	0.24	80.20	97.50	55.93	3.62	52.32	65.24
Belarus	BLR	2018	EUR	UMIC	80.97	0.24	80.73	97.50	56.42	3.65	52.78	65.37
Belarus	BLR	2019	EUR	UMIC	72.73	0.31	72.41	97.29	44.45	2.87	41.58	57.42
Belize	BLZ	2010	AMR	UMIC	90.53	0.30	90.22	98.58	64.23	4.15	60.08	66.59
Belize	BLZ	2011	AMR	UMIC	93.88	0.71	93.17	98.68	71.67	4.63	67.04	71.95
Belize	BLZ	2012	AMR	UMIC	87.33	0.27	87.06	98.52	60.69	3.92	56.77	65.21
Belize	BLZ	2013	AMR	UMIC	90.61	0.31	90.30	98.58	64.35	4.16	60.19	66.65
Belize	BLZ	2014	AMR	UMIC	90.95	0.33	90.63	98.59	64.86	4.19	60.67	66.95
Belize	BLZ	2015	AMR	UMIC	90.44	0.30	90.14	98.58	64.11	4.14	59.96	66.52
Belize	BLZ	2016	AMR	UMIC	79.09	0.86	78.24	98.45	54.85	3.55	51.30	65.58
Belize	BLZ	2017	AMR	UMIC	81.00	0.74	80.26	98.46	56.61	3.66	52.95	65.98

Belize	BLZ	2018	AMR	UMIC	83.85	0.39	83.46	98.49	58.17	3.76	54.41	65.19
Belize	BLZ	2019	AMR	UMIC	82.06	0.55	81.51	98.48	57.15	3.69	53.46	65.58
Bolivia (Plurinational State of)	BOL	2010	AMR	LMIC	68.11	0.62	67.49	93.08	54.58	3.53	51.05	75.64
Bolivia (Plurinational State of)	BOL	2011	AMR	LMIC	68.98	0.50	68.47	97.44	52.42	3.39	49.03	71.61
Bolivia (Plurinational State of)	BOL	2012	AMR	LMIC	68.00	0.52	67.48	95.23	51.95	3.36	48.59	72.01
Bolivia (Plurinational State of)	BOL	2013	AMR	LMIC	69.39	0.65	68.74	97.45	54.06	3.49	50.56	73.56
Bolivia (Plurinational State of)	BOL	2014	AMR	LMIC	67.87	0.49	67.38	95.23	51.49	3.33	48.17	71.49
Bolivia (Plurinational State of)	BOL	2015	AMR	LMIC	65.72	0.47	65.25	90.87	49.89	3.22	46.66	71.52
Bolivia (Plurinational State of)	BOL	2016	AMR	LMIC	65.84	0.50	65.33	90.87	50.30	3.25	47.05	72.01
Bolivia (Plurinational State of)	BOL	2017	AMR	LMIC	65.38	0.41	64.98	90.86	48.83	3.16	45.68	70.30
Bolivia (Plurinational State of)	BOL	2018	AMR	LMIC	65.26	0.27	64.99	93.00	46.96	3.04	43.92	67.58
Bolivia (Plurinational State of)	BOL	2019	AMR	LMIC	64.04	0.25	63.79	90.83	45.90	2.97	42.94	67.31
Brazil	BRA	2010	AMR	UMIC	93.15	0.56	92.59	98.31	69.37	4.48	64.89	70.08
Brazil	BRA	2011	AMR	UMIC	92.15	0.42	91.72	98.27	66.98	4.33	62.65	68.30
Brazil	BRA	2012	AMR	UMIC	92.89	0.52	92.37	98.30	68.68	4.44	64.24	69.54
Brazil	BRA	2013	AMR	UMIC	92.67	0.49	92.19	98.29	68.14	4.40	63.73	69.14
Brazil	BRA	2014	AMR	UMIC	92.68	0.49	92.19	98.29	68.16	4.41	63.75	69.15
Brazil	BRA	2015	AMR	UMIC	93.46	0.62	92.84	98.32	70.25	4.54	65.71	70.78
Brazil	BRA	2016	AMR	UMIC	93.94	0.73	93.21	98.35	71.88	4.65	67.23	72.13
Brazil	BRA	2017	AMR	UMIC	94.08	0.77	93.31	98.36	72.43	4.68	67.74	72.61
Brazil	BRA	2018	AMR	UMIC	94.84	0.99	93.85	98.40	75.13	4.86	70.27	74.88
Brazil	BRA	2019	AMR	UMIC	95.28	0.90	94.38	98.41	75.97	4.91	71.06	75.30
Bhutan	BTN	2010	SEA	LMIC	82.92	0.27	82.65	94.97	58.61	3.79	54.82	66.33
Bhutan	BTN	2011	SEA	LMIC	80.87	0.24	80.63	94.93	56.33	3.64	52.69	65.34
Bhutan	BTN	2012	SEA	LMIC	84.83	0.40	84.43	95.01	61.79	3.99	57.80	68.45
Bhutan	BTN	2013	SEA	LMIC	84.66	0.38	84.27	95.00	61.44	3.97	57.46	68.19
Bhutan	BTN	2014	SEA	LMIC	82.93	0.27	82.67	94.97	58.63	3.79	54.84	66.34
Bhutan	BTN	2015	SEA	LMIC	83.14	0.28	82.86	94.97	58.91	3.81	55.11	66.50
Bhutan	BTN	2016	SEA	LMIC	80.32	0.24	80.07	94.93	55.83	3.61	52.22	65.21
Bhutan	BTN	2017	SEA	LMIC	82.70	0.26	82.44	94.96	58.33	3.77	54.56	66.18
Bhutan	BTN	2018	SEA	LMIC	83.25	0.28	82.97	94.97	59.08	3.82	55.26	66.60
Bhutan	BTN	2019	SEA	LMIC	83.73	0.31	83.42	94.98	59.79	3.86	55.93	67.04
Botswana	BWA	2010	AFR	UMIC	66.56	0.25	66.30	72.83	46.12	2.98	43.14	65.07
Botswana	BWA	2011	AFR	UMIC	69.01	0.20	68.80	72.85	48.14	3.11	45.02	65.44
Botswana	BWA	2012	AFR	UMIC	74.89	0.23	74.66	87.45	51.99	3.36	48.63	65.14
Botswana	BWA	2013	AFR	UMIC	80.99	0.24	80.75	98.65	56.44	3.65	52.79	65.38
Botswana	BWA	2014	AFR	UMIC	73.37	0.28	73.09	87.44	50.85	3.29	47.56	65.07
Botswana	BWA	2015	AFR	UMIC	62.95	0.34	62.61	68.87	43.74	2.83	40.91	65.35
Botswana	BWA	2016	AFR	UMIC	78.53	0.23	78.30	91.87	54.91	3.55	51.36	65.59
Botswana	BWA	2017	AFR	UMIC	79.71	0.25	79.46	98.63	55.31	3.58	51.74	65.12
Botswana	BWA	2018	AFR	UMIC	78.83	0.31	78.52	87.51	56.61	3.66	52.95	67.44
Botswana	BWA	2019	AFR	UMIC	52.22	0.18	52.04	45.75	36.19	2.34	33.85	65.06
Central African Republic	CAF	2010	AFR	LIC	33.44	1.36	32.08	52.35	32.89	2.13	30.77	95.90
Central African Republic	CAF	2011	AFR	LIC	27.62	0.79	26.83	43.95	25.07	1.62	23.45	87.39
Central African Republic	CAF	2012	AFR	LIC	37.49	0.33	37.16	65.14	30.25	1.96	28.29	76.14
Central African Republic	CAF	2013	AFR	LIC	40.55	0.64	39.91	69.52	34.73	2.24	32.49	81.39
Central African Republic	CAF	2014	AFR	LIC	47.44	0.92	46.53	82.76	41.13	2.66	38.47	82.69

Central African Republic	CAF	2015	AFR	LIC	51.10	1.96	49.14	85.04	49.43	3.20	46.24	94.08
Central African Republic	CAF	2016	AFR	LIC	48.93	1.51	47.42	82.79	45.24	2.92	42.31	89.22
Central African Republic	CAF	2017	AFR	LIC	45.01	1.30	43.71	76.13	41.01	2.65	38.36	87.75
Central African Republic	CAF	2018	AFR	LIC	48.74	1.20	47.54	85.00	42.99	2.78	40.21	84.58
Central African Republic	CAF	2019	AFR	LIC	54.16	1.55	52.61	94.01	49.24	3.18	46.06	87.54
China	CHN	2010	WPR	UMIC	100.00	2.91	97.09	81.21	89.48	5.78	83.70	86.21
China	CHN	2011	WPR	UMIC	99.11	1.54	97.57	81.17	84.81	5.48	79.33	81.31
China	CHN	2012	WPR	UMIC	99.07	1.50	97.57	81.17	84.67	5.47	79.20	81.17
China	CHN	2013	WPR	UMIC	98.02	0.95	97.08	81.14	80.92	5.23	75.69	77.97
China	CHN	2014	WPR	UMIC	98.19	0.99	97.20	81.14	81.43	5.26	76.17	78.37
China	CHN	2015	WPR	UMIC	98.42	1.08	97.34	81.15	82.21	5.31	76.89	78.99
China	CHN	2016	WPR	UMIC	96.60	0.87	95.72	78.57	79.10	5.11	73.99	77.29
China	CHN	2017	WPR	UMIC	95.93	0.84	95.09	78.55	77.40	5.00	72.40	76.14
China	CHN	2018	WPR	UMIC	100.00	1.12	98.88	91.87	78.91	5.10	73.81	74.65
China	CHN	2019	WPR	UMIC	94.15	0.80	93.35	78.50	72.71	4.70	68.01	72.86
Cote d'Ivoire	CIV	2010	AFR	LMIC	63.27	1.24	62.03	74.16	54.90	3.55	51.35	82.79
Cote d'Ivoire	CIV	2011	AFR	LMIC	63.38	0.59	62.79	76.14	52.07	3.37	48.70	77.57
Cote d'Ivoire	CIV	2012	AFR	LMIC	73.14	2.57	70.57	86.50	69.35	4.48	64.87	91.93
Cote d'Ivoire	CIV	2013	AFR	LMIC	73.62	0.74	72.88	94.96	61.00	3.94	57.06	78.29
Cote d'Ivoire	CIV	2014	AFR	LMIC	69.51	0.86	68.65	86.43	58.50	3.78	54.72	79.70
Cote d'Ivoire	CIV	2015	AFR	LMIC	70.74	2.10	68.64	84.39	64.88	4.19	60.69	88.42
Cote d'Ivoire	CIV	2016	AFR	LMIC	64.38	0.66	63.73	80.18	51.07	3.30	47.77	74.96
Cote d'Ivoire	CIV	2017	AFR	LMIC	63.11	0.69	62.42	78.14	49.87	3.22	46.65	74.73
Cote d'Ivoire	CIV	2018	AFR	LMIC	65.62	0.61	65.00	80.20	53.95	3.49	50.46	77.63
Cote d'Ivoire	CIV	2019	AFR	LMIC	66.10	0.58	65.51	82.24	53.24	3.44	49.80	76.01
Cameroon	CMR	2010	AFR	LMIC	53.59	2.10	51.48	79.29	52.17	3.37	48.79	94.78
Cameroon	CMR	2011	AFR	LMIC	53.44	1.55	51.89	83.69	47.85	3.09	44.75	86.25
Cameroon	CMR	2012	AFR	LMIC	54.54	1.57	52.97	85.92	48.75	3.15	45.60	86.10
Cameroon	CMR	2013	AFR	LMIC	56.04	0.68	55.36	90.39	47.13	3.05	44.09	79.64
Cameroon	CMR	2014	AFR	LMIC	60.97	2.00	58.97	94.98	57.03	3.69	53.34	90.46
Cameroon	CMR	2015	AFR	LMIC	62.17	2.05	60.12	97.27	58.22	3.76	54.46	90.58
Cameroon	CMR	2016	AFR	LMIC	59.79	1.05	58.74	97.23	51.54	3.33	48.21	82.07
Cameroon	CMR	2017	AFR	LMIC	57.69	1.23	56.46	92.67	50.32	3.25	47.07	83.37
Cameroon	CMR	2018	AFR	LMIC	57.87	1.70	56.17	90.43	52.92	3.42	49.50	88.13
Cameroon	CMR	2019	AFR	LMIC	60.22	1.75	58.48	97.24	53.86	3.48	50.38	86.16
Democratic Republic of the Congo	COD	2010	AFR	LIC	61.27	1.61	59.66	69.32	54.32	3.51	50.81	85.17
Democratic Republic of the Congo	COD	2011	AFR	LIC	57.08	1.63	55.44	62.11	51.30	3.32	47.99	86.56
Democratic Republic of the Congo	COD	2012	AFR	LIC	55.06	1.57	53.49	58.59	49.76	3.22	46.55	87.02
Democratic Republic of the Congo	COD	2013	AFR	LIC	54.20	1.02	53.18	58.58	46.92	3.03	43.88	82.52
Democratic Republic of the Congo	COD	2014	AFR	LIC	54.24	1.08	53.16	58.58	47.10	3.04	44.06	82.87
Democratic Republic of the Congo	COD	2015	AFR	LIC	53.52	0.53	52.99	58.56	44.31	2.86	41.45	78.22
Democratic Republic of the Congo	COD	2016	AFR	LIC	57.53	1.00	56.53	63.87	49.56	3.20	46.36	82.02
Democratic Republic of the Congo	COD	2017	AFR	LIC	66.35	2.03	64.32	74.91	61.23	3.96	57.27	89.05
Democratic Republic of the Congo	COD	2018	AFR	LIC	73.92	2.44	71.48	86.43	69.26	4.48	64.79	90.64
Democratic Republic of the Congo	COD	2019	AFR	LIC	75.10	2.48	72.61	88.39	70.37	4.55	65.82	90.64
Congo	COG	2010	AFR	LMIC	69.60	0.91	68.69	87.99	58.82	3.80	55.02	80.11
Congo	COG	2011	AFR	LMIC	71.67	0.63	71.04	94.42	57.82	3.74	54.08	76.13

Congo	COG	2012	AFR	LMIC	68.12	0.21	67.90	96.49	47.98	3.10	44.88	66.09
Congo	COG	2013	AFR	LMIC	63.59	0.22	63.37	85.76	45.20	2.92	42.28	66.72
Congo	COG	2014	AFR	LMIC	63.34	1.35	61.99	75.57	55.27	3.57	51.70	83.39
Congo	COG	2015	AFR	LMIC	66.20	4.58	61.62	71.67	76.46	4.94	71.51	116.05
Congo	COG	2016	AFR	LMIC	65.10	2.38	62.72	73.60	62.26	4.02	58.24	92.85
Congo	COG	2017	AFR	LMIC	61.29	2.14	59.16	67.67	58.02	3.75	54.27	91.73
Congo	COG	2018	AFR	LMIC	63.48	2.14	61.34	71.60	59.72	3.86	55.86	91.07
Congo	COG	2019	AFR	LMIC	64.09	0.68	63.41	81.66	50.40	3.26	47.14	74.35
Colombia	COL	2010	AMR	UMIC	82.64	0.26	82.38	97.71	58.25	3.76	54.48	66.14
Colombia	COL	2011	AMR	UMIC	81.90	0.24	81.66	97.70	57.38	3.71	53.67	65.73
Colombia	COL	2012	AMR	UMIC	81.30	0.24	81.06	97.69	56.75	3.67	53.08	65.48
Colombia	COL	2013	AMR	UMIC	81.39	0.24	81.15	97.70	56.84	3.67	53.16	65.51
Colombia	COL	2014	AMR	UMIC	77.95	0.31	77.63	97.66	54.02	3.49	50.53	65.09
Colombia	COL	2015	AMR	UMIC	81.02	0.24	80.78	97.69	56.47	3.65	52.82	65.39
Colombia	COL	2016	AMR	UMIC	81.26	0.24	81.02	97.69	56.70	3.67	53.04	65.47
Colombia	COL	2017	AMR	UMIC	80.87	0.24	80.63	97.68	56.33	3.64	52.69	65.34
Colombia	COL	2018	AMR	UMIC	81.10	0.24	80.86	97.69	56.55	3.66	52.89	65.41
Colombia	COL	2019	AMR	UMIC	77.78	0.32	77.46	97.64	53.92	3.48	50.43	65.10
Comoros	COM	2010	AFR	LMIC	46.08	0.23	45.85	74.79	31.98	2.07	29.92	65.24
Comoros	COM	2011	AFR	LMIC	44.97	0.35	44.62	74.78	31.37	2.03	29.35	65.77
Comoros	COM	2012	AFR	LMIC	45.63	0.26	45.36	74.79	31.72	2.05	29.67	65.41
Comoros	COM	2013	AFR	LMIC	45.17	0.32	44.86	74.78	31.48	2.03	29.44	65.64
Comoros	COM	2014	AFR	LMIC	54.28	0.23	54.04	92.76	37.63	2.43	35.20	65.13
Comoros	COM	2015	AFR	LMIC	56.85	0.18	56.67	95.10	39.45	2.55	36.90	65.12
Comoros	COM	2016	AFR	LMIC	59.76	0.18	59.58	99.79	41.65	2.69	38.96	65.39
Comoros	COM	2017	AFR	LMIC	43.84	0.18	43.67	68.35	30.39	1.96	28.42	65.09
Comoros	COM	2018	AFR	LMIC	41.80	0.13	41.66	62.08	29.00	1.87	27.13	65.11
Comoros	COM	2019	AFR	LMIC	41.88	0.13	41.75	62.08	29.07	1.88	27.19	65.12
Cabo Verde	CPV	2010	AFR	LMIC	90.09	1.39	88.70	98.68	77.06	4.98	72.08	81.26
Cabo Verde	CPV	2011	AFR	LMIC	90.04	1.34	88.69	98.67	76.84	4.97	71.88	81.04
Cabo Verde	CPV	2012	AFR	LMIC	90.46	1.88	88.58	98.69	78.79	5.09	73.70	83.20
Cabo Verde	CPV	2013	AFR	LMIC	89.45	0.97	88.48	98.66	74.65	4.82	69.82	78.92
Cabo Verde	CPV	2014	AFR	LMIC	86.93	0.95	85.98	98.60	68.49	4.43	64.06	74.51
Cabo Verde	CPV	2015	AFR	LMIC	88.15	0.78	87.37	98.62	71.03	4.59	66.44	76.04
Cabo Verde	CPV	2016	AFR	LMIC	87.89	0.80	87.10	98.62	70.45	4.55	65.90	75.66
Cabo Verde	CPV	2017	AFR	LMIC	86.87	0.90	85.97	98.60	68.17	4.41	63.76	74.17
Cabo Verde	CPV	2018	AFR	LMIC	86.54	0.72	85.82	98.58	66.71	4.31	62.40	72.71
Cabo Verde	CPV	2019	AFR	LMIC	86.53	0.72	85.81	98.58	66.69	4.31	62.38	72.69
Cuba	CUB	2010	AMR	UMIC	79.09	0.31	78.78	99.88	44.28	2.86	41.42	52.58
Cuba	CUB	2011	AMR	UMIC	79.09	0.31	78.78	99.89	44.80	2.90	41.91	53.20
Cuba	CUB	2012	AMR	UMIC	79.09	0.31	78.78	99.88	44.94	2.91	42.04	53.36
Cuba	CUB	2013	AMR	UMIC	79.09	0.32	78.78	99.90	45.82	2.96	42.86	54.41
Cuba	CUB	2014	AMR	UMIC	79.09	0.32	78.77	99.95	47.33	3.06	44.27	56.20
Cuba	CUB	2015	AMR	UMIC	79.09	0.32	78.77	99.96	47.25	3.05	44.19	56.10
Cuba	CUB	2016	AMR	UMIC	79.09	0.32	78.77	99.93	47.03	3.04	43.99	55.85
Cuba	CUB	2017	AMR	UMIC	90.25	0.29	89.96	99.96	63.84	4.13	59.72	66.38
Cuba	CUB	2018	AMR	UMIC	89.31	0.27	89.05	99.97	62.68	4.05	58.63	65.84
Cuba	CUB	2019	AMR	UMIC	89.30	0.27	89.03	99.97	62.66	4.05	58.61	65.83

Djibouti	DJI	2010	EMR	LMIC	94.48	3.37	91.11	94.10	89.85	5.81	84.04	92.24
Djibouti	DJI	2011	EMR	LMIC	94.44	3.35	91.09	94.10	89.71	5.80	83.92	92.12
Djibouti	DJI	2012	EMR	LMIC	95.02	3.61	91.40	94.12	91.77	5.93	85.83	93.91
Djibouti	DJI	2013	EMR	LMIC	93.96	3.14	90.82	94.09	88.23	5.70	82.53	90.87
Djibouti	DJI	2014	EMR	LMIC	86.98	0.97	86.01	93.94	68.66	4.44	64.23	74.67
Djibouti	DJI	2015	EMR	LMIC	86.62	0.76	85.86	93.93	67.06	4.33	62.72	73.05
Djibouti	DJI	2016	EMR	LMIC	87.02	0.96	86.06	93.94	68.73	4.44	64.29	74.70
Djibouti	DJI	2017	EMR	LMIC	85.46	0.49	84.98	93.88	63.27	4.09	59.18	69.64
Djibouti	DJI	2018	EMR	LMIC	86.87	0.90	85.97	93.94	68.17	4.41	63.76	74.17
Djibouti	DJI	2019	EMR	LMIC	85.48	0.49	84.99	93.88	63.31	4.09	59.21	69.67
Dominican Republic	DOM	2010	AMR	UMIC	82.70	0.26	82.44	98.22	58.33	3.77	54.56	66.18
Dominican Republic	DOM	2011	AMR	UMIC	79.76	0.25	79.51	98.18	55.35	3.58	51.78	65.12
Dominican Republic	DOM	2012	AMR	UMIC	78.50	0.29	78.21	98.16	54.40	3.52	50.88	65.06
Dominican Republic	DOM	2013	AMR	UMIC	79.90	0.25	79.65	98.18	55.47	3.59	51.89	65.14
Dominican Republic	DOM	2014	AMR	UMIC	76.90	0.37	76.53	98.14	53.36	3.45	49.92	65.22
Dominican Republic	DOM	2015	AMR	UMIC	75.59	0.49	75.11	98.13	52.62	3.40	49.22	65.54
Dominican Republic	DOM	2016	AMR	UMIC	77.63	0.33	77.30	98.15	53.81	3.48	50.34	65.12
Dominican Republic	DOM	2017	AMR	UMIC	75.73	0.47	75.26	98.13	52.70	3.41	49.29	65.50
Dominican Republic	DOM	2018	AMR	UMIC	73.21	1.06	72.15	98.11	51.46	3.33	48.13	66.71
Dominican Republic	DOM	2019	AMR	UMIC	73.64	0.91	72.73	98.12	51.65	3.34	48.31	66.43
Ecuador	ECU	2010	AMR	UMIC	83.85	0.32	83.53	98.39	59.98	3.88	56.10	67.17
Ecuador	ECU	2011	AMR	UMIC	81.90	0.24	81.65	98.35	57.38	3.71	53.67	65.73
Ecuador	ECU	2012	AMR	UMIC	83.04	0.27	82.77	98.37	58.78	3.80	54.98	66.43
Ecuador	ECU	2013	AMR	UMIC	84.06	0.33	83.73	98.40	60.34	3.90	56.44	67.41
Ecuador	ECU	2014	AMR	UMIC	83.61	0.30	83.31	98.39	59.60	3.85	55.75	66.92
Ecuador	ECU	2015	AMR	UMIC	82.58	0.26	82.33	98.37	58.18	3.76	54.42	66.10
Ecuador	ECU	2016	AMR	UMIC	82.85	0.26	82.59	98.38	58.52	3.78	54.74	66.28
Ecuador	ECU	2017	AMR	UMIC	82.39	0.25	82.14	98.37	57.94	3.75	54.20	65.99
Ecuador	ECU	2018	AMR	UMIC	82.95	0.27	82.69	98.38	58.66	3.79	54.87	66.36
Ecuador	ECU	2019	AMR	UMIC	81.90	0.24	81.65	98.36	57.37	3.71	53.67	65.73
Eritrea	ERI	2010	AFR	LIC	65.22	0.38	64.84	94.58	48.39	3.13	45.26	69.80
Eritrea	ERI	2011	AFR	LIC	62.66	0.19	62.48	94.53	44.00	2.84	41.15	65.87
Eritrea	ERI	2012	AFR	LIC	64.42	0.28	64.14	94.56	46.58	3.01	43.57	67.93
Eritrea	ERI	2013	AFR	LIC	64.78	0.32	64.46	94.56	47.32	3.06	44.26	68.66
Eritrea	ERI	2014	AFR	LIC	65.15	0.37	64.78	94.57	48.19	3.12	45.08	69.58
Eritrea	ERI	2015	AFR	LIC	65.15	0.37	64.78	94.57	48.19	3.12	45.08	69.58
Eritrea	ERI	2016	AFR	LIC	65.40	0.41	64.99	94.58	48.89	3.16	45.73	70.36
Eritrea	ERI	2017	AFR	LIC	66.00	0.56	65.44	94.60	50.93	3.29	47.64	72.79
Eritrea	ERI	2018	AFR	LIC	66.26	0.70	65.56	94.61	52.09	3.37	48.72	74.33
Eritrea	ERI	2019	AFR	LIC	66.20	0.66	65.54	94.61	51.81	3.35	48.46	73.94
Ethiopia	ETH	2010	AFR	LIC	73.51	0.71	72.80	65.66	60.68	3.92	56.76	77.96
Ethiopia	ETH	2011	AFR	LIC	76.70	0.71	75.99	70.12	62.98	4.07	58.91	77.52
Ethiopia	ETH	2012	AFR	LIC	72.96	0.64	72.32	65.65	59.17	3.82	55.35	76.52
Ethiopia	ETH	2013	AFR	LIC	68.40	0.60	67.80	59.85	55.24	3.57	51.67	76.21
Ethiopia	ETH	2014	AFR	LIC	64.09	0.68	63.41	55.61	50.39	3.26	47.13	74.33
Ethiopia	ETH	2015	AFR	LIC	76.90	0.70	76.21	71.62	61.64	3.98	57.65	75.65
Ethiopia	ETH	2016	AFR	LIC	74.55	0.69	73.86	68.61	59.55	3.85	55.70	75.41

Ethiopia	ETH	2017	AFR	LIC	72.82	0.81	72.01	67.12	57.43	3.71	53.71	74.59
Ethiopia	ETH	2018	AFR	LIC	75.85	0.68	75.17	70.11	60.86	3.93	56.92	75.73
Ethiopia	ETH	2019	AFR	LIC	78.29	0.69	77.60	73.14	63.17	4.08	59.09	76.14
Fiji	FJI	2010	WPR	UMIC	72.73	0.52	72.21	98.93	49.17	3.18	45.99	63.69
Fiji	FJI	2011	WPR	UMIC	72.73	0.34	72.39	98.86	45.66	2.95	42.71	59.00
Fiji	FJI	2012	WPR	UMIC	72.73	0.41	72.31	98.91	48.03	3.10	44.92	62.12
Fiji	FJI	2013	WPR	UMIC	72.73	0.36	72.37	98.88	46.70	3.02	43.68	60.36
Fiji	FJI	2014	WPR	UMIC	72.73	0.41	72.32	98.91	47.92	3.10	44.82	61.98
Fiji	FJI	2015	WPR	UMIC	72.73	0.56	72.17	98.94	49.43	3.20	46.24	64.07
Fiji	FJI	2016	WPR	UMIC	72.73	0.74	71.98	98.96	50.30	3.25	47.05	65.36
Fiji	FJI	2017	WPR	UMIC	72.73	1.02	71.71	98.97	50.99	3.30	47.69	66.51
Fiji	FJI	2018	WPR	UMIC	79.68	0.25	79.43	99.05	55.29	3.57	51.72	65.11
Fiji	FJI	2019	WPR	UMIC	83.01	0.27	82.74	99.10	58.74	3.80	54.95	66.40
Micronesia (Federated States of)	FSM	2010	WPR	LMIC	90.83	2.49	88.34	99.74	80.85	5.23	75.63	85.61
Micronesia (Federated States of)	FSM	2011	WPR	LMIC	80.31	0.24	80.07	99.47	55.82	3.61	52.21	65.21
Micronesia (Federated States of)	FSM	2012	WPR	LMIC	93.61	3.00	90.61	99.78	87.22	5.64	81.58	90.04
Micronesia (Federated States of)	FSM	2013	WPR	LMIC	85.55	0.50	85.05	99.58	63.49	4.10	59.39	69.83
Micronesia (Federated States of)	FSM	2014	WPR	LMIC	84.66	0.38	84.28	99.55	61.45	3.97	57.48	68.20
Micronesia (Federated States of)	FSM	2015	WPR	LMIC	82.38	0.25	82.13	99.50	57.93	3.74	54.19	65.98
Micronesia (Federated States of)	FSM	2016	WPR	LMIC	83.35	0.29	83.06	99.52	59.21	3.83	55.38	66.68
Micronesia (Federated States of)	FSM	2017	WPR	LMIC	75.82	0.46	75.36	99.41	52.75	3.41	49.34	65.47
Micronesia (Federated States of)	FSM	2018	WPR	LMIC	81.46	0.24	81.22	99.48	56.91	3.68	53.23	65.54
Micronesia (Federated States of)	FSM	2019	WPR	LMIC	72.73	1.18	71.55	99.38	51.16	3.31	47.86	66.88
Gabon	GAB	2010	AFR	UMIC	46.89	0.48	46.41	68.84	38.95	2.52	36.43	78.50
Gabon	GAB	2011	AFR	UMIC	52.59	0.59	52.00	79.58	43.98	2.84	41.13	79.11
Gabon	GAB	2012	AFR	UMIC	55.85	0.53	55.32	88.39	44.53	2.88	41.65	75.29
Gabon	GAB	2013	AFR	UMIC	56.31	0.49	55.81	90.61	43.60	2.82	40.78	73.07
Gabon	GAB	2014	AFR	UMIC	59.16	0.41	58.75	97.39	44.66	2.89	41.77	71.10
Gabon	GAB	2015	AFR	UMIC	60.77	0.53	60.23	97.43	49.22	3.18	46.04	76.43
Gabon	GAB	2016	AFR	UMIC	57.85	0.55	57.31	90.65	47.61	3.08	44.53	77.70
Gabon	GAB	2017	AFR	UMIC	54.02	0.81	53.22	81.78	46.12	2.98	43.14	81.06
Gabon	GAB	2018	AFR	UMIC	55.53	0.60	54.93	88.38	43.92	2.84	41.08	74.78
Gabon	GAB	2019	AFR	UMIC	53.72	0.61	53.11	81.77	44.95	2.91	42.04	79.16
Georgia	GEO	2010	EUR	UMIC	92.67	0.28	92.38	96.03	65.16	4.21	60.95	65.97
Georgia	GEO	2011	EUR	UMIC	93.63	0.28	93.34	98.99	65.80	4.25	61.55	65.94
Georgia	GEO	2012	EUR	UMIC	84.22	0.25	83.97	74.36	59.13	3.82	55.30	65.86
Georgia	GEO	2013	EUR	UMIC	72.09	0.25	71.84	54.00	49.97	3.23	46.74	65.06
Georgia	GEO	2014	EUR	UMIC	70.45	0.51	69.94	58.13	49.12	3.17	45.94	65.69
Georgia	GEO	2015	EUR	UMIC	75.79	0.38	75.42	67.06	52.61	3.40	49.21	65.25
Georgia	GEO	2016	EUR	UMIC	77.90	0.37	77.53	71.83	54.05	3.49	50.56	65.22
Georgia	GEO	2017	EUR	UMIC	69.14	1.18	67.96	60.26	48.70	3.15	45.56	67.03
Georgia	GEO	2018	EUR	UMIC	66.69	1.02	65.67	53.96	46.90	3.03	43.87	66.81
Georgia	GEO	2019	EUR	UMIC	67.99	0.61	67.38	53.97	47.51	3.07	44.44	65.96
Ghana	GHA	2010	AFR	LMIC	34.39	0.23	34.16	49.18	25.90	1.67	24.23	70.91
Ghana	GHA	2011	AFR	LMIC	36.87	0.35	36.52	52.39	28.75	1.86	26.89	73.64
Ghana	GHA	2012	AFR	LMIC	35.55	0.26	35.29	50.78	26.97	1.74	25.23	71.49
Ghana	GHA	2013	AFR	LMIC	36.12	0.18	35.94	52.37	26.40	1.71	24.69	68.71

Ghana	GHA	2014	AFR	LMIC	35.49	0.24	35.24	50.78	26.77	1.73	25.04	71.04
Ghana	GHA	2015	AFR	LMIC	35.34	0.31	35.03	49.19	28.60	1.85	26.75	76.36
Ghana	GHA	2016	AFR	LMIC	32.31	0.10	32.21	49.15	22.49	1.45	21.04	65.32
Ghana	GHA	2017	AFR	LMIC	34.10	0.18	33.92	49.17	25.10	1.62	23.48	69.21
Ghana	GHA	2018	AFR	LMIC	34.77	0.38	34.39	49.19	27.38	1.77	25.61	74.46
Ghana	GHA	2019	AFR	LMIC	36.72	0.29	36.43	52.39	28.12	1.82	26.31	72.20
Guinea	GIN	2010	AFR	LIC	64.84	0.70	64.14	63.17	54.09	3.50	50.59	78.88
Guinea	GIN	2011	AFR	LIC	67.79	1.32	66.46	66.99	58.80	3.80	55.00	82.76
Guinea	GIN	2012	AFR	LIC	69.53	2.05	67.47	67.02	63.70	4.12	59.58	88.31
Guinea	GIN	2013	AFR	LIC	70.78	3.10	67.69	65.14	70.79	4.58	66.21	97.82
Guinea	GIN	2014	AFR	LIC	67.61	1.09	66.52	66.99	57.98	3.75	54.23	81.53
Guinea	GIN	2015	AFR	LIC	67.72	1.24	66.49	66.99	58.50	3.78	54.72	82.30
Guinea	GIN	2016	AFR	LIC	70.46	2.05	68.41	68.96	64.25	4.15	60.10	87.85
Guinea	GIN	2017	AFR	LIC	75.25	2.21	73.03	76.94	68.84	4.45	64.39	88.16
Guinea	GIN	2018	AFR	LIC	75.96	2.41	73.55	76.95	70.59	4.56	66.03	89.77
Guinea	GIN	2019	AFR	LIC	85.85	2.91	82.93	94.00	80.82	5.22	75.60	91.15
Guinea-Bissau	GNB	2010	AFR	LIC	44.32	0.39	43.93	97.55	36.11	2.33	33.78	76.90
Guinea-Bissau	GNB	2011	AFR	LIC	40.00	0.33	39.68	89.60	30.77	1.99	28.78	72.54
Guinea-Bissau	GNB	2012	AFR	LIC	35.94	0.38	35.56	79.13	28.43	1.84	26.59	74.80
Guinea-Bissau	GNB	2013	AFR	LIC	36.57	0.13	36.44	84.31	26.06	1.68	24.37	66.89
Guinea-Bissau	GNB	2014	AFR	LIC	38.99	0.16	38.83	89.56	28.11	1.82	26.29	67.71
Guinea-Bissau	GNB	2015	AFR	LIC	35.89	0.15	35.73	81.70	25.93	1.68	24.26	67.88
Guinea-Bissau	GNB	2016	AFR	LIC	37.61	0.25	37.36	84.34	28.29	1.83	26.46	70.84
Guinea-Bissau	GNB	2017	AFR	LIC	36.15	0.18	35.97	81.71	26.47	1.71	24.76	68.84
Guinea-Bissau	GNB	2018	AFR	LIC	31.93	0.16	31.77	71.31	23.45	1.52	21.93	69.03
Guinea-Bissau	GNB	2019	AFR	LIC	37.21	0.19	37.03	84.32	27.25	1.76	25.49	68.84
Equatorial Guinea	GNQ	2010	AFR	UMIC	60.91	0.24	60.67	99.42	33.00	2.13	30.87	50.88
Equatorial Guinea	GNQ	2011	AFR	UMIC	60.00	0.24	59.76	97.17	33.26	2.15	31.11	52.05
Equatorial Guinea	GNQ	2012	AFR	UMIC	59.09	0.23	58.86	94.93	32.73	2.12	30.62	52.02
Equatorial Guinea	GNQ	2013	AFR	UMIC	59.09	0.23	58.86	94.93	33.09	2.14	30.95	52.59
Equatorial Guinea	GNQ	2014	AFR	UMIC	58.18	0.23	57.95	92.74	34.02	2.20	31.82	54.91
Equatorial Guinea	GNQ	2015	AFR	UMIC	58.18	0.31	57.87	92.83	38.10	2.46	35.64	61.58
Equatorial Guinea	GNQ	2016	AFR	UMIC	61.42	0.39	61.02	95.13	42.76	2.76	39.99	65.54
Equatorial Guinea	GNQ	2017	AFR	UMIC	57.27	0.29	56.98	90.60	37.09	2.40	34.69	60.88
Equatorial Guinea	GNQ	2018	AFR	UMIC	51.53	0.64	50.89	75.69	36.15	2.34	33.81	66.44
Equatorial Guinea	GNQ	2019	AFR	UMIC	57.27	0.44	56.84	90.64	38.92	2.52	36.41	64.06
Guatemala	GTM	2010	AMR	UMIC	87.74	0.81	86.93	97.69	70.13	4.53	65.60	75.46
Guatemala	GTM	2011	AMR	UMIC	80.41	0.24	80.17	97.50	55.91	3.61	52.29	65.23
Guatemala	GTM	2012	AMR	UMIC	86.33	0.65	85.67	97.64	65.91	4.26	61.65	71.96
Guatemala	GTM	2013	AMR	UMIC	83.87	0.32	83.55	97.57	60.01	3.88	56.13	67.18
Guatemala	GTM	2014	AMR	UMIC	78.58	0.28	78.30	97.48	54.46	3.52	50.94	65.06
Guatemala	GTM	2015	AMR	UMIC	84.04	0.33	83.71	97.57	60.31	3.90	56.41	67.38
Guatemala	GTM	2016	AMR	UMIC	84.67	0.38	84.28	97.59	61.45	3.97	57.48	68.20
Guatemala	GTM	2017	AMR	UMIC	84.02	0.33	83.69	97.57	60.26	3.89	56.36	67.35
Guatemala	GTM	2018	AMR	UMIC	83.03	0.27	82.76	97.55	58.76	3.80	54.96	66.41
Guatemala	GTM	2019	AMR	UMIC	82.30	0.25	82.05	97.53	57.84	3.74	54.10	65.94
Guyana	GUY	2010	AMR	UMIC	84.89	0.41	84.48	98.24	61.91	4.00	57.91	68.55
Guyana	GUY	2011	AMR	UMIC	85.21	0.45	84.76	98.25	62.64	4.05	58.59	69.12

Guyana	GUY	2012	AMR	UMIC	83.75	0.31	83.44	98.21	59.82	3.87	55.96	67.06
Guyana	GUY	2013	AMR	UMIC	83.54	0.30	83.24	98.21	59.50	3.85	55.65	66.85
Guyana	GUY	2014	AMR	UMIC	84.56	0.37	84.18	98.23	61.24	3.96	57.28	68.04
Guyana	GUY	2015	AMR	UMIC	83.21	0.28	82.94	98.20	59.02	3.81	55.21	66.56
Guyana	GUY	2016	AMR	UMIC	83.16	0.28	82.88	98.20	58.94	3.81	55.13	66.52
Guyana	GUY	2017	AMR	UMIC	83.04	0.27	82.77	98.20	58.78	3.80	54.98	66.43
Guyana	GUY	2018	AMR	UMIC	82.42	0.25	82.16	98.19	57.98	3.75	54.23	66.00
Guyana	GUY	2019	AMR	UMIC	82.34	0.25	82.09	98.19	57.88	3.74	54.14	65.96
Honduras	HND	2010	AMR	LMIC	85.02	0.42	84.60	99.00	62.21	4.02	58.19	68.78
Honduras	HND	2011	AMR	LMIC	84.61	0.38	84.23	98.99	61.35	3.97	57.38	68.12
Honduras	HND	2012	AMR	LMIC	84.87	0.40	84.46	99.00	61.87	4.00	57.87	68.52
Honduras	HND	2013	AMR	LMIC	85.68	0.52	85.16	99.02	63.86	4.13	59.73	70.14
Honduras	HND	2014	AMR	LMIC	83.22	0.28	82.94	98.96	59.03	3.82	55.21	66.57
Honduras	HND	2015	AMR	LMIC	84.03	0.33	83.70	98.98	60.28	3.90	56.39	67.37
Honduras	HND	2016	AMR	LMIC	83.39	0.29	83.10	98.96	59.27	3.83	55.44	66.71
Honduras	HND	2017	AMR	LMIC	82.24	0.25	81.99	98.94	57.77	3.73	54.04	65.90
Honduras	HND	2018	AMR	LMIC	83.90	0.32	83.58	98.97	60.06	3.88	56.18	67.22
Honduras	HND	2019	AMR	LMIC	79.97	0.25	79.72	98.91	55.53	3.59	51.94	65.15
Haiti	HTI	2010	AMR	LIC	72.14	2.20	69.94	61.02	66.52	4.30	62.22	88.97
Haiti	HTI	2011	AMR	LIC	73.82	0.75	73.07	72.21	57.87	3.74	54.13	74.08
Haiti	HTI	2012	AMR	LIC	83.76	0.74	83.03	88.62	67.53	4.36	63.16	76.07
Haiti	HTI	2013	AMR	LIC	86.93	0.77	86.16	95.15	69.88	4.52	65.36	75.86
Haiti	HTI	2014	AMR	LIC	84.17	1.09	83.08	86.52	71.11	4.60	66.51	80.05
Haiti	HTI	2015	AMR	LIC	85.75	0.77	84.98	92.95	68.82	4.45	64.37	75.75
Haiti	HTI	2016	AMR	LIC	82.94	0.73	82.21	86.49	67.31	4.35	62.96	76.58
Haiti	HTI	2017	AMR	LIC	82.13	0.78	81.35	86.48	65.49	4.23	61.26	75.29
Haiti	HTI	2018	AMR	LIC	76.71	0.71	76.00	74.21	63.01	4.07	58.94	77.55
Haiti	HTI	2019	AMR	LIC	73.81	0.74	73.07	72.21	57.80	3.74	54.07	74.00
Indonesia	IDN	2010	SEA	UMIC	40.55	0.63	39.91	36.86	34.71	2.24	32.46	81.33
Indonesia	IDN	2011	SEA	UMIC	43.06	1.04	42.02	40.18	37.90	2.45	35.45	84.37
Indonesia	IDN	2012	SEA	UMIC	44.14	0.99	43.16	41.87	38.65	2.50	36.15	83.76
Indonesia	IDN	2013	SEA	UMIC	44.91	1.28	43.63	41.88	40.79	2.64	38.15	87.45
Indonesia	IDN	2014	SEA	UMIC	43.09	1.09	42.00	40.18	38.07	2.46	35.61	84.78
Indonesia	IDN	2015	SEA	UMIC	44.31	1.27	43.04	41.88	39.59	2.56	37.03	86.03
Indonesia	IDN	2016	SEA	UMIC	48.21	0.60	47.62	48.91	40.60	2.62	37.97	79.75
Indonesia	IDN	2017	SEA	UMIC	58.53	0.88	57.65	66.20	49.99	3.23	46.76	81.10
Indonesia	IDN	2018	SEA	UMIC	75.65	1.42	74.23	99.11	65.46	4.23	61.22	82.48
Indonesia	IDN	2019	SEA	UMIC	75.95	1.88	74.07	99.12	67.02	4.33	62.69	84.63
India	IND	2010	SEA	LMIC	51.00	1.51	49.49	24.40	46.75	3.02	43.73	88.35
India	IND	2011	SEA	LMIC	50.44	1.44	49.00	24.39	45.56	2.94	42.61	86.96
India	IND	2012	SEA	LMIC	49.22	1.41	47.82	23.29	44.38	2.87	41.52	86.82
India	IND	2013	SEA	LMIC	48.65	1.06	47.60	23.28	42.50	2.75	39.75	83.52
India	IND	2014	SEA	LMIC	63.42	1.46	61.95	39.66	55.64	3.60	52.05	84.01
India	IND	2015	SEA	LMIC	65.88	0.68	65.19	44.04	54.74	3.54	51.21	78.54
India	IND	2016	SEA	LMIC	70.42	0.76	69.66	50.26	58.70	3.79	54.91	78.82
India	IND	2017	SEA	LMIC	66.15	0.58	65.57	45.54	53.35	3.45	49.91	76.11
India	IND	2018	SEA	LMIC	79.40	0.87	78.53	63.96	66.28	4.28	62.00	78.95
India	IND	2019	SEA	LMIC	92.16	1.28	90.88	85.53	78.24	5.06	73.18	80.52

Iran (Islamic Republic of)	IRN	2010	EMR	UMIC	87.76	0.81	86.95	99.82	70.17	4.54	65.64	75.49
Iran (Islamic Republic of)	IRN	2011	EMR	UMIC	90.14	1.44	88.69	99.86	77.27	4.99	72.27	81.49
Iran (Islamic Republic of)	IRN	2012	EMR	UMIC	77.65	0.33	77.33	99.63	53.83	3.48	50.35	65.12
Iran (Islamic Republic of)	IRN	2013	EMR	UMIC	78.74	0.28	78.47	99.64	54.58	3.53	51.05	65.06
Iran (Islamic Republic of)	IRN	2014	EMR	UMIC	83.65	0.30	83.35	99.72	59.66	3.86	55.81	66.96
Iran (Islamic Republic of)	IRN	2015	EMR	UMIC	84.85	0.40	84.45	99.75	61.84	4.00	57.85	68.50
Iran (Islamic Republic of)	IRN	2016	EMR	UMIC	84.67	0.38	84.29	99.74	61.46	3.97	57.49	68.21
Iran (Islamic Republic of)	IRN	2017	EMR	UMIC	84.83	0.40	84.43	99.75	61.80	3.99	57.80	68.46
Iran (Islamic Republic of)	IRN	2018	EMR	UMIC	85.47	0.49	84.98	99.77	63.27	4.09	59.18	69.64
Iran (Islamic Republic of)	IRN	2019	EMR	UMIC	87.62	0.83	86.79	99.83	69.87	4.52	65.36	75.31
Iraq	IRQ	2010	EMR	UMIC	72.44	0.22	72.22	98.12	50.38	3.26	47.12	65.25
Iraq	IRQ	2011	EMR	UMIC	64.36	0.19	64.16	80.53	44.75	2.89	41.86	65.24
Iraq	IRQ	2012	EMR	UMIC	60.00	0.21	59.78	74.28	41.58	2.69	38.89	65.06
Iraq	IRQ	2013	EMR	UMIC	57.09	0.20	56.89	68.25	39.57	2.56	37.01	65.06
Iraq	IRQ	2014	EMR	UMIC	54.63	0.18	54.45	62.44	37.89	2.45	35.44	65.09
Iraq	IRQ	2015	EMR	UMIC	52.53	0.17	52.35	58.68	36.42	2.35	34.07	65.08
Iraq	IRQ	2016	EMR	UMIC	45.90	0.14	45.75	46.27	31.86	2.06	29.80	65.13
Iraq	IRQ	2017	EMR	UMIC	48.38	0.14	48.24	49.70	33.67	2.18	31.49	65.28
Iraq	IRQ	2018	EMR	UMIC	44.14	0.13	44.00	42.94	30.67	1.98	28.69	65.20
Iraq	IRQ	2019	EMR	UMIC	41.00	0.13	40.88	38.14	28.47	1.84	26.63	65.16
Jordan	JOR	2010	EMR	UMIC	72.73	1.11	71.61	98.84	51.10	3.30	47.80	66.75
Jordan	JOR	2011	EMR	UMIC	72.73	1.07	71.65	98.84	51.06	3.30	47.76	66.65
Jordan	JOR	2012	EMR	UMIC	72.73	0.98	71.75	98.84	50.92	3.29	47.63	66.39
Jordan	JOR	2013	EMR	UMIC	72.73	0.98	71.75	98.84	50.93	3.29	47.64	66.39
Jordan	JOR	2014	EMR	UMIC	73.84	0.84	73.00	98.86	51.75	3.34	48.40	66.30
Jordan	JOR	2015	EMR	UMIC	75.37	0.51	74.85	98.87	52.51	3.39	49.11	65.61
Jordan	JOR	2016	EMR	UMIC	75.43	0.51	74.92	98.87	52.54	3.40	49.14	65.59
Jordan	JOR	2017	EMR	UMIC	78.21	0.30	77.91	98.89	54.20	3.50	50.70	65.07
Jordan	JOR	2018	EMR	UMIC	75.84	0.46	75.38	98.87	52.76	3.41	49.35	65.47
Jordan	JOR	2019	EMR	UMIC	73.92	0.82	73.09	98.85	51.78	3.35	48.44	66.26
Kazakhstan	KAZ	2010	EUR	UMIC	93.72	0.72	93.00	62.32	65.38	4.23	61.15	65.76
Kazakhstan	KAZ	2011	EUR	UMIC	100.00	1.75	98.25	95.31	70.46	4.55	65.91	67.08
Kazakhstan	KAZ	2012	EUR	UMIC	80.00	0.63	79.37	35.18	54.50	3.52	50.98	64.23
Kazakhstan	KAZ	2013	EUR	UMIC	90.91	0.53	90.38	62.21	60.27	3.90	56.38	62.38
Kazakhstan	KAZ	2014	EUR	UMIC	88.18	0.52	87.67	54.30	58.43	3.78	54.66	62.35
Kazakhstan	KAZ	2015	EUR	UMIC	90.00	0.47	89.53	59.46	58.57	3.79	54.78	61.19
Kazakhstan	KAZ	2016	EUR	UMIC	85.45	0.59	84.87	47.21	57.55	3.72	53.83	63.43
Kazakhstan	KAZ	2017	EUR	UMIC	90.91	0.61	90.30	62.23	61.07	3.95	57.12	63.25
Kazakhstan	KAZ	2018	EUR	UMIC	90.91	0.60	90.31	62.23	61.02	3.94	57.07	63.20
Kazakhstan	KAZ	2019	EUR	UMIC	89.09	0.66	88.43	56.88	60.40	3.90	56.50	63.89
Kenya	KEN	2010	AFR	LMIC	53.07	1.86	51.21	58.29	50.29	3.25	47.04	91.85
Kenya	KEN	2011	AFR	LMIC	51.23	1.48	49.75	58.27	45.93	2.97	42.96	86.35
Kenya	KEN	2012	AFR	LMIC	52.16	1.54	50.62	58.27	47.81	3.09	44.72	88.35
Kenya	KEN	2013	AFR	LMIC	51.09	1.41	49.69	58.26	45.49	2.94	42.55	85.64
Kenya	KEN	2014	AFR	LMIC	50.35	0.57	49.79	58.24	42.11	2.72	39.39	79.12
Kenya	KEN	2015	AFR	LMIC	49.71	0.43	49.27	58.23	40.25	2.60	37.64	76.40
Kenya	KEN	2016	AFR	LMIC	49.36	0.46	48.91	58.23	39.47	2.55	36.92	75.48

Kenya	KEN	2017	AFR	LMIC	57.99	0.58	57.41	70.81	48.03	3.10	44.92	78.24
Kenya	KEN	2018	AFR	LMIC	70.37	0.74	69.63	91.77	58.53	3.78	54.75	78.63
Kenya	KEN	2019	AFR	LMIC	65.10	0.64	64.46	85.88	50.89	3.29	47.60	73.84
Kyrgyzstan	KGZ	2010	EUR	LMIC	85.60	0.30	85.29	95.09	59.33	3.83	55.49	65.06
Kyrgyzstan	KGZ	2011	EUR	LMIC	86.13	0.29	85.85	95.09	59.72	3.86	55.86	65.07
Kyrgyzstan	KGZ	2012	EUR	LMIC	81.05	0.73	80.32	95.04	56.63	3.66	52.97	65.95
Kyrgyzstan	KGZ	2013	EUR	LMIC	81.75	0.59	81.16	95.05	56.99	3.68	53.30	65.68
Kyrgyzstan	KGZ	2014	EUR	LMIC	80.64	0.83	79.82	95.04	56.44	3.65	52.79	66.14
Kyrgyzstan	KGZ	2015	EUR	LMIC	82.47	0.50	81.98	95.06	57.37	3.71	53.67	65.46
Kyrgyzstan	KGZ	2016	EUR	LMIC	83.96	0.38	83.58	95.07	58.24	3.76	54.47	65.17
Kyrgyzstan	KGZ	2017	EUR	LMIC	81.12	0.71	80.41	95.04	56.67	3.66	53.01	65.92
Kyrgyzstan	KGZ	2018	EUR	LMIC	89.02	0.27	88.75	95.13	62.35	4.03	58.32	65.71
Kyrgyzstan	KGZ	2019	EUR	LMIC	90.66	0.31	90.35	95.16	64.43	4.16	60.26	66.70
Cambodia	KHM	2010	WPR	LMIC	70.40	0.63	69.78	56.20	54.59	3.53	51.06	73.18
Cambodia	KHM	2011	WPR	LMIC	67.20	0.62	66.58	51.66	52.26	3.38	48.88	73.42
Cambodia	KHM	2012	WPR	LMIC	71.51	0.65	70.86	57.76	55.52	3.59	51.93	73.29
Cambodia	KHM	2013	WPR	LMIC	68.29	0.63	67.66	53.16	53.13	3.43	49.70	73.45
Cambodia	KHM	2014	WPR	LMIC	80.09	0.68	79.41	70.85	61.88	4.00	57.88	72.90
Cambodia	KHM	2015	WPR	LMIC	66.62	0.45	66.17	51.64	50.12	3.24	46.88	70.84
Cambodia	KHM	2016	WPR	LMIC	65.50	0.43	65.07	50.17	49.18	3.18	46.00	70.69
Cambodia	KHM	2017	WPR	LMIC	69.79	0.46	69.33	56.19	52.38	3.39	49.00	70.67
Cambodia	KHM	2018	WPR	LMIC	61.17	0.39	60.77	44.44	45.83	2.96	42.87	70.54
Cambodia	KHM	2019	WPR	LMIC	67.68	0.45	67.23	53.14	50.89	3.29	47.60	70.80
Kiribati	KIR	2010	WPR	LMIC	84.22	0.35	83.88	97.98	60.62	3.92	56.70	67.60
Kiribati	KIR	2011	WPR	LMIC	82.95	0.27	82.69	97.96	58.66	3.79	54.87	66.36
Kiribati	KIR	2012	WPR	LMIC	83.45	0.29	83.16	97.96	59.36	3.84	55.52	66.77
Kiribati	KIR	2013	WPR	LMIC	84.93	0.41	84.52	98.00	62.00	4.01	58.00	68.62
Kiribati	KIR	2014	WPR	LMIC	82.03	0.25	81.78	97.94	57.52	3.72	53.81	65.79
Kiribati	KIR	2015	WPR	LMIC	90.40	1.80	88.61	98.14	78.52	5.08	73.44	82.89
Kiribati	KIR	2016	WPR	LMIC	89.31	0.92	88.39	98.11	74.19	4.80	69.40	78.51
Kiribati	KIR	2017	WPR	LMIC	83.25	0.28	82.97	97.96	59.07	3.82	55.25	66.59
Kiribati	KIR	2018	WPR	LMIC	84.43	0.36	84.07	97.99	61.00	3.94	57.05	67.87
Kiribati	KIR	2019	WPR	LMIC	85.36	0.47	84.89	98.01	63.00	4.07	58.93	69.42
Lao People's Democratic Republic	LAO	2010	WPR	LMIC	30.78	0.15	30.63	35.15	22.47	1.45	21.01	68.60
Lao People's Democratic Republic	LAO	2011	WPR	LMIC	34.15	0.19	33.96	40.33	25.23	1.63	23.60	69.47
Lao People's Democratic Republic	LAO	2012	WPR	LMIC	32.53	0.13	32.41	38.57	23.31	1.51	21.81	67.29
Lao People's Democratic Republic	LAO	2013	WPR	LMIC	33.80	0.15	33.66	40.32	24.46	1.58	22.88	67.97
Lao People's Democratic Republic	LAO	2014	WPR	LMIC	37.43	0.22	37.21	45.71	27.79	1.80	25.99	69.85
Lao People's Democratic Republic	LAO	2015	WPR	LMIC	39.50	0.22	39.28	49.40	29.19	1.89	27.30	69.51
Lao People's Democratic Republic	LAO	2016	WPR	LMIC	44.93	0.26	44.66	58.97	33.37	2.16	31.21	69.89
Lao People's Democratic Republic	LAO	2017	WPR	LMIC	52.92	0.41	52.50	73.15	40.52	2.62	37.90	72.18
Lao People's Democratic Republic	LAO	2018	WPR	LMIC	61.49	0.46	61.03	90.34	46.88	3.03	43.85	71.86
Lao People's Democratic Republic	LAO	2019	WPR	LMIC	65.86	0.51	65.35	99.32	50.40	3.26	47.14	72.13
Lebanon	LBN	2010	EMR	UMIC	89.41	0.27	89.14	98.40	62.79	4.06	58.73	65.89
Lebanon	LBN	2011	EMR	UMIC	89.86	0.28	89.58	98.41	63.34	4.09	59.24	66.13
Lebanon	LBN	2012	EMR	UMIC	89.61	0.27	89.33	98.41	63.03	4.07	58.95	65.99
Lebanon	LBN	2013	EMR	UMIC	79.09	0.61	78.48	98.25	53.79	3.48	50.31	64.11

Lebanon	LBN	2014	EMR	UMIC	83.12	0.44	82.68	98.32	57.73	3.73	54.00	65.32
Lebanon	LBN	2015	EMR	UMIC	81.98	0.56	81.43	98.31	57.11	3.69	53.42	65.60
Lebanon	LBN	2016	EMR	UMIC	86.22	0.29	85.93	98.36	59.79	3.86	55.92	65.08
Lebanon	LBN	2017	EMR	UMIC	85.76	0.30	85.46	98.37	59.44	3.84	55.60	65.06
Lebanon	LBN	2018	EMR	UMIC	85.95	0.29	85.66	98.35	59.58	3.85	55.73	65.06
Lebanon	LBN	2019	EMR	UMIC	87.41	0.26	87.15	98.37	60.77	3.93	56.84	65.22
Liberia	LBR	2010	AFR	LIC	64.95	0.75	64.19	69.11	54.45	3.52	50.93	79.34
Liberia	LBR	2011	AFR	LIC	74.84	1.89	72.95	85.26	66.14	4.28	61.87	84.81
Liberia	LBR	2012	AFR	LIC	70.16	0.65	69.51	81.05	56.04	3.62	52.42	75.41
Liberia	LBR	2013	AFR	LIC	67.84	2.64	65.19	67.25	65.91	4.26	61.65	94.57
Liberia	LBR	2014	AFR	LIC	41.95	1.05	40.90	33.32	37.04	2.39	34.65	84.72
Liberia	LBR	2015	AFR	LIC	49.19	1.59	47.60	40.95	45.93	2.97	42.96	90.25
Liberia	LBR	2016	AFR	LIC	58.56	1.90	56.66	54.35	54.67	3.53	51.13	90.24
Liberia	LBR	2017	AFR	LIC	57.35	0.49	56.87	59.66	44.28	2.86	41.42	72.84
Liberia	LBR	2018	AFR	LIC	61.71	1.90	59.82	59.74	56.99	3.68	53.31	89.11
Liberia	LBR	2019	AFR	LIC	60.80	0.93	59.87	61.55	51.99	3.36	48.63	81.23
Saint Lucia	LCA	2010	AMR	UMIC	79.09	0.94	78.15	99.08	55.06	3.56	51.50	65.90
Saint Lucia	LCA	2011	AMR	UMIC	79.09	0.75	78.34	99.07	54.48	3.52	50.96	65.05
Saint Lucia	LCA	2012	AMR	UMIC	79.09	0.87	78.22	99.07	54.90	3.55	51.35	65.65
Saint Lucia	LCA	2013	AMR	UMIC	79.09	0.55	78.54	99.04	53.37	3.45	49.92	63.57
Saint Lucia	LCA	2014	AMR	UMIC	79.09	0.44	78.65	99.02	52.15	3.37	48.78	62.02
Saint Lucia	LCA	2015	AMR	UMIC	79.09	0.80	78.29	99.07	54.67	3.53	51.14	65.32
Saint Lucia	LCA	2016	AMR	UMIC	79.09	0.42	78.67	99.01	51.65	3.34	48.31	61.40
Saint Lucia	LCA	2017	AMR	UMIC	79.09	0.44	78.65	99.02	52.12	3.37	48.75	61.98
Saint Lucia	LCA	2018	AMR	UMIC	79.09	0.44	78.65	99.02	52.12	3.37	48.75	61.99
Saint Lucia	LCA	2019	AMR	UMIC	79.09	0.61	78.48	99.05	53.80	3.48	50.33	64.13
Sri Lanka	LKA	2010	SEA	LMIC	78.57	0.38	78.19	96.34	57.38	3.71	53.67	68.64
Sri Lanka	LKA	2011	SEA	LMIC	79.91	0.42	79.49	98.63	58.79	3.80	54.99	69.18
Sri Lanka	LKA	2012	SEA	LMIC	71.18	0.35	70.83	80.78	52.04	3.36	48.68	68.72
Sri Lanka	LKA	2013	SEA	LMIC	72.44	0.38	72.06	82.93	53.27	3.44	49.83	69.15
Sri Lanka	LKA	2014	SEA	LMIC	73.46	0.38	73.08	85.11	53.94	3.49	50.46	69.04
Sri Lanka	LKA	2015	SEA	LMIC	71.90	0.32	71.58	82.92	52.11	3.37	48.74	68.09
Sri Lanka	LKA	2016	SEA	LMIC	66.46	0.22	66.24	74.45	47.06	3.04	44.02	66.45
Sri Lanka	LKA	2017	SEA	LMIC	63.74	0.23	63.51	68.38	45.42	2.94	42.49	66.90
Sri Lanka	LKA	2018	SEA	LMIC	67.62	0.30	67.32	74.47	48.94	3.16	45.77	67.99
Sri Lanka	LKA	2019	SEA	LMIC	65.06	0.35	64.71	68.41	47.97	3.10	44.87	69.35
Lesotho	LSO	2010	AFR	LMIC	54.94	0.68	54.26	64.94	46.25	2.99	43.26	79.73
Lesotho	LSO	2011	AFR	LMIC	59.76	1.71	58.06	69.59	54.14	3.50	50.64	87.23
Lesotho	LSO	2012	AFR	LMIC	55.96	0.51	55.44	68.00	44.76	2.89	41.86	75.51
Lesotho	LSO	2013	AFR	LMIC	56.09	0.71	55.38	66.48	47.30	3.06	44.24	79.89
Lesotho	LSO	2014	AFR	LMIC	57.98	1.69	56.29	68.04	51.90	3.35	48.54	86.24
Lesotho	LSO	2015	AFR	LMIC	55.96	2.19	53.77	61.93	54.44	3.52	50.92	94.72
Lesotho	LSO	2016	AFR	LMIC	54.65	0.55	54.11	64.94	45.28	2.93	42.35	78.28
Lesotho	LSO	2017	AFR	LMIC	53.89	0.39	53.50	66.44	40.97	2.65	38.32	71.63
Lesotho	LSO	2018	AFR	LMIC	55.99	0.51	55.48	68.00	44.83	2.90	41.93	75.58
Lesotho	LSO	2019	AFR	LMIC	54.33	0.29	54.04	67.97	39.95	2.58	37.37	69.14
Morocco	MAR	2010	EMR	LMIC	96.44	0.86	95.59	97.25	78.68	5.09	73.59	76.99
Morocco	MAR	2011	EMR	LMIC	94.54	1.04	93.50	97.22	74.51	4.82	69.70	74.54

Morocco	MAR	2012	EMR	LMIC	94.41	0.94	93.48	97.21	73.88	4.78	69.11	73.93
Morocco	MAR	2013	EMR	LMIC	94.12	0.79	93.33	97.20	72.59	4.69	67.89	72.75
Morocco	MAR	2014	EMR	LMIC	94.18	0.81	93.37	97.20	72.84	4.71	68.14	72.98
Morocco	MAR	2015	EMR	LMIC	94.60	1.05	93.55	97.22	74.69	4.83	69.86	74.68
Morocco	MAR	2016	EMR	LMIC	95.54	0.87	94.67	97.23	76.52	4.95	71.58	75.61
Morocco	MAR	2017	EMR	LMIC	94.74	1.02	93.72	97.22	74.93	4.84	70.09	74.79
Morocco	MAR	2018	EMR	LMIC	95.19	0.92	94.27	97.23	75.80	4.90	70.90	75.21
Morocco	MAR	2019	EMR	LMIC	94.47	0.98	93.49	97.21	74.14	4.79	69.35	74.18
Republic of Moldova	MDA	2010	EUR	LMIC	86.01	0.29	85.72	88.19	59.63	3.85	55.78	65.07
Republic of Moldova	MDA	2011	EUR	LMIC	85.29	0.32	84.98	88.18	59.11	3.82	55.29	65.06
Republic of Moldova	MDA	2012	EUR	LMIC	84.57	0.35	84.22	88.18	58.62	3.79	54.83	65.11
Republic of Moldova	MDA	2013	EUR	LMIC	84.30	0.36	83.94	88.17	58.45	3.78	54.67	65.13
Republic of Moldova	MDA	2014	EUR	LMIC	79.09	1.00	78.09	88.12	55.22	3.57	51.65	66.14
Republic of Moldova	MDA	2015	EUR	LMIC	80.95	0.75	80.20	88.14	56.58	3.66	52.93	66.00
Republic of Moldova	MDA	2016	EUR	LMIC	79.09	1.09	78.00	88.12	55.42	3.58	51.83	66.45
Republic of Moldova	MDA	2017	EUR	LMIC	79.09	0.73	78.37	88.10	54.38	3.51	50.86	64.90
Republic of Moldova	MDA	2018	EUR	LMIC	79.09	0.71	78.38	88.10	54.32	3.51	50.81	64.83
Republic of Moldova	MDA	2019	EUR	LMIC	79.09	0.81	78.29	88.11	54.70	3.54	51.16	65.35
Madagascar	MDG	2010	AFR	LIC	58.32	3.29	55.03	73.06	62.33	4.03	58.30	105.96
Madagascar	MDG	2011	AFR	LIC	56.32	0.89	55.44	76.97	48.24	3.12	45.12	81.40
Madagascar	MDG	2012	AFR	LIC	55.41	1.15	54.25	74.98	48.27	3.12	45.15	83.21
Madagascar	MDG	2013	AFR	LIC	59.22	2.18	57.04	77.02	56.75	3.67	53.08	93.06
Madagascar	MDG	2014	AFR	LIC	61.71	2.33	59.39	81.05	59.47	3.84	55.63	93.67
Madagascar	MDG	2015	AFR	LIC	61.73	2.33	59.40	81.05	59.54	3.85	55.69	93.76
Madagascar	MDG	2016	AFR	LIC	58.39	1.84	56.55	77.00	54.20	3.50	50.70	89.65
Madagascar	MDG	2017	AFR	LIC	62.57	2.22	60.35	83.08	59.45	3.84	55.61	92.14
Madagascar	MDG	2018	AFR	LIC	65.13	2.39	62.74	87.19	62.37	4.03	58.34	93.00
Madagascar	MDG	2019	AFR	LIC	70.51	2.89	67.61	95.55	69.47	4.49	64.98	96.11
Maldives	MDV	2010	SEA	UMIC	72.73	0.29	72.44	99.68	41.47	2.68	38.79	53.55
Maldives	MDV	2011	SEA	UMIC	73.50	0.96	72.55	99.85	51.59	3.33	48.26	66.52
Maldives	MDV	2012	SEA	UMIC	81.72	0.24	81.47	99.90	57.18	3.70	53.48	65.64
Maldives	MDV	2013	SEA	UMIC	82.67	0.26	82.41	99.91	58.29	3.77	54.52	66.16
Maldives	MDV	2014	SEA	UMIC	78.93	0.27	78.66	99.89	54.71	3.54	51.17	65.06
Maldives	MDV	2015	SEA	UMIC	82.19	0.25	81.94	99.91	57.71	3.73	53.98	65.87
Maldives	MDV	2016	SEA	UMIC	81.63	0.24	81.39	99.96	57.09	3.69	53.40	65.61
Maldives	MDV	2017	SEA	UMIC	78.81	0.28	78.53	99.96	54.62	3.53	51.09	65.06
Maldives	MDV	2018	SEA	UMIC	78.36	0.29	78.07	99.97	54.31	3.51	50.80	65.07
Maldives	MDV	2019	SEA	UMIC	78.29	0.30	78.00	99.98	54.26	3.51	50.75	65.07
Mexico	MEX	2010	AMR	UMIC	82.50	0.25	82.25	99.21	58.08	3.75	54.33	66.05
Mexico	MEX	2011	AMR	UMIC	82.75	0.26	82.49	99.22	58.40	3.77	54.62	66.22
Mexico	MEX	2012	AMR	UMIC	82.53	0.25	82.27	99.21	58.12	3.76	54.36	66.07
Mexico	MEX	2013	AMR	UMIC	81.90	0.24	81.65	99.20	57.38	3.71	53.67	65.73
Mexico	MEX	2014	AMR	UMIC	80.64	0.24	80.40	99.18	56.11	3.63	52.49	65.28
Mexico	MEX	2015	AMR	UMIC	82.32	0.25	82.06	99.21	57.86	3.74	54.12	65.94
Mexico	MEX	2016	AMR	UMIC	82.84	0.26	82.58	99.22	58.51	3.78	54.73	66.28
Mexico	MEX	2017	AMR	UMIC	82.72	0.26	82.46	99.22	58.36	3.77	54.59	66.19
Mexico	MEX	2018	AMR	UMIC	83.02	0.27	82.75	99.22	58.75	3.80	54.95	66.41
Mexico	MEX	2019	AMR	UMIC	82.75	0.26	82.49	99.22	58.40	3.77	54.62	66.22

Marshall Islands	MHL	2010	WPR	UMIC	72.73	0.38	72.34	99.70	47.45	3.07	44.38	61.35
Marshall Islands	MHL	2011	WPR	UMIC	72.73	0.31	72.42	99.65	44.05	2.85	41.21	56.90
Marshall Islands	MHL	2012	WPR	UMIC	72.73	0.72	72.01	99.76	50.22	3.25	46.97	65.23
Marshall Islands	MHL	2013	WPR	UMIC	73.13	1.10	72.03	99.77	51.42	3.32	48.10	66.77
Marshall Islands	MHL	2014	WPR	UMIC	73.11	1.10	72.01	99.77	51.41	3.32	48.09	66.78
Marshall Islands	MHL	2015	WPR	UMIC	72.73	1.15	71.58	99.81	51.14	3.31	47.84	66.83
Marshall Islands	MHL	2016	WPR	UMIC	79.63	0.26	79.38	99.83	55.25	3.57	51.68	65.11
Marshall Islands	MHL	2017	WPR	UMIC	72.73	0.79	71.94	99.76	50.44	3.26	47.18	65.59
Marshall Islands	MHL	2018	WPR	UMIC	75.99	0.44	75.55	99.78	52.84	3.42	49.43	65.42
Marshall Islands	MHL	2019	WPR	UMIC	76.26	0.42	75.84	99.79	52.99	3.43	49.57	65.36
North Macedonia	MKD	2010	EUR	UMIC	78.04	0.31	77.74	98.89	54.09	3.50	50.59	65.08
North Macedonia	MKD	2011	EUR	UMIC	83.61	0.30	83.31	98.96	59.60	3.85	55.75	66.92
North Macedonia	MKD	2012	EUR	UMIC	75.64	0.48	75.16	98.86	52.65	3.40	49.25	65.52
North Macedonia	MKD	2013	EUR	UMIC	74.13	0.77	73.36	98.85	51.88	3.35	48.53	66.15
North Macedonia	MKD	2014	EUR	UMIC	72.73	0.76	71.97	98.82	50.35	3.25	47.10	65.44
North Macedonia	MKD	2015	EUR	UMIC	72.73	0.57	72.16	98.81	49.51	3.20	46.31	64.17
North Macedonia	MKD	2016	EUR	UMIC	72.73	0.70	72.02	98.82	50.16	3.24	46.92	65.15
North Macedonia	MKD	2017	EUR	UMIC	72.73	0.65	72.08	98.82	49.91	3.23	46.69	64.77
North Macedonia	MKD	2018	EUR	UMIC	72.73	0.50	72.22	98.80	49.02	3.17	45.85	63.48
North Macedonia	MKD	2019	EUR	UMIC	72.73	0.45	72.27	98.79	48.53	3.14	45.39	62.81
Mali	MLI	2010	AFR	LIC	66.23	2.40	63.83	72.58	63.22	4.09	59.13	92.64
Mali	MLI	2011	AFR	LIC	66.64	2.12	64.52	74.54	61.99	4.01	57.98	89.87
Mali	MLI	2012	AFR	LIC	64.66	1.87	62.79	72.56	58.93	3.81	55.12	87.80
Mali	MLI	2013	AFR	LIC	68.27	2.00	66.27	78.53	62.41	4.03	58.38	88.10
Mali	MLI	2014	AFR	LIC	70.17	2.24	67.93	80.56	65.31	4.22	61.09	89.93
Mali	MLI	2015	AFR	LIC	77.16	2.03	75.12	97.35	68.43	4.42	64.01	85.20
Mali	MLI	2016	AFR	LIC	72.38	0.37	72.00	97.23	53.12	3.43	49.69	69.00
Mali	MLI	2017	AFR	LIC	67.29	0.38	66.91	86.61	49.79	3.22	46.57	69.60
Mali	MLI	2018	AFR	LIC	73.04	0.48	72.56	97.25	54.90	3.55	51.35	70.77
Mali	MLI	2019	AFR	LIC	72.83	0.82	72.01	95.12	57.47	3.71	53.75	74.65
Myanmar	MMR	2010	SEA	LMIC	63.23	3.63	59.60	34.17	67.98	4.39	63.59	106.69
Myanmar	MMR	2011	SEA	LMIC	66.47	2.51	63.96	39.25	64.07	4.14	59.93	93.69
Myanmar	MMR	2012	SEA	LMIC	73.50	3.49	70.00	46.11	74.90	4.84	70.06	100.08
Myanmar	MMR	2013	SEA	LMIC	70.25	2.27	67.98	44.68	65.54	4.24	61.30	90.17
Myanmar	MMR	2014	SEA	LMIC	74.68	2.34	72.34	50.44	69.22	4.47	64.75	89.50
Myanmar	MMR	2015	SEA	LMIC	78.43	2.52	75.90	54.98	73.13	4.73	68.40	90.12
Myanmar	MMR	2016	SEA	LMIC	78.61	1.05	77.56	59.65	66.54	4.30	62.24	80.24
Myanmar	MMR	2017	SEA	LMIC	78.11	1.76	76.35	58.07	68.41	4.42	63.99	83.81
Myanmar	MMR	2018	SEA	LMIC	85.97	1.84	84.13	69.63	75.03	4.85	70.18	83.42
Myanmar	MMR	2019	SEA	LMIC	86.92	1.60	85.32	71.36	75.11	4.85	70.25	82.34
Montenegro	MNE	2010	EUR	UMIC	79.09	0.80	78.29	99.04	54.68	3.53	51.15	65.33
Montenegro	MNE	2011	EUR	UMIC	79.09	0.62	78.47	99.03	53.87	3.48	50.39	64.22
Montenegro	MNE	2012	EUR	UMIC	79.09	0.91	78.18	99.05	54.99	3.55	51.44	65.79
Montenegro	MNE	2013	EUR	UMIC	84.89	0.33	84.56	99.12	58.83	3.80	55.03	65.08
Montenegro	MNE	2014	EUR	UMIC	79.09	0.52	78.57	99.01	53.07	3.43	49.64	63.18
Montenegro	MNE	2015	EUR	UMIC	79.09	0.45	78.64	98.99	52.29	3.38	48.91	62.20
Montenegro	MNE	2016	EUR	UMIC	79.09	0.70	78.39	99.03	54.28	3.51	50.77	64.77
Montenegro	MNE	2017	EUR	UMIC	79.09	0.62	78.47	99.03	53.85	3.48	50.37	64.19

Montenegro	MNE	2018	EUR	UMIC	79.09	0.69	78.40	99.03	54.22	3.50	50.72	64.69
Montenegro	MNE	2019	EUR	UMIC	79.09	0.68	78.41	99.03	54.18	3.50	50.68	64.64
Mongolia	MNG	2010	WPR	LMIC	39.86	0.15	39.71	49.16	28.55	1.85	26.71	67.25
Mongolia	MNG	2011	WPR	LMIC	37.49	0.13	37.36	46.36	26.62	1.72	24.90	66.64
Mongolia	MNG	2012	WPR	LMIC	35.01	0.11	34.90	43.58	24.62	1.59	23.03	65.97
Mongolia	MNG	2013	WPR	LMIC	36.20	0.11	36.09	44.97	25.55	1.65	23.90	66.21
Mongolia	MNG	2014	WPR	LMIC	36.52	0.11	36.41	46.35	25.47	1.65	23.82	65.43
Mongolia	MNG	2015	WPR	LMIC	36.72	0.11	36.61	46.35	25.68	1.66	24.02	65.60
Mongolia	MNG	2016	WPR	LMIC	34.98	0.11	34.88	43.58	24.59	1.59	23.00	65.94
Mongolia	MNG	2017	WPR	LMIC	32.62	0.10	32.52	40.81	22.80	1.47	21.33	65.58
Mongolia	MNG	2018	WPR	LMIC	28.94	0.09	28.85	36.68	20.09	1.30	18.79	65.13
Mongolia	MNG	2019	WPR	LMIC	30.99	0.10	30.89	39.42	21.52	1.39	20.12	65.15
Mozambique	MOZ	2010	AFR	LIC	59.48	1.70	57.78	24.13	53.58	3.46	50.12	86.74
Mozambique	MOZ	2011	AFR	LIC	56.51	0.50	56.01	22.99	46.05	2.98	43.07	76.90
Mozambique	MOZ	2012	AFR	LIC	60.20	1.71	58.50	25.28	53.74	3.47	50.26	85.92
Mozambique	MOZ	2013	AFR	LIC	67.78	2.15	65.63	31.55	63.00	4.07	58.93	89.78
Mozambique	MOZ	2014	AFR	LIC	68.22	0.75	67.47	35.66	56.98	3.68	53.30	78.99
Mozambique	MOZ	2015	AFR	LIC	68.15	1.93	66.22	34.27	60.83	3.93	56.90	85.92
Mozambique	MOZ	2016	AFR	LIC	82.20	2.35	79.85	53.58	73.90	4.78	69.12	86.57
Mozambique	MOZ	2017	AFR	LIC	94.13	2.40	91.73	75.90	83.25	5.38	77.86	84.88
Mozambique	MOZ	2018	AFR	LIC	97.93	1.47	96.46	85.15	83.61	5.40	78.21	81.08
Mozambique	MOZ	2019	AFR	LIC	99.43	1.96	97.47	87.58	86.31	5.58	80.73	82.82
Mauritania	MRT	2010	AFR	LMIC	57.51	0.50	57.01	73.02	46.69	3.02	43.67	76.61
Mauritania	MRT	2011	AFR	LMIC	44.10	0.92	43.18	49.73	38.42	2.48	35.94	83.22
Mauritania	MRT	2012	AFR	LMIC	64.55	0.61	63.93	84.51	53.15	3.44	49.72	77.77
Mauritania	MRT	2013	AFR	LMIC	56.99	0.61	56.38	71.16	47.48	3.07	44.41	78.77
Mauritania	MRT	2014	AFR	LMIC	61.22	0.58	60.64	78.71	50.45	3.26	47.19	77.82
Mauritania	MRT	2015	AFR	LMIC	55.55	0.51	55.04	69.30	45.56	2.94	42.61	77.42
Mauritania	MRT	2016	AFR	LMIC	62.18	0.56	61.61	80.63	50.91	3.29	47.62	77.29
Mauritania	MRT	2017	AFR	LMIC	64.97	0.57	64.40	86.45	52.29	3.38	48.91	75.95
Mauritania	MRT	2018	AFR	LMIC	66.24	0.87	65.37	86.47	56.01	3.62	52.39	80.14
Mauritania	MRT	2019	AFR	LMIC	68.45	0.60	67.85	92.38	55.36	3.58	51.78	76.32
Malawi	MWI	2010	AFR	LIC	48.79	1.28	47.51	57.10	43.24	2.80	40.45	85.14
Malawi	MWI	2011	AFR	LIC	46.39	1.00	45.38	53.36	40.51	2.62	37.89	83.49
Malawi	MWI	2012	AFR	LIC	51.12	1.47	49.66	60.91	45.68	2.95	42.73	86.04
Malawi	MWI	2013	AFR	LIC	48.41	0.74	47.68	57.09	41.39	2.68	38.71	81.19
Malawi	MWI	2014	AFR	LIC	50.58	1.44	49.14	59.00	45.84	2.96	42.88	87.26
Malawi	MWI	2015	AFR	LIC	55.68	1.66	54.03	66.77	51.11	3.30	47.81	88.48
Malawi	MWI	2016	AFR	LIC	54.21	0.27	53.94	72.66	39.68	2.56	37.12	68.81
Malawi	MWI	2017	AFR	LIC	61.20	0.26	60.94	87.22	44.21	2.86	41.35	67.85
Malawi	MWI	2018	AFR	LIC	60.59	0.51	60.08	83.02	46.74	3.02	43.72	72.77
Malawi	MWI	2019	AFR	LIC	67.36	0.72	66.63	95.94	53.00	3.43	49.58	74.40
Malaysia	MYS	2010	WPR	UMIC	90.89	0.32	90.56	98.30	64.76	4.19	60.58	66.89
Malaysia	MYS	2011	WPR	UMIC	90.23	0.29	89.93	98.29	63.81	4.12	59.69	66.37
Malaysia	MYS	2012	WPR	UMIC	89.83	0.28	89.56	98.28	63.30	4.09	59.21	66.12
Malaysia	MYS	2013	WPR	UMIC	91.35	0.36	91.00	98.31	65.51	4.23	61.27	67.33
Malaysia	MYS	2014	WPR	UMIC	85.77	0.30	85.47	98.22	59.45	3.84	55.61	65.06
Malaysia	MYS	2015	WPR	UMIC	86.37	0.28	86.09	98.23	59.90	3.87	56.03	65.09

Malaysia	MYS	2016	WPR	UMIC	89.21	0.27	88.94	98.27	62.56	4.04	58.51	65.79
Malaysia	MYS	2017	WPR	UMIC	89.20	0.27	88.93	98.27	62.55	4.04	58.51	65.79
Malaysia	MYS	2018	WPR	UMIC	90.60	0.31	90.29	98.30	64.34	4.16	60.18	66.65
Malaysia	MYS	2019	WPR	UMIC	90.76	0.32	90.44	98.30	64.56	4.17	60.39	66.77
Namibia	NAM	2010	AFR	UMIC	60.62	0.18	60.44	87.18	42.22	2.73	39.49	65.33
Namibia	NAM	2011	AFR	UMIC	63.07	0.20	62.87	89.40	44.49	2.88	41.62	66.19
Namibia	NAM	2012	AFR	UMIC	57.95	0.26	57.68	87.15	40.19	2.60	37.59	65.17
Namibia	NAM	2013	AFR	UMIC	61.59	0.19	61.40	87.20	43.22	2.79	40.42	65.84
Namibia	NAM	2014	AFR	UMIC	60.13	0.18	59.95	85.01	42.02	2.72	39.31	65.57
Namibia	NAM	2015	AFR	UMIC	63.67	0.24	63.44	98.32	44.13	2.85	41.28	65.06
Namibia	NAM	2016	AFR	UMIC	60.81	0.22	60.59	91.58	42.14	2.72	39.42	65.06
Namibia	NAM	2017	AFR	UMIC	57.27	0.80	56.47	93.77	40.15	2.60	37.55	66.50
Namibia	NAM	2018	AFR	UMIC	60.01	0.21	59.80	89.36	41.59	2.69	38.90	65.06
Namibia	NAM	2019	AFR	UMIC	63.66	0.21	63.46	96.07	44.17	2.85	41.31	65.10
Niger	NER	2010	AFR	LIC	63.04	1.98	61.06	73.07	58.49	3.78	54.71	89.59
Niger	NER	2011	AFR	LIC	66.68	2.62	64.06	76.79	64.90	4.20	60.71	94.76
Niger	NER	2012	AFR	LIC	71.14	3.44	67.71	82.46	72.80	4.71	68.09	100.57
Niger	NER	2013	AFR	LIC	70.96	2.57	68.39	84.33	67.72	4.38	63.34	92.62
Niger	NER	2014	AFR	LIC	65.62	1.88	63.75	78.63	59.58	3.85	55.73	87.42
Niger	NER	2015	AFR	LIC	64.30	2.52	61.78	73.09	62.58	4.05	58.54	94.75
Niger	NER	2016	AFR	LIC	61.81	2.37	59.44	69.44	59.82	3.87	55.95	94.14
Niger	NER	2017	AFR	LIC	63.11	2.00	61.11	73.08	58.67	3.79	54.88	89.81
Niger	NER	2018	AFR	LIC	60.19	0.58	59.61	74.85	47.94	3.10	44.85	75.23
Niger	NER	2019	AFR	LIC	68.59	2.08	66.51	82.41	63.17	4.08	59.08	88.83
Nigeria	NGA	2010	AFR	LMIC	26.82	0.29	26.53	27.74	22.34	1.44	20.89	78.75
Nigeria	NGA	2011	AFR	LMIC	26.67	0.25	26.43	27.74	21.89	1.41	20.48	77.48
Nigeria	NGA	2012	AFR	LMIC	27.65	0.24	27.41	28.89	22.45	1.45	21.00	76.61
Nigeria	NGA	2013	AFR	LMIC	27.46	0.25	27.21	28.89	22.01	1.42	20.59	75.65
Nigeria	NGA	2014	AFR	LMIC	23.69	0.17	23.52	25.41	17.95	1.16	16.79	71.39
Nigeria	NGA	2015	AFR	LMIC	23.61	0.15	23.46	25.40	17.70	1.14	16.56	70.58
Nigeria	NGA	2016	AFR	LMIC	25.61	0.14	25.47	27.72	18.92	1.22	17.69	69.46
Nigeria	NGA	2017	AFR	LMIC	25.69	0.15	25.54	27.72	19.12	1.24	17.89	70.04
Nigeria	NGA	2018	AFR	LMIC	25.92	0.20	25.72	27.73	19.87	1.28	18.59	72.26
Nigeria	NGA	2019	AFR	LMIC	29.23	0.25	28.98	31.21	22.62	1.46	21.15	73.00
Nicaragua	NIC	2010	AMR	LMIC	86.95	0.97	85.98	99.27	68.58	4.43	64.15	74.61
Nicaragua	NIC	2011	AMR	LMIC	87.72	0.81	86.91	99.29	70.09	4.53	65.56	75.44
Nicaragua	NIC	2012	AMR	LMIC	86.79	0.84	85.95	99.27	67.80	4.38	63.42	73.79
Nicaragua	NIC	2013	AMR	LMIC	82.87	0.26	82.61	99.15	58.55	3.78	54.77	66.30
Nicaragua	NIC	2014	AMR	LMIC	82.42	0.25	82.17	99.15	57.99	3.75	54.24	66.01
Nicaragua	NIC	2015	AMR	LMIC	80.65	0.24	80.41	99.12	56.12	3.63	52.50	65.29
Nicaragua	NIC	2016	AMR	LMIC	79.88	0.25	79.63	99.11	55.45	3.58	51.87	65.14
Nicaragua	NIC	2017	AMR	LMIC	77.91	0.31	77.59	99.08	54.00	3.49	50.51	65.09
Nicaragua	NIC	2018	AMR	LMIC	76.66	0.39	76.27	99.07	53.22	3.44	49.78	65.27
Nicaragua	NIC	2019	AMR	LMIC	77.08	0.36	76.72	99.08	53.47	3.46	50.02	65.19
Nepal	NPL	2010	SEA	LMIC	47.34	0.78	46.56	75.86	40.65	2.63	38.02	81.66
Nepal	NPL	2011	SEA	LMIC	48.21	0.60	47.62	77.86	40.59	2.62	37.97	79.74
Nepal	NPL	2012	SEA	LMIC	50.99	1.23	49.76	81.91	44.91	2.90	42.01	84.42
Nepal	NPL	2013	SEA	LMIC	49.29	0.58	48.70	79.87	41.36	2.67	38.69	79.43

Nepal	NPL	2014	SEA	LMIC	52.84	0.47	52.37	87.96	42.50	2.75	39.75	75.91
Nepal	NPL	2015	SEA	LMIC	50.82	0.44	50.38	83.90	41.16	2.66	38.50	76.43
Nepal	NPL	2016	SEA	LMIC	48.28	0.44	47.84	79.85	38.62	2.50	36.12	75.52
Nepal	NPL	2017	SEA	LMIC	50.03	0.46	49.56	81.88	41.09	2.66	38.43	77.54
Nepal	NPL	2018	SEA	LMIC	50.20	0.51	49.69	83.89	39.83	2.57	37.25	74.97
Nepal	NPL	2019	SEA	LMIC	50.75	0.44	50.30	83.90	40.99	2.65	38.34	76.21
Pakistan	PAK	2010	EMR	LMIC	61.95	1.97	59.98	50.79	57.61	3.72	53.89	89.83
Pakistan	PAK	2011	EMR	LMIC	63.04	3.40	59.64	49.41	66.40	4.29	62.11	104.14
Pakistan	PAK	2012	EMR	LMIC	62.96	3.31	59.65	49.41	65.88	4.26	61.62	103.30
Pakistan	PAK	2013	EMR	LMIC	63.37	1.82	61.56	53.65	57.60	3.72	53.87	87.52
Pakistan	PAK	2014	EMR	LMIC	69.21	2.79	66.42	58.06	67.82	4.38	63.44	95.51
Pakistan	PAK	2015	EMR	LMIC	70.23	2.26	67.97	61.03	65.49	4.23	61.25	90.11
Pakistan	PAK	2016	EMR	LMIC	78.83	4.29	74.54	68.74	83.31	5.38	77.92	104.54
Pakistan	PAK	2017	EMR	LMIC	74.31	2.12	72.19	68.68	66.91	4.32	62.58	86.69
Pakistan	PAK	2018	EMR	LMIC	73.50	2.10	71.40	67.13	66.52	4.30	62.22	87.15
Pakistan	PAK	2019	EMR	LMIC	68.17	2.30	65.87	58.04	64.10	4.14	59.96	91.02
Peru	PER	2010	AMR	UMIC	84.37	0.36	84.01	92.11	60.89	3.94	56.95	67.79
Peru	PER	2011	AMR	UMIC	85.04	0.42	84.62	92.13	62.25	4.02	58.23	68.81
Peru	PER	2012	AMR	UMIC	78.48	0.29	78.19	92.02	54.39	3.52	50.87	65.06
Peru	PER	2013	AMR	UMIC	77.54	0.33	77.21	92.01	53.76	3.48	50.29	65.13
Peru	PER	2014	AMR	UMIC	81.55	0.24	81.31	92.06	57.00	3.68	53.32	65.57
Peru	PER	2015	AMR	UMIC	78.47	0.29	78.18	92.02	54.38	3.52	50.87	65.06
Peru	PER	2016	AMR	UMIC	80.23	0.25	79.99	92.04	55.75	3.60	52.15	65.20
Peru	PER	2017	AMR	UMIC	78.17	0.30	77.87	92.02	54.18	3.50	50.67	65.07
Peru	PER	2018	AMR	UMIC	75.45	0.91	74.55	99.16	52.57	3.40	49.17	65.96
Peru	PER	2019	AMR	UMIC	74.55	1.10	73.45	96.74	52.33	3.38	48.95	66.65
Philippines	PHL	2010	WPR	LMIC	35.77	0.33	35.44	32.89	27.84	1.80	26.04	73.48
Philippines	PHL	2011	WPR	LMIC	41.29	0.45	40.84	40.43	32.54	2.10	30.43	74.52
Philippines	PHL	2012	WPR	LMIC	45.05	0.41	44.64	45.19	36.12	2.33	33.78	75.67
Philippines	PHL	2013	WPR	LMIC	47.33	0.42	46.91	48.45	38.06	2.46	35.60	75.89
Philippines	PHL	2014	WPR	LMIC	48.38	0.43	47.94	50.10	38.83	2.51	36.32	75.75
Philippines	PHL	2015	WPR	LMIC	54.06	0.47	53.58	58.64	43.66	2.82	40.84	76.21
Philippines	PHL	2016	WPR	LMIC	64.62	0.63	63.99	75.05	53.36	3.45	49.91	78.01
Philippines	PHL	2017	WPR	LMIC	60.34	0.64	59.70	67.61	50.26	3.25	47.01	78.75
Philippines	PHL	2018	WPR	LMIC	70.40	0.75	69.65	84.70	58.64	3.79	54.85	78.76
Philippines	PHL	2019	WPR	LMIC	76.13	0.88	75.26	94.71	63.80	4.12	59.67	79.29
Papua New Guinea	PNG	2010	WPR	LMIC	50.00	0.56	49.44	28.00	39.46	2.55	36.91	74.65
Papua New Guinea	PNG	2011	WPR	LMIC	49.96	0.52	49.43	28.00	39.24	2.54	36.70	74.24
Papua New Guinea	PNG	2012	WPR	LMIC	67.05	0.55	66.50	52.98	51.63	3.34	48.29	72.62
Papua New Guinea	PNG	2013	WPR	LMIC	72.17	0.52	71.65	62.52	54.75	3.54	51.22	71.47
Papua New Guinea	PNG	2014	WPR	LMIC	84.30	0.75	83.55	81.90	68.88	4.45	64.43	77.11
Papua New Guinea	PNG	2015	WPR	LMIC	80.77	0.58	80.19	79.56	61.22	3.96	57.26	71.40
Papua New Guinea	PNG	2016	WPR	LMIC	85.37	0.76	84.61	84.22	69.67	4.50	65.17	77.02
Papua New Guinea	PNG	2017	WPR	LMIC	79.27	0.70	78.57	72.96	63.78	4.12	59.66	75.93
Papua New Guinea	PNG	2018	WPR	LMIC	81.41	0.82	80.59	79.58	63.77	4.12	59.65	74.02
Papua New Guinea	PNG	2019	WPR	LMIC	85.52	0.74	84.78	88.92	66.16	4.28	61.88	72.99
Democratic People's Republic of Korea	PRK	2010	SEA	LIC	74.27	0.66	73.61	54.26	60.59	3.92	56.68	76.99

Democratic People's Republic of Korea	PRK	2011	SEA	LIC	79.79	0.71	79.08	64.10	65.05	4.20	60.84	76.93
Democratic People's Republic of Korea	PRK	2012	SEA	LIC	80.61	0.93	79.68	64.12	67.55	4.37	63.18	79.29
Democratic People's Republic of Korea	PRK	2013	SEA	LIC	85.62	1.36	84.26	72.68	73.36	4.74	68.62	81.44
Democratic People's Republic of Korea	PRK	2014	SEA	LIC	88.75	0.79	87.95	81.80	72.53	4.69	67.84	77.13
Democratic People's Republic of Korea	PRK	2015	SEA	LIC	96.71	0.90	95.82	99.31	79.42	5.13	74.29	77.53
Democratic People's Republic of Korea	PRK	2016	SEA	LIC	96.37	0.85	95.51	99.30	78.47	5.07	73.40	76.85
Democratic People's Republic of Korea	PRK	2017	SEA	LIC	85.14	0.75	84.40	74.87	69.09	4.47	64.62	76.56
Democratic People's Republic of Korea	PRK	2018	SEA	LIC	76.31	0.67	75.64	58.08	61.93	4.00	57.93	76.59
Democratic People's Republic of Korea	PRK	2019	SEA	LIC	81.53	0.85	80.68	66.19	67.81	4.38	63.42	78.61
Paraguay	PRY	2010	AMR	UMIC	93.01	0.54	92.48	98.37	68.99	4.46	64.53	69.78
Paraguay	PRY	2011	AMR	UMIC	90.57	0.31	90.27	98.31	64.30	4.16	60.14	66.62
Paraguay	PRY	2012	AMR	UMIC	90.87	0.32	90.55	98.32	64.74	4.18	60.55	66.87
Paraguay	PRY	2013	AMR	UMIC	91.65	0.38	91.27	98.34	66.03	4.27	61.76	67.67
Paraguay	PRY	2014	AMR	UMIC	85.84	0.30	85.54	98.25	59.50	3.85	55.66	65.06
Paraguay	PRY	2015	AMR	UMIC	89.14	0.27	88.88	98.29	62.49	4.04	58.45	65.76
Paraguay	PRY	2016	AMR	UMIC	87.92	0.26	87.66	98.27	61.23	3.96	57.28	65.34
Paraguay	PRY	2017	AMR	UMIC	87.47	0.26	87.20	98.27	60.82	3.93	56.89	65.23
Paraguay	PRY	2018	AMR	UMIC	87.24	0.27	86.98	98.26	60.62	3.92	56.70	65.19
Paraguay	PRY	2019	AMR	UMIC	87.04	0.27	86.77	98.26	60.45	3.91	56.54	65.16
Russian Federation	RUS	2010	EUR	UMIC	90.91	0.75	90.16	98.68	62.11	4.01	58.10	64.44
Russian Federation	RUS	2011	EUR	UMIC	88.18	0.68	87.50	88.65	59.97	3.88	56.09	64.11
Russian Federation	RUS	2012	EUR	UMIC	87.27	0.52	86.76	85.44	57.92	3.74	54.18	62.45
Russian Federation	RUS	2013	EUR	UMIC	90.91	0.48	90.43	98.61	59.44	3.84	55.60	61.49
Russian Federation	RUS	2014	EUR	UMIC	90.91	0.46	90.45	98.59	58.77	3.80	54.97	60.78
Russian Federation	RUS	2015	EUR	UMIC	90.91	0.56	90.35	98.64	60.54	3.91	56.62	62.67
Russian Federation	RUS	2016	EUR	UMIC	90.91	0.62	90.29	98.66	61.17	3.95	57.22	63.37
Russian Federation	RUS	2017	EUR	UMIC	90.00	0.49	89.51	95.20	59.04	3.82	55.22	61.69
Russian Federation	RUS	2018	EUR	UMIC	90.00	0.47	89.53	95.19	58.70	3.79	54.91	61.33
Russian Federation	RUS	2019	EUR	UMIC	90.91	0.46	90.45	98.59	58.74	3.80	54.94	60.74
Rwanda	RWA	2010	AFR	LIC	88.84	0.81	88.03	96.21	72.78	4.70	68.08	77.33
Rwanda	RWA	2011	AFR	LIC	86.94	0.96	85.98	96.17	68.54	4.43	64.11	74.56
Rwanda	RWA	2012	AFR	LIC	89.41	0.96	88.45	96.22	74.51	4.82	69.69	78.79
Rwanda	RWA	2013	AFR	LIC	86.73	0.81	85.92	96.16	67.54	4.37	63.18	73.53
Rwanda	RWA	2014	AFR	LIC	84.53	0.37	84.16	96.09	61.18	3.95	57.23	68.00
Rwanda	RWA	2015	AFR	LIC	84.27	0.35	83.92	96.08	60.70	3.92	56.78	67.66
Rwanda	RWA	2016	AFR	LIC	83.49	0.29	83.20	96.06	59.42	3.84	55.58	66.81
Rwanda	RWA	2017	AFR	LIC	84.12	0.34	83.78	96.08	60.44	3.91	56.53	67.47
Rwanda	RWA	2018	AFR	LIC	85.20	0.44	84.75	96.10	62.60	4.05	58.56	69.09
Rwanda	RWA	2019	AFR	LIC	84.66	0.38	84.28	96.09	61.44	3.97	57.47	68.19
Sudan	SDN	2010	EMR	LIC	56.23	0.80	55.42	49.81	47.84	3.09	44.75	80.75
Sudan	SDN	2011	EMR	LIC	56.45	0.57	55.88	52.83	44.23	2.86	41.37	74.05
Sudan	SDN	2012	EMR	LIC	56.31	0.87	55.44	49.81	48.19	3.11	45.07	81.31
Sudan	SDN	2013	EMR	LIC	57.38	0.50	56.88	52.84	46.37	3.00	43.38	76.26
Sudan	SDN	2014	EMR	LIC	59.17	0.55	58.62	55.94	47.23	3.05	44.18	75.37
Sudan	SDN	2015	EMR	LIC	61.12	0.56	60.56	57.53	50.15	3.24	46.90	77.46
Sudan	SDN	2016	EMR	LIC	69.31	0.62	68.69	70.66	55.69	3.60	52.09	75.83
Sudan	SDN	2017	EMR	LIC	70.53	0.69	69.83	74.07	55.13	3.56	51.57	73.85

Sudan	SDN	2018	EMR	LIC	75.99	1.94	74.05	77.62	67.22	4.34	62.87	84.91
Sudan	SDN	2019	EMR	LIC	76.02	0.67	75.35	81.11	61.25	3.96	57.29	76.03
Senegal	SEN	2010	AFR	LMIC	73.92	0.87	73.05	76.17	62.01	4.01	58.00	79.39
Senegal	SEN	2011	AFR	LMIC	73.28	1.23	72.05	73.94	62.99	4.07	58.92	81.77
Senegal	SEN	2012	AFR	LMIC	79.88	1.17	78.71	87.85	68.07	4.40	63.67	80.89
Senegal	SEN	2013	AFR	LMIC	83.71	0.86	82.85	97.76	69.48	4.49	64.99	78.44
Senegal	SEN	2014	AFR	LMIC	83.65	0.84	82.81	97.76	69.29	4.48	64.81	78.26
Senegal	SEN	2015	AFR	LMIC	82.76	0.91	81.85	95.23	69.10	4.47	64.63	78.96
Senegal	SEN	2016	AFR	LMIC	79.53	0.94	78.59	87.84	66.73	4.31	62.42	79.42
Senegal	SEN	2017	AFR	LMIC	80.88	1.09	79.79	90.28	68.54	4.43	64.11	80.35
Senegal	SEN	2018	AFR	LMIC	78.40	0.69	77.71	87.81	63.42	4.10	59.32	76.34
Senegal	SEN	2019	AFR	LMIC	78.04	0.77	77.27	85.43	64.57	4.17	60.39	78.16
Solomon Islands	SLB	2010	WPR	LMIC	83.84	0.32	83.52	97.54	59.96	3.88	56.09	67.15
Solomon Islands	SLB	2011	WPR	LMIC	81.34	0.24	81.10	97.49	56.79	3.67	53.12	65.50
Solomon Islands	SLB	2012	WPR	LMIC	77.19	0.35	76.84	97.44	53.54	3.46	50.08	65.18
Solomon Islands	SLB	2013	WPR	LMIC	78.80	0.28	78.52	97.46	54.62	3.53	51.09	65.06
Solomon Islands	SLB	2014	WPR	LMIC	81.83	0.24	81.59	97.50	57.31	3.70	53.60	65.70
Solomon Islands	SLB	2015	WPR	LMIC	82.87	0.26	82.60	97.52	58.55	3.78	54.76	66.30
Solomon Islands	SLB	2016	WPR	LMIC	77.50	0.33	77.16	97.45	53.73	3.47	50.26	65.14
Solomon Islands	SLB	2017	WPR	LMIC	79.36	0.26	79.10	97.47	55.04	3.56	51.48	65.08
Solomon Islands	SLB	2018	WPR	LMIC	79.30	0.26	79.04	97.47	54.99	3.55	51.44	65.08
Solomon Islands	SLB	2019	WPR	LMIC	78.40	0.29	78.11	97.46	54.33	3.51	50.82	65.06
Sierra Leone	SLE	2010	AFR	LIC	66.18	0.26	65.92	66.82	47.48	3.07	44.41	67.38
Sierra Leone	SLE	2011	AFR	LIC	64.09	0.25	63.84	62.88	46.00	2.97	43.02	67.39
Sierra Leone	SLE	2012	AFR	LIC	65.35	0.27	65.08	64.84	47.13	3.05	44.08	67.73
Sierra Leone	SLE	2013	AFR	LIC	58.65	0.22	58.43	53.54	41.92	2.71	39.21	67.10
Sierra Leone	SLE	2014	AFR	LIC	58.70	0.18	58.52	55.34	41.28	2.67	38.61	65.98
Sierra Leone	SLE	2015	AFR	LIC	55.83	0.18	55.66	50.00	39.39	2.55	36.84	66.19
Sierra Leone	SLE	2016	AFR	LIC	66.14	0.26	65.88	66.82	47.41	3.06	44.35	67.32
Sierra Leone	SLE	2017	AFR	LIC	74.90	0.32	74.58	83.66	54.08	3.50	50.58	67.82
Sierra Leone	SLE	2018	AFR	LIC	78.27	0.28	77.99	92.72	55.70	3.60	52.10	66.80
Sierra Leone	SLE	2019	AFR	LIC	81.28	0.35	80.93	97.44	58.73	3.80	54.94	67.88
El Salvador	SLV	2010	AMR	LMIC	80.92	0.24	80.68	99.35	56.37	3.64	52.73	65.36
El Salvador	SLV	2011	AMR	LMIC	80.66	0.24	80.42	99.34	56.13	3.63	52.50	65.29
El Salvador	SLV	2012	AMR	LMIC	80.11	0.25	79.86	99.33	55.64	3.60	52.05	65.17
El Salvador	SLV	2013	AMR	LMIC	80.77	0.24	80.53	99.35	56.23	3.63	52.60	65.32
El Salvador	SLV	2014	AMR	LMIC	80.95	0.24	80.71	99.35	56.40	3.65	52.75	65.37
El Salvador	SLV	2015	AMR	LMIC	75.44	0.50	74.94	99.28	52.55	3.40	49.15	65.58
El Salvador	SLV	2016	AMR	LMIC	83.40	0.29	83.11	99.39	59.29	3.83	55.46	66.73
El Salvador	SLV	2017	AMR	LMIC	84.13	0.34	83.79	99.41	60.45	3.91	56.54	67.48
El Salvador	SLV	2018	AMR	LMIC	77.25	0.35	76.91	99.30	53.58	3.46	50.12	65.17
El Salvador	SLV	2019	AMR	LMIC	77.23	0.35	76.88	99.30	53.56	3.46	50.10	65.17
Somalia	SOM	2010	EMR	LIC	31.22	0.22	31.00	59.27	23.64	1.53	22.11	71.32
Somalia	SOM	2011	EMR	LIC	35.39	0.22	35.17	68.00	26.47	1.71	24.76	70.40
Somalia	SOM	2012	EMR	LIC	35.70	0.30	35.40	68.02	27.53	1.78	25.75	72.74
Somalia	SOM	2013	EMR	LIC	37.86	0.32	37.54	72.41	29.19	1.89	27.30	72.71
Somalia	SOM	2014	EMR	LIC	38.49	0.34	38.14	72.42	30.90	2.00	28.90	75.78
Somalia	SOM	2015	EMR	LIC	40.31	0.43	39.89	76.83	31.91	2.06	29.85	74.83

Somalia	SOM	2016	EMR	LIC	40.55	0.38	40.17	76.83	32.37	2.09	30.28	75.38
Somalia	SOM	2017	EMR	LIC	46.49	0.38	46.11	90.13	35.77	2.31	33.46	72.55
Somalia	SOM	2018	EMR	LIC	47.08	0.57	46.50	87.95	39.59	2.56	37.03	79.64
Somalia	SOM	2019	EMR	LIC	46.42	0.41	46.01	87.93	37.63	2.43	35.19	76.49
Serbia	SRB	2010	EUR	UMIC	79.09	1.14	77.95	99.31	55.50	3.59	51.91	66.60
Serbia	SRB	2011	EUR	UMIC	79.09	0.44	78.65	99.25	52.12	3.37	48.75	61.99
Serbia	SRB	2012	EUR	UMIC	79.09	0.43	78.67	99.24	51.81	3.35	48.46	61.60
Serbia	SRB	2013	EUR	UMIC	79.09	0.53	78.56	99.27	53.12	3.43	49.69	63.24
Serbia	SRB	2014	EUR	UMIC	79.09	0.47	78.62	99.26	52.47	3.39	49.07	62.42
Serbia	SRB	2015	EUR	UMIC	79.09	0.75	78.34	99.30	54.50	3.52	50.98	65.07
Serbia	SRB	2016	EUR	UMIC	79.09	0.84	78.25	99.30	54.81	3.54	51.26	65.51
Serbia	SRB	2017	EUR	UMIC	79.09	0.65	78.44	99.29	53.99	3.49	50.50	64.38
Serbia	SRB	2018	EUR	UMIC	79.09	0.64	78.45	99.29	53.96	3.49	50.47	64.33
Serbia	SRB	2019	EUR	UMIC	79.09	0.56	78.53	99.28	53.46	3.46	50.01	63.68
South Sudan	SSD	2011	AFR	LIC	34.59	0.28	34.31	35.08	26.59	1.72	24.87	72.49
South Sudan	SSD	2012	AFR	LIC	40.07	0.35	39.72	43.27	31.06	2.01	29.05	73.15
South Sudan	SSD	2013	AFR	LIC	29.20	0.24	28.96	27.44	22.47	1.45	21.02	72.59
South Sudan	SSD	2014	AFR	LIC	37.83	0.30	37.52	39.93	29.07	1.88	27.19	72.45
South Sudan	SSD	2015	AFR	LIC	43.01	0.29	42.72	48.42	32.43	2.10	30.33	71.01
South Sudan	SSD	2016	AFR	LIC	47.52	0.37	47.15	55.58	36.38	2.35	34.03	72.19
South Sudan	SSD	2017	AFR	LIC	44.15	0.31	43.84	50.18	33.46	2.16	31.30	71.39
South Sudan	SSD	2018	AFR	LIC	67.50	1.93	65.57	85.00	60.82	3.93	56.89	86.76
South Sudan	SSD	2019	AFR	LIC	74.05	2.14	71.92	97.77	66.43	4.29	62.13	86.40
Sao Tome and Principe	STP	2010	AFR	LMIC	56.79	0.18	56.61	99.58	39.41	2.55	36.86	65.12
Sao Tome and Principe	STP	2011	AFR	LMIC	57.28	0.17	57.11	99.58	39.83	2.57	37.25	65.23
Sao Tome and Principe	STP	2012	AFR	LMIC	55.86	0.21	55.65	99.57	38.71	2.50	36.21	65.06
Sao Tome and Principe	STP	2013	AFR	LMIC	54.92	0.26	54.66	99.56	38.10	2.46	35.64	65.19
Sao Tome and Principe	STP	2014	AFR	LMIC	56.63	0.18	56.45	99.58	39.28	2.54	36.74	65.09
Sao Tome and Principe	STP	2015	AFR	LMIC	55.42	0.23	55.20	99.56	38.42	2.48	35.93	65.10
Sao Tome and Principe	STP	2016	AFR	LMIC	56.86	0.18	56.68	99.58	39.46	2.55	36.91	65.13
Sao Tome and Principe	STP	2017	AFR	LMIC	53.77	0.36	53.41	99.55	37.44	2.42	35.02	65.58
Sao Tome and Principe	STP	2018	AFR	LMIC	56.42	0.19	56.23	99.58	39.12	2.53	36.59	65.07
Sao Tome and Principe	STP	2019	AFR	LMIC	56.09	0.20	55.89	99.57	38.87	2.51	36.36	65.06
Suriname	SUR	2010	AMR	UMIC	72.73	0.70	72.03	99.56	50.14	3.24	46.90	65.11
Suriname	SUR	2011	AMR	UMIC	72.73	0.45	72.28	99.52	48.46	3.13	45.33	62.72
Suriname	SUR	2012	AMR	UMIC	72.73	0.56	72.16	99.54	49.48	3.20	46.28	64.13
Suriname	SUR	2013	AMR	UMIC	72.73	0.61	72.12	99.55	49.74	3.21	46.52	64.51
Suriname	SUR	2014	AMR	UMIC	72.73	0.63	72.10	99.55	49.80	3.22	46.58	64.61
Suriname	SUR	2015	AMR	UMIC	72.73	0.76	71.97	99.57	50.34	3.25	47.09	65.43
Suriname	SUR	2016	AMR	UMIC	74.66	0.64	74.02	99.60	52.14	3.37	48.77	65.89
Suriname	SUR	2017	AMR	UMIC	76.08	0.44	75.64	99.61	52.89	3.42	49.47	65.40
Suriname	SUR	2018	AMR	UMIC	72.73	0.96	71.77	99.58	50.87	3.29	47.58	66.30
Suriname	SUR	2019	AMR	UMIC	72.73	0.53	72.20	99.54	49.25	3.18	46.06	63.80
Eswatini	SWZ	2010	AFR	LMIC	64.91	0.54	64.37	78.52	50.07	3.24	46.83	72.75
Eswatini	SWZ	2011	AFR	LMIC	64.50	0.44	64.07	78.51	48.61	3.14	45.46	70.97
Eswatini	SWZ	2012	AFR	LMIC	60.92	0.18	60.73	78.45	42.50	2.75	39.75	65.45
Eswatini	SWZ	2013	AFR	LMIC	65.18	0.69	64.48	78.53	51.25	3.31	47.94	74.35
Eswatini	SWZ	2014	AFR	LMIC	63.83	0.33	63.51	78.49	46.81	3.03	43.78	68.94

Eswatini	SWZ	2015	AFR	LMIC	61.30	0.18	61.12	78.45	42.90	2.77	40.12	65.65
Eswatini	SWZ	2016	AFR	LMIC	59.18	0.20	58.98	78.43	41.02	2.65	38.37	65.06
Eswatini	SWZ	2017	AFR	LMIC	58.42	0.24	58.19	78.42	40.49	2.62	37.88	65.09
Eswatini	SWZ	2018	AFR	LMIC	57.33	0.30	57.03	78.42	39.82	2.57	37.25	65.31
Eswatini	SWZ	2019	AFR	LMIC	67.20	0.27	66.93	96.44	46.57	3.01	43.56	65.09
Syrian Arab Republic	SYR	2010	EMR	LIC	81.91	0.24	81.67	97.61	57.39	3.71	53.68	65.73
Syrian Arab Republic	SYR	2011	EMR	LIC	86.30	0.65	85.65	97.71	65.81	4.25	61.56	71.87
Syrian Arab Republic	SYR	2012	EMR	LIC	86.47	0.70	85.77	97.72	66.45	4.30	62.16	72.47
Syrian Arab Republic	SYR	2013	EMR	LIC	84.58	0.37	84.20	97.66	61.28	3.96	57.32	68.07
Syrian Arab Republic	SYR	2014	EMR	LIC	99.97	12.51	87.46	97.70	64.11	4.14	59.97	68.57
Syrian Arab Republic	SYR	2015	EMR	LIC	100.00	20.62	79.38	97.69	63.63	4.11	59.52	74.97
Syrian Arab Republic	SYR	2016	EMR	LIC	97.49	5.84	91.65	97.68	62.89	4.07	58.83	64.19
Syrian Arab Republic	SYR	2017	EMR	LIC	99.48	9.31	90.17	97.69	63.53	4.11	59.42	65.90
Syrian Arab Republic	SYR	2018	EMR	LIC	86.89	0.92	85.97	97.74	68.28	4.41	63.87	74.29
Syrian Arab Republic	SYR	2019	EMR	LIC	90.27	1.61	88.66	97.82	77.88	5.03	72.84	82.16
Chad	TCD	2010	AFR	LIC	60.59	0.77	59.81	58.99	51.13	3.30	47.82	79.95
Chad	TCD	2011	AFR	LIC	65.75	1.62	64.12	65.50	57.99	3.75	54.24	84.59
Chad	TCD	2012	AFR	LIC	63.55	0.63	62.92	63.83	52.58	3.40	49.18	78.17
Chad	TCD	2013	AFR	LIC	65.02	0.80	64.22	65.48	54.70	3.54	51.17	79.67
Chad	TCD	2014	AFR	LIC	66.93	0.67	66.26	68.81	55.47	3.59	51.88	78.30
Chad	TCD	2015	AFR	LIC	62.11	0.55	61.56	62.20	50.73	3.28	47.45	77.09
Chad	TCD	2016	AFR	LIC	57.87	0.55	57.32	55.81	47.67	3.08	44.59	77.78
Chad	TCD	2017	AFR	LIC	61.98	1.01	60.97	60.60	53.17	3.44	49.73	81.57
Chad	TCD	2018	AFR	LIC	66.87	1.64	65.23	67.16	58.95	3.81	55.14	84.53
Chad	TCD	2019	AFR	LIC	67.09	0.73	66.36	68.81	56.00	3.62	52.38	78.93
Togo	TGO	2010	AFR	LIC	63.54	0.63	62.92	40.49	52.57	3.40	49.17	78.16
Togo	TGO	2011	AFR	LIC	66.67	0.61	66.06	45.17	54.70	3.54	51.17	77.46
Togo	TGO	2012	AFR	LIC	68.55	0.95	67.60	46.81	58.18	3.76	54.42	80.49
Togo	TGO	2013	AFR	LIC	62.82	0.56	62.26	41.99	48.71	3.15	45.56	73.17
Togo	TGO	2014	AFR	LIC	67.62	0.60	67.02	46.79	55.17	3.57	51.60	76.99
Togo	TGO	2015	AFR	LIC	76.26	0.95	75.31	59.04	64.25	4.15	60.10	79.81
Togo	TGO	2016	AFR	LIC	85.03	0.42	84.61	83.42	62.22	4.02	58.20	68.79
Togo	TGO	2017	AFR	LIC	85.74	0.39	85.35	85.66	62.25	4.02	58.23	68.22
Togo	TGO	2018	AFR	LIC	90.11	0.85	89.26	85.78	74.15	4.79	69.36	77.70
Togo	TGO	2019	AFR	LIC	94.25	0.88	93.37	97.45	75.25	4.86	70.38	75.38
Thailand	THA	2010	SEA	UMIC	59.23	0.42	58.80	37.63	44.88	2.90	41.98	71.38
Thailand	THA	2011	SEA	UMIC	59.64	0.56	59.08	37.64	46.51	3.01	43.50	73.63
Thailand	THA	2012	SEA	UMIC	56.37	0.52	55.85	33.09	43.87	2.84	41.03	73.47
Thailand	THA	2013	SEA	UMIC	60.16	0.58	59.57	37.65	47.88	3.09	44.78	75.17
Thailand	THA	2014	SEA	UMIC	66.40	0.99	65.41	44.26	56.66	3.66	53.00	81.02
Thailand	THA	2015	SEA	UMIC	61.31	0.60	60.70	37.66	50.70	3.28	47.43	78.13
Thailand	THA	2016	SEA	UMIC	73.04	2.09	70.95	53.36	65.63	4.24	61.39	86.53
Thailand	THA	2017	SEA	UMIC	83.62	1.65	81.97	74.42	72.59	4.69	67.90	82.84
Thailand	THA	2018	SEA	UMIC	89.56	1.03	88.53	88.96	75.02	4.85	70.17	79.26
Thailand	THA	2019	SEA	UMIC	93.67	0.93	92.74	99.45	77.57	5.01	72.55	78.23
Tajikistan	TJK	2010	EUR	LIC	75.15	0.26	74.88	75.47	53.49	3.46	50.03	66.81
Tajikistan	TJK	2011	EUR	LIC	75.22	0.35	74.88	89.20	52.18	3.37	48.81	65.18
Tajikistan	TJK	2012	EUR	LIC	77.56	0.23	77.33	86.85	53.96	3.49	50.47	65.27

Tajikistan	TJK	2013	EUR	LIC	68.66	0.22	68.44	68.97	47.64	3.08	44.56	65.10
Tajikistan	TJK	2014	EUR	LIC	78.60	0.23	78.37	86.87	54.98	3.55	51.43	65.62
Tajikistan	TJK	2015	EUR	LIC	80.31	0.27	80.05	96.59	55.70	3.60	52.10	65.08
Tajikistan	TJK	2016	EUR	LIC	82.45	0.24	82.21	96.62	57.59	3.72	53.87	65.53
Tajikistan	TJK	2017	EUR	LIC	79.73	0.24	79.49	91.66	55.50	3.59	51.91	65.31
Tajikistan	TJK	2018	EUR	LIC	76.31	0.23	76.08	82.19	53.29	3.44	49.85	65.52
Tajikistan	TJK	2019	EUR	LIC	79.00	0.44	78.56	79.98	58.39	3.77	54.62	69.52
Timor-Leste	TLS	2010	SEA	LMIC	85.39	0.69	84.70	94.15	65.60	4.24	61.36	72.45
Timor-Leste	TLS	2011	SEA	LMIC	84.97	0.58	84.38	94.13	64.12	4.14	59.98	71.08
Timor-Leste	TLS	2012	SEA	LMIC	73.03	0.48	72.55	66.95	54.86	3.55	51.32	70.73
Timor-Leste	TLS	2013	SEA	LMIC	71.45	0.64	70.80	60.47	57.32	3.70	53.61	75.72
Timor-Leste	TLS	2014	SEA	LMIC	68.41	0.71	67.70	56.31	53.71	3.47	50.24	74.20
Timor-Leste	TLS	2015	SEA	LMIC	61.48	0.56	60.92	43.10	49.23	3.18	46.04	75.58
Timor-Leste	TLS	2016	SEA	LMIC	64.12	0.71	63.41	48.51	50.57	3.27	47.30	74.59
Timor-Leste	TLS	2017	SEA	LMIC	61.51	0.56	60.96	43.10	49.29	3.19	46.11	75.64
Timor-Leste	TLS	2018	SEA	LMIC	65.08	0.63	64.46	50.40	50.82	3.28	47.53	73.74
Timor-Leste	TLS	2019	SEA	LMIC	69.45	0.61	68.84	56.33	56.00	3.62	52.38	76.10
Tonga	TON	2010	WPR	UMIC	79.09	0.37	78.72	98.88	49.91	3.23	46.69	59.31
Tonga	TON	2011	WPR	UMIC	79.09	0.32	78.77	98.81	46.93	3.03	43.89	55.72
Tonga	TON	2012	WPR	UMIC	79.09	0.36	78.73	98.87	49.44	3.20	46.25	58.74
Tonga	TON	2013	WPR	UMIC	79.09	0.36	78.73	98.87	49.32	3.19	46.13	58.59
Tonga	TON	2014	WPR	UMIC	79.09	0.56	78.53	98.96	53.44	3.45	49.98	63.65
Tonga	TON	2015	WPR	UMIC	79.09	1.19	77.90	99.00	55.55	3.59	51.96	66.70
Tonga	TON	2016	WPR	UMIC	79.09	0.42	78.67	98.92	51.76	3.35	48.42	61.55
Tonga	TON	2017	WPR	UMIC	79.09	0.49	78.60	98.94	52.73	3.41	49.33	62.75
Tonga	TON	2018	WPR	UMIC	79.09	0.35	78.74	98.85	48.85	3.16	45.69	58.03
Tonga	TON	2019	WPR	UMIC	79.09	0.33	78.76	98.83	47.68	3.08	44.60	56.63
Tunisia	TUN	2010	EMR	LMIC	85.11	0.43	84.68	97.82	62.41	4.03	58.38	68.94
Tunisia	TUN	2011	EMR	LMIC	86.10	0.60	85.50	97.85	65.11	4.21	60.90	71.23
Tunisia	TUN	2012	EMR	LMIC	87.47	0.85	86.62	97.90	69.59	4.50	65.09	75.14
Tunisia	TUN	2013	EMR	LMIC	87.34	0.88	86.46	97.90	69.33	4.48	64.85	75.01
Tunisia	TUN	2014	EMR	LMIC	88.82	0.80	88.01	97.93	72.71	4.70	68.01	77.27
Tunisia	TUN	2015	EMR	LMIC	86.83	0.87	85.96	97.88	67.98	4.39	63.58	73.97
Tunisia	TUN	2016	EMR	LMIC	86.75	0.82	85.93	97.88	67.61	4.37	63.24	73.60
Tunisia	TUN	2017	EMR	LMIC	86.73	0.81	85.92	97.88	67.55	4.37	63.19	73.54
Tunisia	TUN	2018	EMR	LMIC	86.76	0.83	85.94	97.88	67.69	4.38	63.31	73.67
Tunisia	TUN	2019	EMR	LMIC	86.79	0.84	85.95	97.88	67.81	4.38	63.43	73.80
Turkey	TUR	2010	EUR	UMIC	85.96	0.29	85.67	98.97	59.59	3.85	55.74	65.06
Turkey	TUR	2011	EUR	UMIC	85.91	0.29	85.62	98.97	59.55	3.85	55.70	65.06
Turkey	TUR	2012	EUR	UMIC	83.50	0.41	83.08	98.94	57.95	3.75	54.21	65.25
Turkey	TUR	2013	EUR	UMIC	81.41	0.65	80.76	98.92	56.81	3.67	53.14	65.80
Turkey	TUR	2014	EUR	UMIC	79.09	1.12	77.97	98.90	55.47	3.59	51.88	66.55
Turkey	TUR	2015	EUR	UMIC	81.54	0.63	80.91	98.92	56.88	3.68	53.20	65.76
Turkey	TUR	2016	EUR	UMIC	81.48	0.64	80.84	98.93	56.85	3.67	53.17	65.78
Turkey	TUR	2017	EUR	UMIC	83.15	0.44	82.71	98.94	57.75	3.73	54.02	65.31
Turkey	TUR	2018	EUR	UMIC	83.53	0.41	83.12	98.95	57.98	3.75	54.23	65.24
Turkey	TUR	2019	EUR	UMIC	83.36	0.42	82.93	98.94	57.87	3.74	54.13	65.27
Tuvalu	TUV	2010	WPR	UMIC	79.09	0.44	78.65	99.57	52.08	3.37	48.71	61.94

Tuvalu	TUV	2011	WPR	UMIC	82.87	0.46	82.41	99.68	57.59	3.72	53.87	65.37
Tuvalu	TUV	2012	WPR	UMIC	87.39	0.26	87.12	99.70	60.75	3.93	56.82	65.22
Tuvalu	TUV	2013	WPR	UMIC	85.02	0.33	84.69	99.74	58.92	3.81	55.11	65.07
Tuvalu	TUV	2014	WPR	UMIC	87.78	0.26	87.51	99.83	61.10	3.95	57.15	65.30
Tuvalu	TUV	2015	WPR	UMIC	89.65	0.27	89.38	99.76	63.08	4.08	59.00	66.01
Tuvalu	TUV	2016	WPR	UMIC	86.79	0.27	86.51	99.73	60.24	3.89	56.34	65.13
Tuvalu	TUV	2017	WPR	UMIC	86.33	0.28	86.05	99.72	59.88	3.87	56.01	65.08
Tuvalu	TUV	2018	WPR	UMIC	90.42	0.30	90.12	99.75	64.07	4.14	59.93	66.50
Tuvalu	TUV	2019	WPR	UMIC	94.01	0.75	93.26	99.84	72.17	4.66	67.50	72.38
United Republic of Tanzania	TZA	2010	AFR	LMIC	36.02	0.54	35.48	37.80	30.77	1.99	28.78	81.11
United Republic of Tanzania	TZA	2011	AFR	LMIC	37.29	1.15	36.13	37.82	34.47	2.23	32.24	89.23
United Republic of Tanzania	TZA	2012	AFR	LMIC	37.79	0.29	37.50	42.48	28.90	1.87	27.04	72.10
United Republic of Tanzania	TZA	2013	AFR	LMIC	44.00	0.78	43.22	48.98	37.94	2.45	35.49	82.11
United Republic of Tanzania	TZA	2014	AFR	LMIC	43.16	1.22	41.94	47.35	38.51	2.49	36.02	85.88
United Republic of Tanzania	TZA	2015	AFR	LMIC	45.00	1.30	43.70	48.99	40.98	2.65	38.33	87.71
United Republic of Tanzania	TZA	2016	AFR	LMIC	47.36	0.80	46.56	53.96	40.73	2.63	38.10	81.83
United Republic of Tanzania	TZA	2017	AFR	LMIC	53.33	1.58	51.75	60.81	48.89	3.16	45.73	88.38
United Republic of Tanzania	TZA	2018	AFR	LMIC	59.70	0.94	58.76	73.17	51.13	3.30	47.82	81.38
United Republic of Tanzania	TZA	2019	AFR	LMIC	65.46	0.59	64.87	84.17	53.51	3.46	50.06	77.16
Uganda	UGA	2010	AFR	LIC	75.54	3.27	72.27	63.28	75.39	4.87	70.52	97.57
Uganda	UGA	2011	AFR	LIC	76.05	2.05	74.00	70.36	67.58	4.37	63.21	85.42
Uganda	UGA	2012	AFR	LIC	72.44	2.07	70.37	63.23	65.67	4.24	61.42	87.28
Uganda	UGA	2013	AFR	LIC	69.87	0.62	69.25	63.19	57.07	3.69	53.38	77.09
Uganda	UGA	2014	AFR	LIC	67.11	1.95	65.16	56.42	60.10	3.88	56.21	86.27
Uganda	UGA	2015	AFR	LIC	63.62	2.20	61.42	48.40	60.15	3.89	56.26	91.60
Uganda	UGA	2016	AFR	LIC	59.16	0.56	58.61	48.34	47.21	3.05	44.16	75.35
Uganda	UGA	2017	AFR	LIC	62.53	1.82	60.71	49.95	55.98	3.62	52.36	86.24
Uganda	UGA	2018	AFR	LIC	73.07	1.03	72.04	66.73	62.09	4.01	58.08	80.62
Uganda	UGA	2019	AFR	LIC	84.06	1.02	83.04	85.54	70.68	4.57	66.11	79.61
Ukraine	UKR	2010	EUR	LMIC	60.91	0.69	60.22	49.81	42.33	2.74	39.60	65.76
Ukraine	UKR	2011	EUR	LMIC	71.50	0.21	71.29	56.88	49.74	3.22	46.53	65.26
Ukraine	UKR	2012	EUR	LMIC	88.90	0.30	88.59	97.48	61.62	3.98	57.64	65.06
Ukraine	UKR	2013	EUR	LMIC	82.35	0.30	82.05	83.33	57.07	3.69	53.38	65.06
Ukraine	UKR	2014	EUR	LMIC	69.41	0.53	68.88	62.45	48.41	3.13	45.28	65.73
Ukraine	UKR	2015	EUR	LMIC	73.81	0.23	73.58	62.49	51.23	3.31	47.92	65.13
Ukraine	UKR	2016	EUR	LMIC	72.83	0.30	72.52	64.40	50.49	3.26	47.22	65.11
Ukraine	UKR	2017	EUR	LMIC	70.78	0.30	70.48	60.56	49.07	3.17	45.90	65.13
Ukraine	UKR	2018	EUR	LMIC	70.41	0.52	69.89	64.38	49.10	3.17	45.92	65.71
Ukraine	UKR	2019	EUR	LMIC	82.46	0.74	81.72	64.56	66.19	4.28	61.91	75.76
Venezuela (Bolivarian Republic of)	VEN	2010	AMR	UMIC	84.95	0.41	84.54	99.47	62.06	4.01	58.05	68.66
Venezuela (Bolivarian Republic of)	VEN	2011	AMR	UMIC	83.74	0.31	83.43	99.45	59.80	3.87	55.94	67.05
Venezuela (Bolivarian Republic of)	VEN	2012	AMR	UMIC	82.94	0.27	82.67	99.43	58.64	3.79	54.85	66.35
Venezuela (Bolivarian Republic of)	VEN	2013	AMR	UMIC	84.46	0.36	84.09	99.45	61.05	3.95	57.10	67.91
Venezuela (Bolivarian Republic of)	VEN	2014	AMR	UMIC	92.19	2.64	89.54	99.65	83.80	5.42	78.38	87.53
Venezuela (Bolivarian Republic of)	VEN	2015	AMR	UMIC	92.09	2.63	89.46	99.65	83.60	5.40	78.20	87.41
Venezuela (Bolivarian Republic of)	VEN	2016	AMR	UMIC	92.78	2.76	90.03	99.65	85.12	5.50	79.62	88.44

Venezuela (Bolivarian Republic of)	VEN	2017	AMR	UMIC	96.55	4.74	91.81	99.73	99.21	6.41	92.80	101.07
Venezuela (Bolivarian Republic of)	VEN	2018	AMR	UMIC	95.63	3.94	91.69	99.70	94.31	6.10	88.21	96.21
Venezuela (Bolivarian Republic of)	VEN	2019	AMR	UMIC	95.94	4.17	91.77	99.71	95.82	6.19	89.63	97.66
Viet Nam	VNM	2010	WPR	LMIC	54.37	1.27	53.10	72.07	47.76	3.09	44.67	84.13
Viet Nam	VNM	2011	WPR	LMIC	54.99	0.71	54.28	74.04	46.44	3.00	43.44	80.03
Viet Nam	VNM	2012	WPR	LMIC	58.42	0.80	57.63	80.06	49.55	3.20	46.34	80.42
Viet Nam	VNM	2013	WPR	LMIC	58.53	0.88	57.65	80.07	49.98	3.23	46.75	81.10
Viet Nam	VNM	2014	WPR	LMIC	58.05	0.55	57.50	82.06	46.29	2.99	43.30	75.31
Viet Nam	VNM	2015	WPR	LMIC	60.41	0.55	59.87	86.17	48.41	3.13	45.28	75.64
Viet Nam	VNM	2016	WPR	LMIC	62.62	0.68	61.93	88.26	52.26	3.38	48.88	78.92
Viet Nam	VNM	2017	WPR	LMIC	64.30	0.57	63.73	92.41	52.47	3.39	49.08	77.01
Viet Nam	VNM	2018	WPR	LMIC	63.32	0.58	62.74	90.33	51.91	3.36	48.55	77.39
Viet Nam	VNM	2019	WPR	LMIC	66.72	0.62	66.10	96.63	54.84	3.54	51.30	77.60
Vanuatu	VUT	2010	WPR	LMIC	72.69	0.22	72.48	77.80	50.93	3.29	47.64	65.73
Vanuatu	VUT	2011	WPR	LMIC	64.44	0.39	64.05	71.21	44.83	2.90	41.93	65.47
Vanuatu	VUT	2012	WPR	LMIC	75.41	0.33	75.07	94.12	52.29	3.38	48.91	65.16
Vanuatu	VUT	2013	WPR	LMIC	76.29	0.25	76.04	91.71	52.90	3.42	49.48	65.08
Vanuatu	VUT	2014	WPR	LMIC	66.99	0.23	66.75	71.23	46.43	3.00	43.43	65.06
Vanuatu	VUT	2015	WPR	LMIC	80.44	0.24	80.20	99.13	55.93	3.62	52.32	65.24
Vanuatu	VUT	2016	WPR	LMIC	61.70	0.29	61.41	62.96	42.80	2.77	40.03	65.19
Vanuatu	VUT	2017	WPR	LMIC	61.83	0.23	61.61	60.99	42.85	2.77	40.08	65.06
Vanuatu	VUT	2018	WPR	LMIC	62.08	0.64	61.44	69.09	43.45	2.81	40.64	66.15
Vanuatu	VUT	2019	WPR	LMIC	68.18	0.27	67.92	86.58	35.31	2.28	33.03	48.63
Samoa	WSM	2010	WPR	UMIC	92.48	0.46	92.02	97.05	67.70	4.38	63.32	68.81
Samoa	WSM	2011	WPR	UMIC	94.56	1.05	93.51	97.13	74.58	4.82	69.76	74.61
Samoa	WSM	2012	WPR	UMIC	95.82	0.85	94.98	97.15	77.16	4.99	72.17	75.98
Samoa	WSM	2013	WPR	UMIC	96.09	0.84	95.25	97.16	77.77	5.03	72.75	76.38
Samoa	WSM	2014	WPR	UMIC	94.29	0.87	93.43	97.11	73.33	4.74	68.59	73.42
Samoa	WSM	2015	WPR	UMIC	95.23	0.91	94.33	97.14	75.89	4.91	70.98	75.25
Samoa	WSM	2016	WPR	UMIC	93.86	0.71	93.15	97.09	71.60	4.63	66.97	71.89
Samoa	WSM	2017	WPR	UMIC	96.33	0.85	95.48	97.16	78.37	5.07	73.31	76.78
Samoa	WSM	2018	WPR	UMIC	93.62	0.65	92.97	97.08	70.77	4.57	66.20	71.20
Samoa	WSM	2019	WPR	UMIC	98.10	1.66	96.44	97.22	84.38	5.45	78.92	81.84
Yemen	YEM	2010	EMR	LIC	81.15	0.75	80.40	63.87	64.85	4.19	60.65	75.44
Yemen	YEM	2011	EMR	LIC	81.48	0.83	80.64	62.12	67.64	4.37	63.26	78.45
Yemen	YEM	2012	EMR	LIC	92.17	0.84	91.33	80.99	75.49	4.88	70.61	77.31
Yemen	YEM	2013	EMR	LIC	93.17	0.84	92.33	87.10	72.31	4.67	67.63	73.25
Yemen	YEM	2014	EMR	LIC	87.64	1.20	86.44	71.25	74.33	4.80	69.52	80.43
Yemen	YEM	2015	EMR	LIC	68.36	1.97	66.39	41.45	61.33	3.96	57.37	86.41
Yemen	YEM	2016	EMR	LIC	85.61	3.30	82.31	60.46	82.96	5.36	77.59	94.27
Yemen	YEM	2017	EMR	LIC	89.23	5.75	83.48	62.26	100.00	6.46	93.54	112.05
Yemen	YEM	2018	EMR	LIC	81.05	2.32	78.73	58.70	72.86	4.71	68.15	86.56
Yemen	YEM	2019	EMR	LIC	86.76	3.32	83.43	62.21	83.95	5.43	78.52	94.11
South Africa	ZAF	2010	AFR	UMIC	59.71	0.32	59.39	94.41	44.01	2.84	41.16	69.31
South Africa	ZAF	2011	AFR	UMIC	61.54	0.29	61.24	98.68	44.88	2.90	41.97	68.54
South Africa	ZAF	2012	AFR	UMIC	57.00	0.39	56.61	88.08	43.00	2.78	40.22	71.05
South Africa	ZAF	2013	AFR	UMIC	55.76	0.23	55.53	88.05	40.09	2.59	37.50	67.52

South Africa	ZAF	2014	AFR	UMIC	57.58	0.62	56.96	88.10	45.32	2.93	42.39	74.42
South Africa	ZAF	2015	AFR	UMIC	56.01	0.40	55.61	85.99	42.49	2.75	39.75	71.47
South Africa	ZAF	2016	AFR	UMIC	55.30	0.28	55.03	85.97	40.53	2.62	37.91	68.89
South Africa	ZAF	2017	AFR	UMIC	62.02	0.36	61.66	98.69	46.03	2.98	43.06	69.83
South Africa	ZAF	2018	AFR	UMIC	61.80	0.33	61.47	98.68	45.47	2.94	42.53	69.19
South Africa	ZAF	2019	AFR	UMIC	62.05	0.37	61.69	98.69	46.11	2.98	43.13	69.92
Zambia	ZMB	2010	AFR	LMIC	78.10	2.85	75.25	87.11	74.64	4.82	69.82	92.78
Zambia	ZMB	2011	AFR	LMIC	74.57	2.13	72.44	85.21	67.40	4.36	63.04	87.03
Zambia	ZMB	2012	AFR	LMIC	66.95	0.52	66.43	79.56	51.24	3.31	47.93	72.15
Zambia	ZMB	2013	AFR	LMIC	68.97	0.65	68.32	79.60	56.75	3.67	53.08	77.69
Zambia	ZMB	2014	AFR	LMIC	68.08	0.69	67.38	77.78	56.49	3.65	52.84	78.41
Zambia	ZMB	2015	AFR	LMIC	66.08	0.77	65.31	74.16	55.44	3.58	51.85	79.40
Zambia	ZMB	2016	AFR	LMIC	69.02	0.66	68.36	79.60	56.88	3.68	53.20	77.83
Zambia	ZMB	2017	AFR	LMIC	66.03	0.75	65.28	74.16	55.27	3.57	51.69	79.18
Zambia	ZMB	2018	AFR	LMIC	63.26	0.65	62.61	72.35	50.14	3.24	46.90	74.92
Zambia	ZMB	2019	AFR	LMIC	66.30	0.73	65.56	77.75	52.28	3.38	48.90	74.58
Zimbabwe	ZWE	2010	AFR	LMIC	95.89	2.75	93.15	89.13	86.20	5.57	80.62	86.56
Zimbabwe	ZWE	2011	AFR	LMIC	87.58	1.16	86.42	76.25	74.11	4.79	69.32	80.21
Zimbabwe	ZWE	2012	AFR	LMIC	85.92	0.87	85.05	74.20	71.25	4.61	66.65	78.37
Zimbabwe	ZWE	2013	AFR	LMIC	91.16	1.36	89.80	82.55	77.79	5.03	72.77	81.03
Zimbabwe	ZWE	2014	AFR	LMIC	86.56	0.78	85.78	76.23	70.79	4.58	66.21	77.19
Zimbabwe	ZWE	2015	AFR	LMIC	91.02	1.25	89.77	82.55	77.24	4.99	72.24	80.48
Zimbabwe	ZWE	2016	AFR	LMIC	87.89	0.86	87.03	82.48	68.70	4.44	64.26	73.85
Zimbabwe	ZWE	2017	AFR	LMIC	91.83	1.07	90.76	84.69	77.02	4.98	72.04	79.37
Zimbabwe	ZWE	2018	AFR	LMIC	91.78	0.80	90.98	86.85	74.48	4.81	69.67	76.57
Zimbabwe	ZWE	2019	AFR	LMIC	79.15	0.71	78.44	64.42	63.50	4.10	59.40	75.73

Figure S4. Sensitivity analysis: average efficiency scores of 14 alternative models compared with the main model

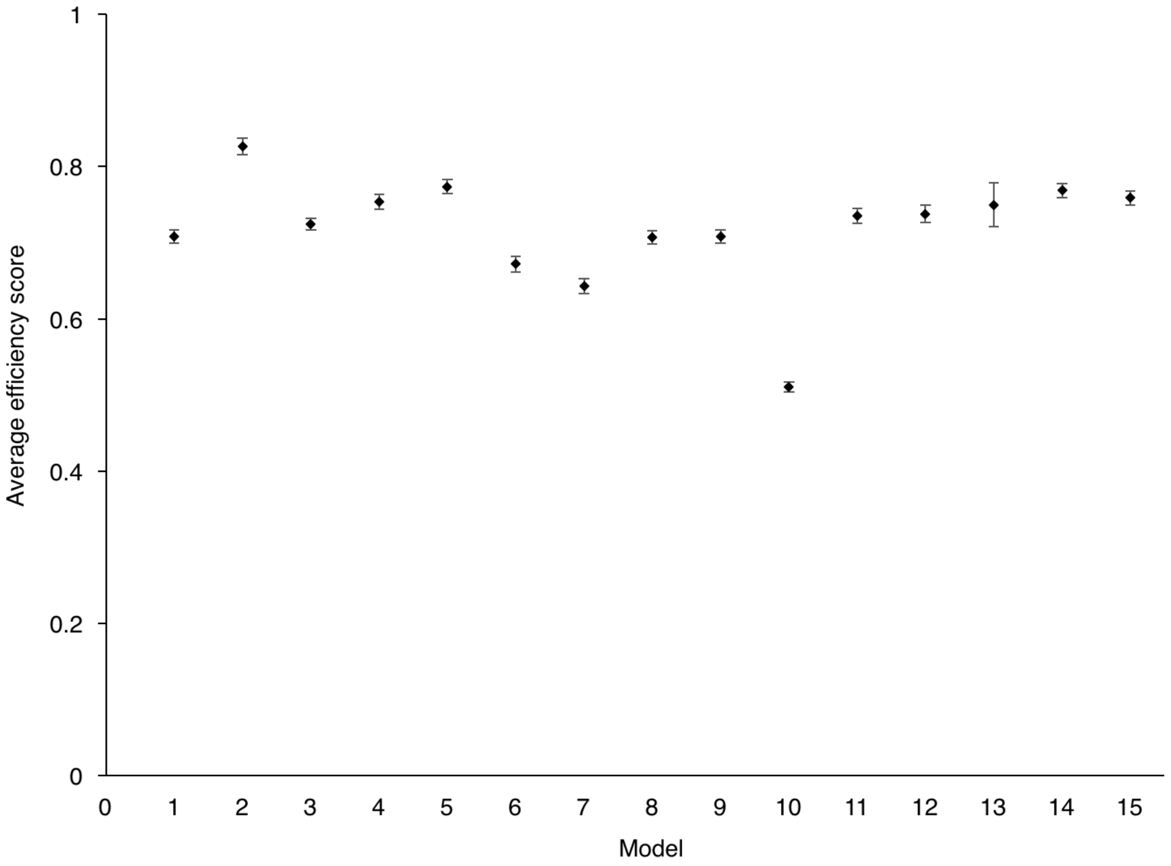

Note: Error bars represent 95% Confidence intervals of the mean efficiency score. Model 1 = Main model. Model 2 = Main SFA based on Greene (2005) true random effects time-varying model using all the variables in the main model. Model 3 = Main model, excluding bottom 5% outlier countries in terms of efficiency scores. Model 4 = Main model, excluding top 5% outlier countries in terms of efficiency scores. Model 5 = Main model, excluding top and bottom 5% outlier countries in terms of efficiency scores. Model 6 = Main model input and independent variables, three outputs using outcome-adjusted treatment coverage for new DS-TB, previously treated DS-TB and MDR-TB. Model 7 = Main model input, two outputs using outcome-adjusted treatment coverage for new DS-TB and previously treated DS-TB. The model excludes MDR-TB related independent variables. Model 8 = Main model, excluding SDG variables listed as included in Table S3 in Supplement 1. Model 9 = Main model, using IHME estimates for sources of national TB spending (out-of-pocket, pre-paid private and development assistance for health) instead of WHO estimates for the proportion of national TB spending accounted for by external sources. Model 10 = Same as main model but assumes constant returns to scale. Model 11 = Main model, assuming 3-year investment lag. Model 12 = Main model, assuming 5-year investment lag. Model 13: Cross-sectional DEA, same as the main model using 10-year average for all variables. Model 14 = SFA based on Battese and Coelli (1998) using all the variables in the main model. Model 15 = SFA based on Pitt and Lee (1991) using all the variables in the main model.

Figure S5a. Graph of average log of input and output by country: 2010-2019

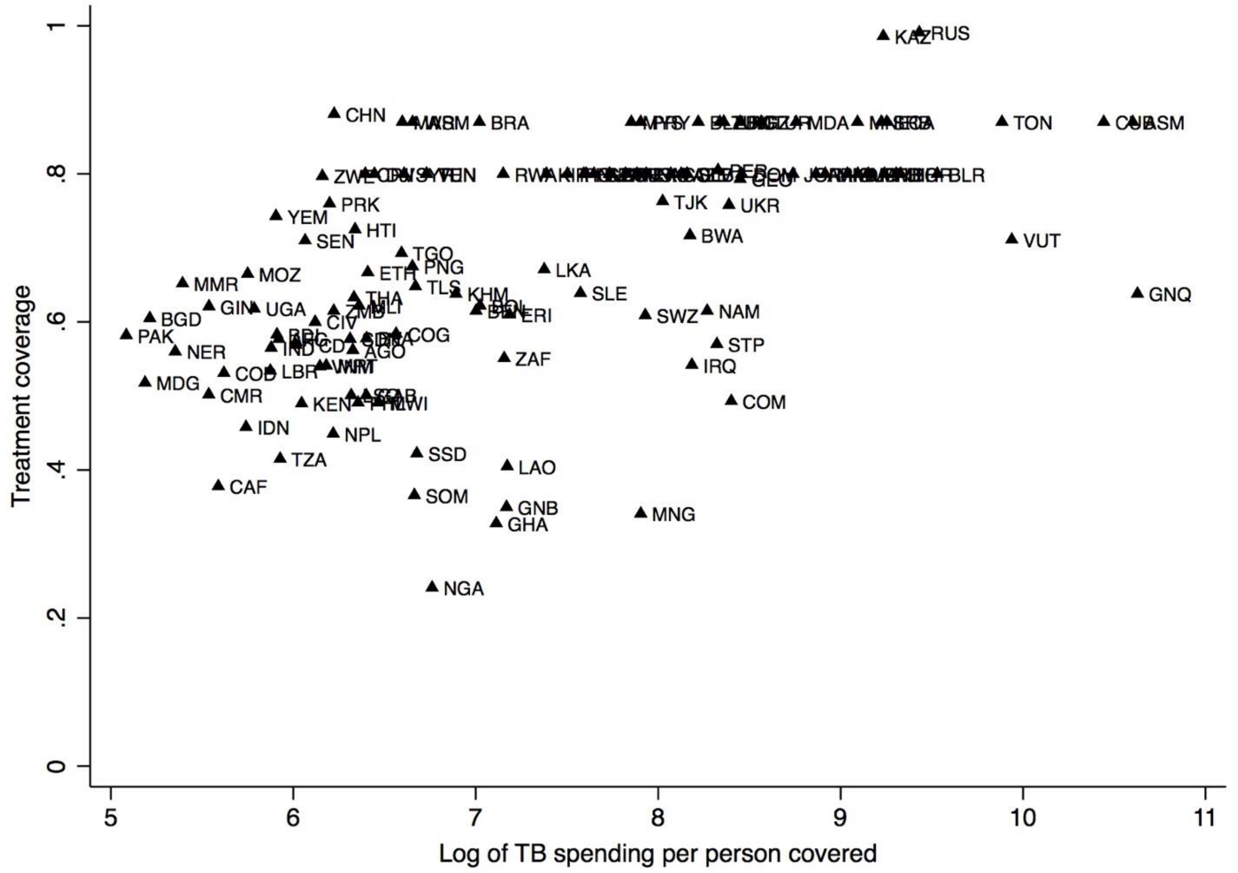

Figure S5b. Graph of average log of input and three outcome-adjusted coverage outputs by country: 2010-2019

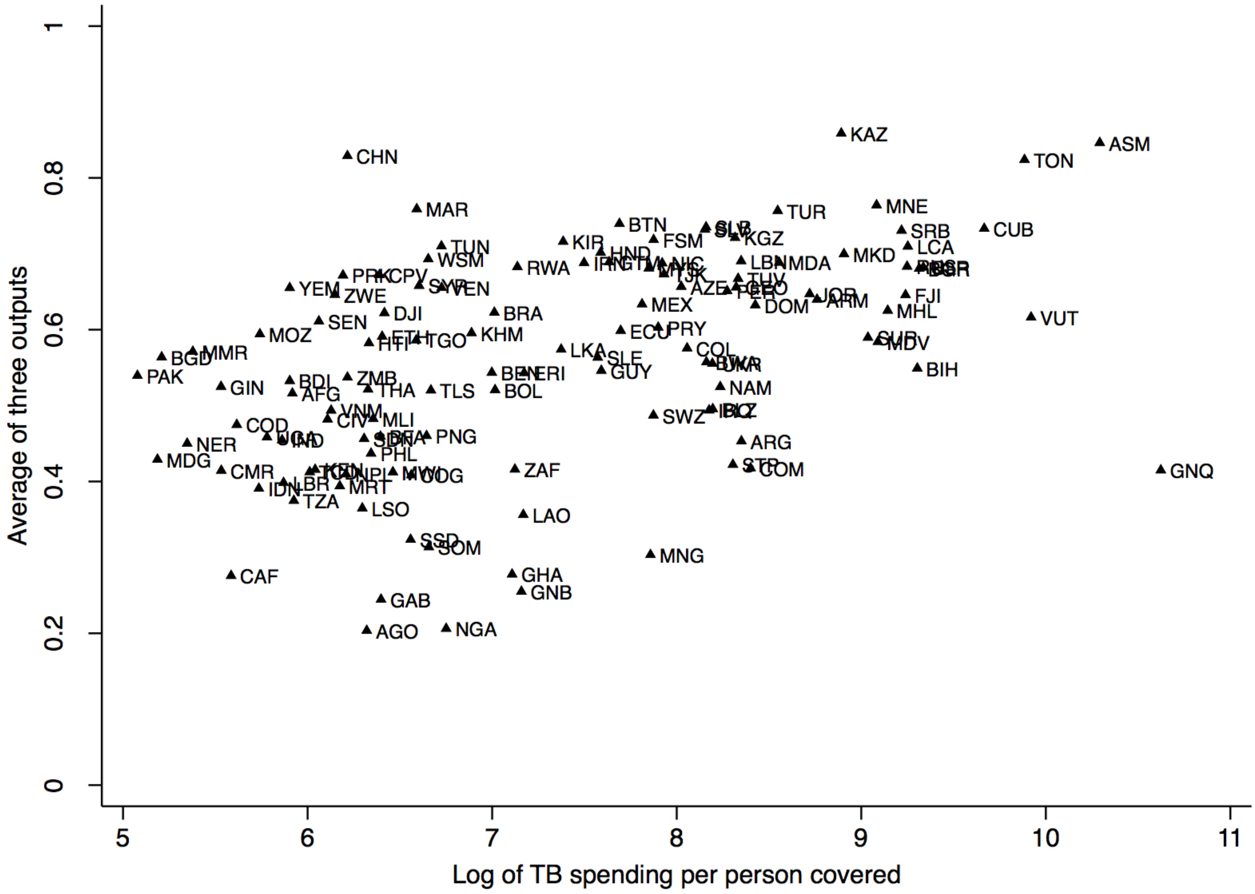

Figure S6. Average DEA and SFA efficiency scores by country between 2010-2019

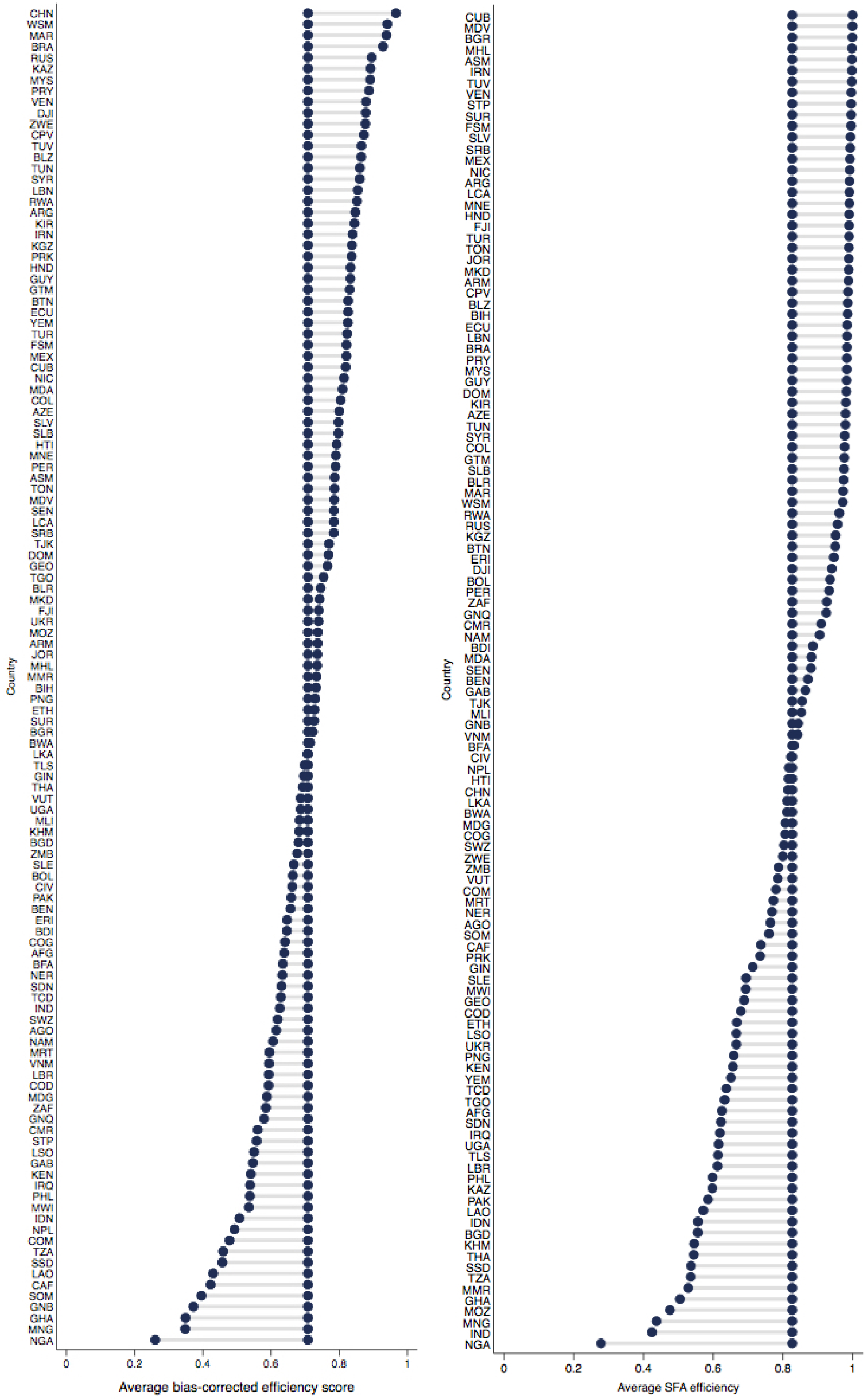

Figures S7a-7f. Descriptive plots of independent variables against bias-corrected efficiency scores (N = 1209 observations).

Figure S7a. Current health expenditure (% GDP)

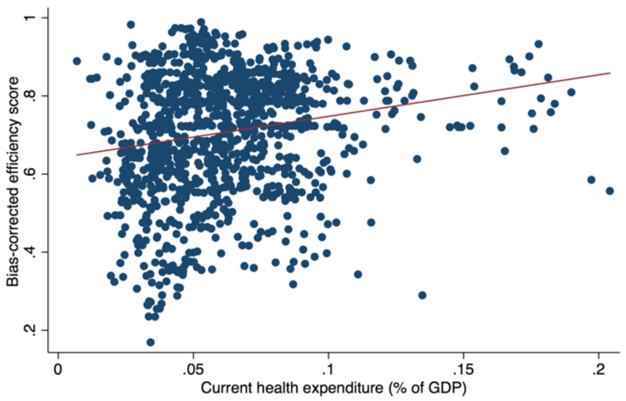

Figure S7b. UHC service coverage index

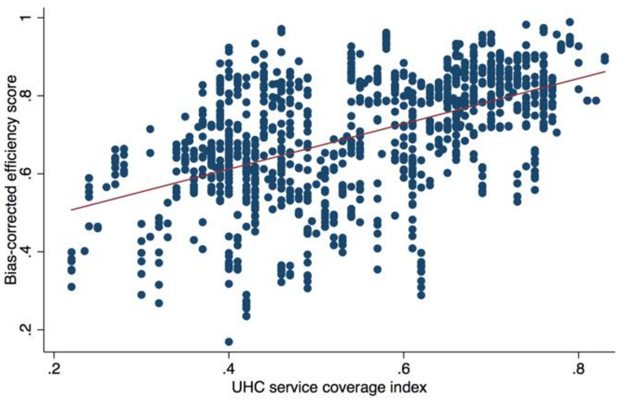

Figure S7c. Governance indicators

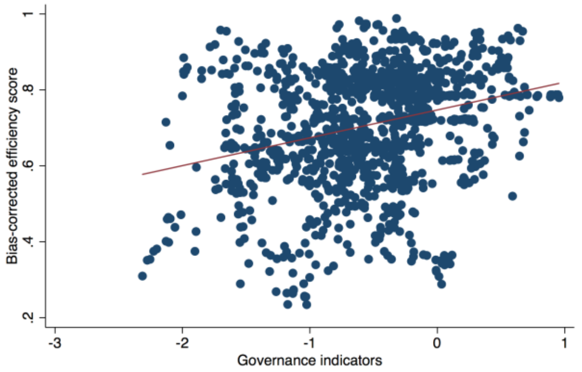

Figure S7d. Out-of-pocket expenditure (%CHE)

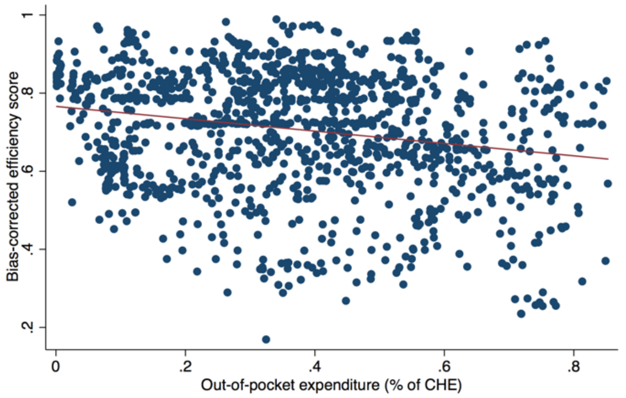

Figure S7e. Population living in slums

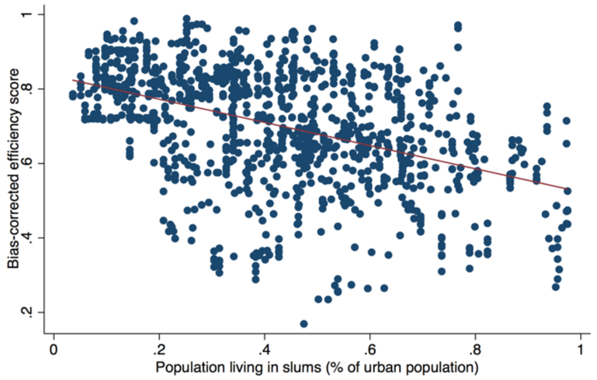

Figure S7f. TB incidence per 100k

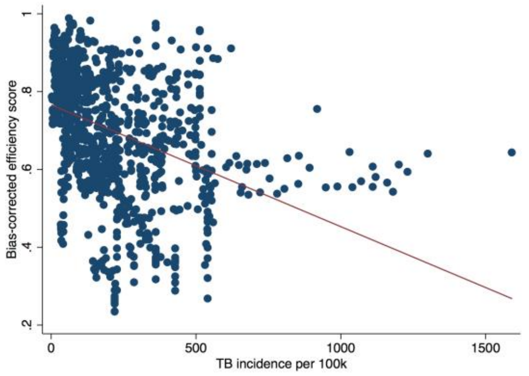

Note: Solid lines show the linear fit between variables on the y- and x-axes.

Figures S7g-7l. Descriptive plots of independent variables against SFA efficiency scores (N = 1209 observations).

Figure S7g. Current health expenditure (% GDP)

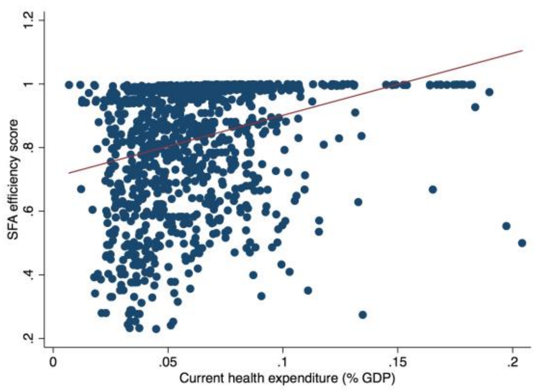

Figure S7h. UHC service coverage index

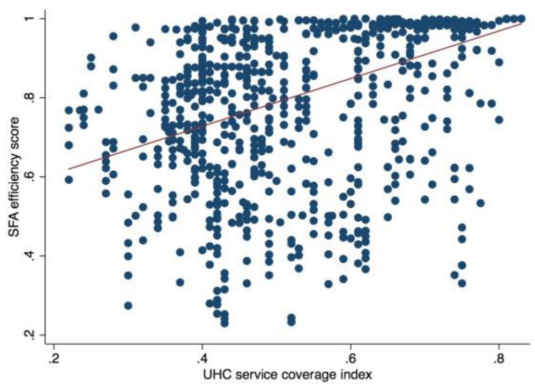

Figure S7i. Governance indicator

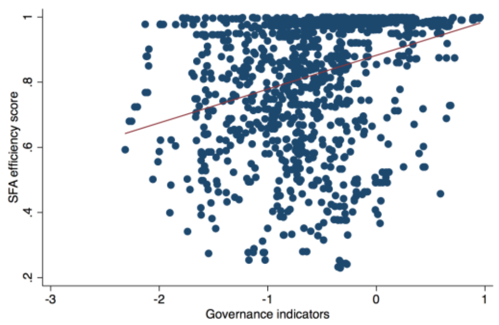

Figure S7j. Out-of-pocket expenditure (%CHE)

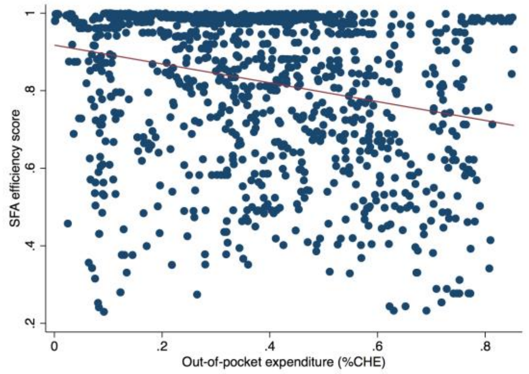

Figure S7k. Population living in slums

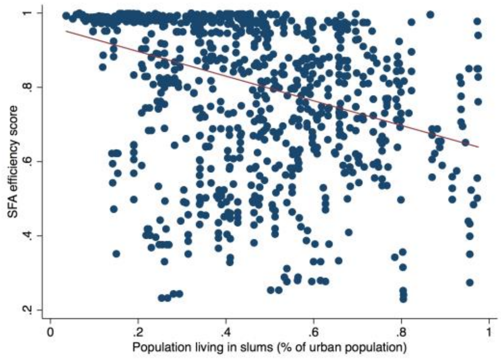

Figure S7l. TB incidence per 100k

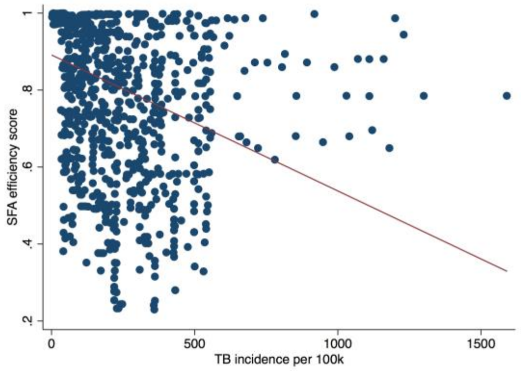

Note: Solid lines show the linear fit between variables on the y- and x-axes.

Figures S8. Main versus sub-regional DEA analyses average scores and rankings

Figure S8a. Main versus sub-regional analysis average DEA efficiency scores

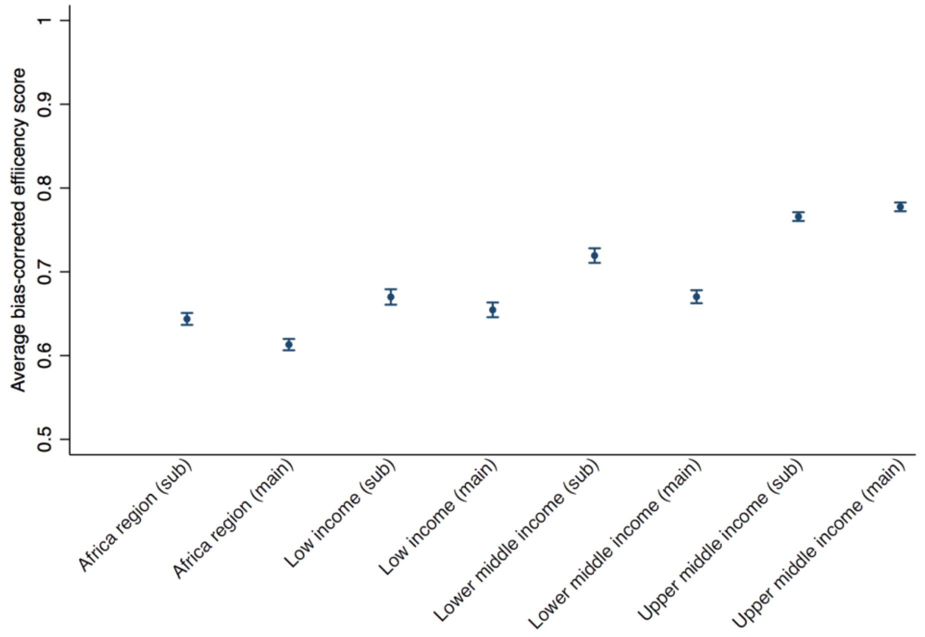

WHO Africa (n=429): ρ=0·9739, p<0·0001; WB Low income (n=279): ρ=0·9888, p<0·0001; WB lower-middle income (n=460): ρ=0·9864, p<0·0001; WB Upper middle-income (n=470): ρ=0·9937, p<0·0001.

Figure S8b. Main versus sub-regional DEA ranking (AFR)

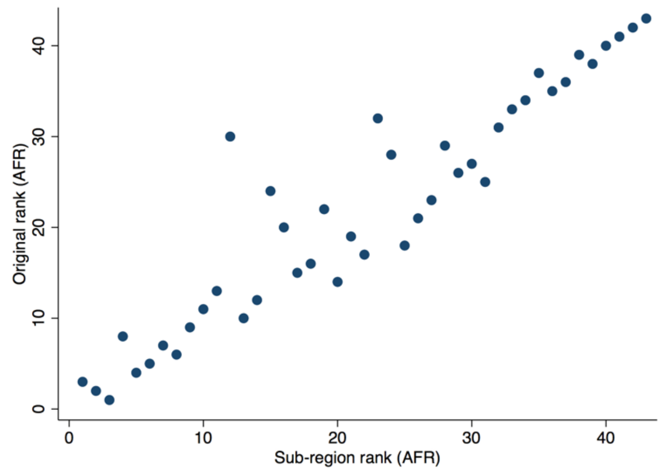

WHO Africa ρ=0·9394, p<0·0001

Figure S8c. Main versus sub-regional DEA ranking (LIC)

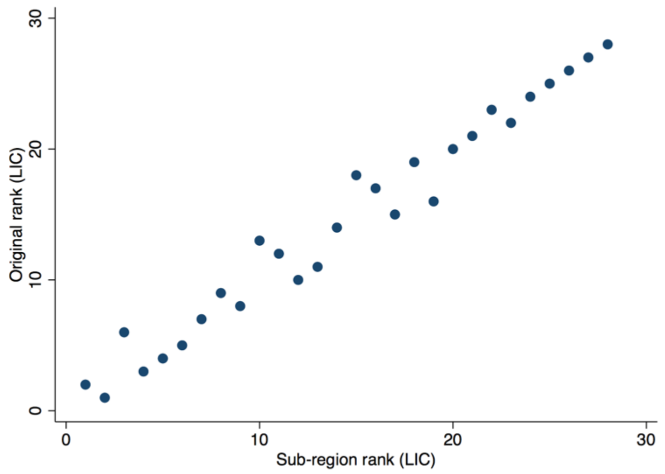

WB Low income ρ=0·9836, p<0·0001

Figure S8d. Main versus sub-regional DEA ranking (LMIC)

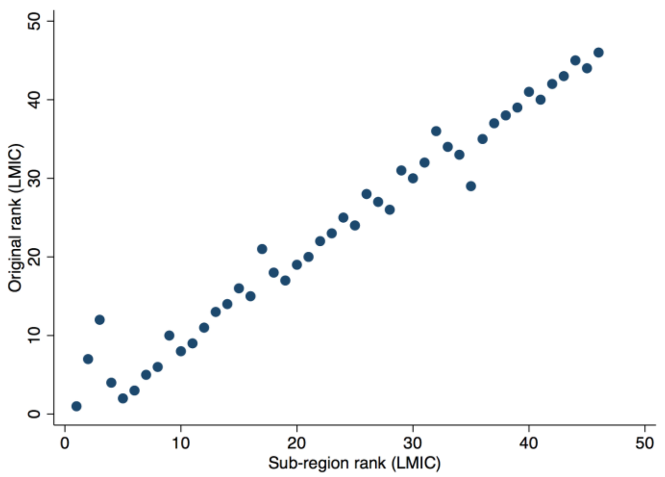

WB Lower-middle income ρ=0·9852, p<0·0001

Figure S8e. Main versus sub-regional DEA ranking (UMIC)

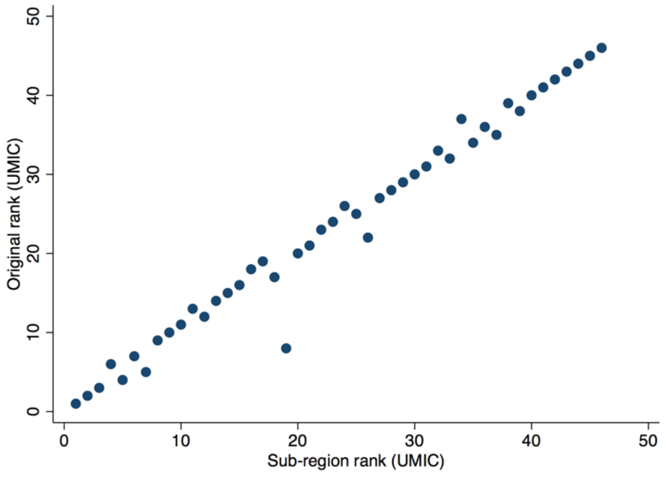

WB Upper-middle income ρ=0·9883, p<0·0001

Table S5. Sub-regional DEA analysis regression results

Independent variables (n=25)	Observed coefficient	Bootstrap standard error	P> z	95% bootstrap confidence intervals	
				Low	High
**Africa region**					
Typical number of visits to a health facility after diagnosis for DS-TB treatment	0.0005	0.0002	0.0070	0.0001	0.0008
Typical number of visits to a health facility after diagnosis for MDR-TB treatment	0.0001	0.0000	0.0100	0.0000	0.0001
Estimated percentage of DS-TB cases that are hospitalised	-0.0389	0.0343	0.2570	-0.1073	0.0281
Estimated percentage of MDR-TB cases that are hospitalised	0.0606	0.0160	0.0000	0.0291	0.0923
Estimated average duration of stay for DS-TB cases if they are hospitalised	-0.0001	0.0004	0.7450	-0.0008	0.0006
Estimated average duration of stay for MDR cases if they are hospitalised	-0.0001	0.0001	0.1050	-0.0002	0.0000
Average of six World Governance Indicators	0.0053	0.0188	0.7760	-0.0316	0.0416
Current health expenditure per capita, PPP (current international $)	0.0000	0.0000	0.8510	-0.0001	0.0001
Current health expenditure (% of GDP)	0.1955	0.2828	0.4890	-0.3715	0.7661
External health expenditure (% of CHE)	-0.0078	0.0569	0.8910	-0.1225	0.1033
Out-of-pocket expenditure (% of CHE)	-0.0254	0.0491	0.6050	-0.1255	0.0702
Population total (log)	0.0000	0.0000	0.0000	0.0000	0.0000
Population per km2	-0.0004	0.0001	0.0000	-0.0005	-0.0002
Rural population (% of total population)	0.1059	0.0659	0.1080	-0.0228	0.2345
TB spending accounted for by external sources (% of total TB spending, excluding private spending)	-0.0964	0.0242	0.0000	-0.1429	-0.0489
TB incidence per 100k	-0.0004	0.0000	0.0000	-0.0005	-0.0003
MDR-TB incidence per 100k	-0.0037	0.0016	0.0170	-0.0069	-0.0006
UHC service coverage index	0.6030	0.1339	0.0000	0.3391	0.8779
Population living in slums (% of urban population)	-0.0974	0.0475	0.0400	-0.1907	-0.0052
Diabetes prevalence (% of population aged >= 18 years)	1.9912	0.7244	0.0060	0.5785	3.3895
Alcohol use disorders, 12-month prevalence (% of population aged >= 15 years)	0.4436	0.2995	0.1390	-0.1432	1.0214
GINI index (0=perfect equality, 100=perfect inequality)	-0.5789	0.1265	0.0000	-0.8239	-0.3225
Population living below the international poverty line (% of population)	-0.1465	0.0491	0.0030	-0.2419	-0.0499
Prevalence of undernourishment (% of population)	-0.2488	0.0585	0.0000	-0.3612	-0.1308
HIV prevalence (adults 15-49)	0.2472	0.2152	0.2510	-0.1662	0.6658
**Low income**					
Typical number of visits to a health facility after diagnosis for DS-TB treatment	0.0000	0.0002	0.8080	-0.0003	0.0003
Typical number of visits to a health facility after diagnosis for MDR-TB treatment	0.0000	0.0000	0.9410	-0.0001	0.0001
Estimated percentage of DS-TB cases that are hospitalised	-0.1598	0.0362	0.0000	-0.2284	-0.0879
Estimated percentage of MDR-TB cases that are hospitalised	0.0109	0.0155	0.4820	-0.0182	0.0415
Estimated average duration of stay for DS-TB cases if they are hospitalised	-0.0001	0.0004	0.6920	-0.0008	0.0006
Estimated average duration of stay for MDR cases if they are hospitalised	0.0001	0.0001	0.0230	0.0000	0.0002

Average of six World Governance Indicators	-0.0617	0.0257	0.0160	-0.1122	-0.0121
Current health expenditure per capita, PPP (current international $)	-0.0003	0.0002	0.1000	-0.0006	0.0001
Current health expenditure (% of GDP)	1.1372	0.4167	0.0060	0.3205	1.9765
External health expenditure (% of CHE)	-0.0294	0.0607	0.6280	-0.1493	0.0910
Out-of-pocket expenditure (% of CHE)	-0.1277	0.0573	0.0260	-0.2374	-0.0147
Population total (log)	0.0000	0.0000	0.0000	0.0000	0.0000
Population per km2	0.0000	0.0001	0.7240	-0.0002	0.0002
Rural population (% of total population)	-0.2256	0.0880	0.0100	-0.4011	-0.0517
TB spending accounted for by external sources (% of total TB spending, excluding private spending)	-0.0731	0.0214	0.0010	-0.1145	-0.0316
TB incidence per 100k	-0.0006	0.0001	0.0000	-0.0008	-0.0004
MDR-TB incidence per 100k	-0.0056	0.0021	0.0070	-0.0099	-0.0017
UHC service coverage index	0.2497	0.1308	0.0560	-0.0068	0.4983
Population living in slums (% of urban population)	-0.1539	0.0552	0.0050	-0.2628	-0.0438
Diabetes prevalence (% of population aged >= 18 years)	1.8080	0.5972	0.0020	0.6010	2.9661
Alcohol use disorders, 12-month prevalence (% of population aged >= 15 years)	-0.3108	0.3755	0.4080	-1.0566	0.4091
GINI index (0=perfect equality, 100=perfect inequality)	0.6855	0.1903	0.0000	0.3104	1.0556
Population living below the international poverty line (% of population)	-0.3013	0.0503	0.0000	-0.3980	-0.2020
Prevalence of undernourishment (% of population)	0.1515	0.0927	0.1020	-0.0284	0.3394
HIV prevalence (adults 15-49)	-0.3009	0.4620	0.5150	-1.2026	0.5974
**Lower-middle income**					
Typical number of visits to a health facility after diagnosis for DS-TB treatment	-0.0004	0.0002	0.0170	-0.0008	0.0000
Typical number of visits to a health facility after diagnosis for MDR-TB treatment	-0.0001	0.0000	0.0020	-0.0002	-0.0001
Estimated percentage of DS-TB cases that are hospitalised	-0.0531	0.0447	0.2350	-0.1381	0.0364
Estimated percentage of MDR-TB cases that are hospitalised	0.0211	0.0297	0.4790	-0.0338	0.0805
Estimated average duration of stay for DS-TB cases if they are hospitalised	0.0007	0.0005	0.1710	-0.0003	0.0017
Estimated average duration of stay for MDR cases if they are hospitalised	0.0002	0.0001	0.1800	-0.0001	0.0004
Average of six World Governance Indicators	0.0728	0.0295	0.0140	0.0140	0.1322
Current health expenditure per capita, PPP (current international $)	-0.0007	0.0001	0.0000	-0.0009	-0.0004
Current health expenditure (% of GDP)	3.8868	0.5948	0.0000	2.7192	5.0208
External health expenditure (% of CHE)	-0.2047	0.0976	0.0360	-0.3986	-0.0085
Out-of-pocket expenditure (% of CHE)	-0.2553	0.0766	0.0010	-0.4093	-0.1107
Population total (log)	0.0000	0.0000	0.5130	0.0000	0.0000
Population per km2	0.0000	0.0000	0.9510	-0.0001	0.0001
Rural population (% of total population)	-0.3100	0.0672	0.0000	-0.4377	-0.1765
TB spending accounted for by external sources (% of total TB spending, excluding private spending)	-0.1784	0.0396	0.0000	-0.2551	-0.0996
TB incidence per 100k	-0.0003	0.0001	0.0000	-0.0004	-0.0002
MDR-TB incidence per 100k	-0.0037	0.0016	0.0180	-0.0069	-0.0007
UHC service coverage index	1.5538	0.2126	0.0000	1.1286	1.9773
Population living in slums (% of urban population)	-0.2258	0.0628	0.0000	-0.3481	-0.1033
Diabetes prevalence (% of population aged >= 18 years)	3.6139	0.4068	0.0000	2.7668	4.3555

Alcohol use disorders, 12-month prevalence (% of population aged >= 15 years)	-2.1094	0.4226	0.0000	-2.9366	-1.2665
GINI index (0=perfect equality, 100=perfect inequality)	-0.5838	0.1658	0.0000	-0.8958	-0.2449
Population living below the international poverty line (% of population)	0.3862	0.1149	0.0010	0.1576	0.6032
Prevalence of undernourishment (% of population)	0.1206	0.1297	0.3520	-0.1306	0.3738
HIV prevalence (adults 15-49)	0.2457	0.3146	0.4350	-0.3734	0.8293
**Upper-middle income**					
Typical number of visits to a health facility after diagnosis for DS-TB treatment	0.0002	0.0001	0.0160	0.0000	0.0003
Typical number of visits to a health facility after diagnosis for MDR-TB treatment	-0.0001	0.0000	0.0040	-0.0001	0.0000
Estimated percentage of DS-TB cases that are hospitalised	-0.0539	0.0166	0.0010	-0.0863	-0.0205
Estimated percentage of MDR-TB cases that are hospitalised	0.0331	0.0107	0.0020	0.0126	0.0536
Estimated average duration of stay for DS-TB cases if they are hospitalised	0.0002	0.0003	0.4070	-0.0003	0.0007
Estimated average duration of stay for MDR cases if they are hospitalised	0.0000	0.0001	0.8050	-0.0001	0.0001
Average of six World Governance Indicators	-0.0088	0.0093	0.3460	-0.0264	0.0098
Current health expenditure per capita, PPP (current international $)	0.0000	0.0000	0.2010	0.0000	0.0000
Current health expenditure (% of GDP)	0.6636	0.1915	0.0010	0.2918	1.0372
External health expenditure (% of CHE)	0.0574	0.0725	0.4280	-0.0859	0.2020
Out-of-pocket expenditure (% of CHE)	-0.0338	0.0245	0.1670	-0.0838	0.0121
Population total (log)	0.0000	0.0000	0.0000	0.0000	0.0000
Population per km2	0.0000	0.0000	0.3810	0.0000	0.0000
Rural population (% of total population)	0.1030	0.0229	0.0000	0.0593	0.1507
TB spending accounted for by external sources (% of total TB spending, excluding private spending)	-0.0721	0.0178	0.0000	-0.1079	-0.0383
TB incidence per 100k	-0.0004	0.0000	0.0000	-0.0005	-0.0004
MDR-TB incidence per 100k	-0.0041	0.0006	0.0000	-0.0054	-0.0029
UHC service coverage index	0.2165	0.0651	0.0010	0.0913	0.3442
Population living in slums (% of urban population)	0.1766	0.0355	0.0000	0.1062	0.2465
Diabetes prevalence (% of population aged >= 18 years)	-0.0033	0.1199	0.9780	-0.2330	0.2268
Alcohol use disorders, 12-month prevalence (% of population aged >= 15 years)	-0.0113	0.1325	0.9320	-0.2666	0.2567
GINI index (0=perfect equality, 100=perfect inequality)	0.5938	0.0702	0.0000	0.4587	0.7344
Population living below the international poverty line (% of population)	-0.3414	0.0741	0.0000	-0.4827	-0.1902
Prevalence of undernourishment (% of population)	-0.7058	0.0780	0.0000	-0.8569	-0.5482
HIV prevalence (adults 15-49)	0.3054	0.1636	0.0620	-0.0186	0.6310
